# Clinical and health inequality risk factors for non-COVID-related sepsis during the global COVID-19 pandemic: a national case-control and cohort study

**DOI:** 10.1016/j.eclinm.2023.102321

**Published:** 2023-11-23

**Authors:** Xiaomin Zhong, Diane Ashiru-Oredope, Alexander Pate, Glen P. Martin, Anita Sharma, Paul Dark, Tim Felton, Claire Lake, Brian MacKenna, Amir Mehrkar, Sebastian C.J. Bacon, Jon Massey, Peter Inglesby, Ben Goldacre, The OpenSAFELY Collaborative, Alex J. Walker, Brian MacKenna, Peter Inglesby, Ben Goldacre, Helen J. Curtis, Jessica Morley, Amir Mehrkar, Sebastian C.J. Bacon, George Hickman, Richard Croker, David Evans, Tom Ward, Nicholas J. DeVito, Louis Fisher, Amelia C.A. Green, Jon Massey, Rebecca M. Smith, William J. Hulme, Simon Davy, Colm D. Andrews, Lisa E.M. Hopcroft, Iain Dillingham, Rose Higgins, Christine Cunningham, Milan Wiedemann, Linda Nab, Steven Maude, Orla Macdonald, Ben F.C. Butler-Cole, Thomas O'Dwyer, Catherine L. Stables, Christopher Wood, Andrew D. Brown, Victoria Speed, Lucy Bridges, Andrea L. Schaffer, Caroline E. Walters, Christopher Bates, Jonathan Cockburn, John Parry, Frank Hester, Sam Harper, Kieran Hand, Sian Bladon, Neil Cunningham, Ellie Gilham, Colin S. Brown, Mariyam Mirfenderesky, Victoria Palin, Tjeerd Pieter van Staa

**Affiliations:** a Centre for Health Informatics, School of Health Sciences, Faculty of Biology, Medicine, and Health, The University of Manchester, M13 9PL, UK; b Healthcare-Associated Infection (HCAI), Fungal, Antimicrobial Resistance (AMR), Antimicrobial Use (AMU) & Sepsis Division, United Kingdom Health Security Agency (UKHSA), London SW1P 3JR, UK; c School of Pharmacy, University of Nottingham, Nottingham NG7 2RD, UK; d Chadderton South Health Centre, Eaves Lane, Chadderton, Oldham OL9 8RG, UK; e Division of Infection, Immunity and Respiratory Medicine, Faculty of Biology, Medicine and Health, The University of Manchester, Manchester Academic Health Science Centre, Manchester, UK; f Intensive Care Unit, Manchester University NHS Foundation Trust, Wythenshawe Hospital, Manchester, UK; g Maples Medical Centre, 2 Scout Dr, Baguley, Manchester M23 2SY, UK; h Bennett Institute for Applied Data Science, Nuffield Department of Primary Care Health Sciences, University of Oxford, OX2 6GG, UK; i NHS England, Wellington House, Waterloo Road, London SE1 8UG, UK; j Pharmacy Department, Portsmouth Hospitals University NHS Trust, Portsmouth, UK; k NIHR Health Protection Unit in Healthcare-Associated Infection & Antimicrobial Resistance, Imperial College London, London, UK; l Division of Developmental Biology and Medicine, Maternal and Fetal Research Centre, The University of Manchester, St Marys Hospital, Oxford Road, Manchester M13 9WL, UK

**Keywords:** Health inequality, Morbidity, Primary care, Deprivation, Sepsis, COVID-19 pandemic

## Abstract

**Background:**

Sepsis, characterised by significant morbidity and mortality, is intricately linked to socioeconomic disparities and pre-admission clinical histories. This study aspires to elucidate the association between non-COVID-19 related sepsis and health inequality risk factors amidst the pandemic in England, with a secondary focus on their association with 30-day sepsis mortality.

**Methods:**

With the approval of NHS England, we harnessed the OpenSAFELY platform to execute a cohort study and a 1:6 matched case-control study. A sepsis diagnosis was identified from the incident hospital admissions record using ICD-10 codes. This encompassed 248,767 cases with non-COVID-19 sepsis from a cohort of 22.0 million individuals spanning January 1, 2019, to June 31, 2022. Socioeconomic deprivation was gauged using the Index of Multiple Deprivation score, reflecting indicators like income, employment, and education. Hospitalisation-related sepsis diagnoses were categorised as community-acquired or hospital-acquired. Cases were matched to controls who had no recorded diagnosis of sepsis, based on age (stepwise), sex, and calendar month. The eligibility criteria for controls were established primarily on the absence of a recorded sepsis diagnosis. Associations between potential predictors and odds of developing non-COVID-19 sepsis underwent assessment through conditional logistic regression models, with multivariable regression determining odds ratios (ORs) for 30-day mortality.

**Findings:**

The study included 224,361 (10.2%) cases with non-COVID-19 sepsis and 1,346,166 matched controls. The most socioeconomic deprived quintile was associated with higher odds of developing non-COVID-19 sepsis than the least deprived quintile (crude OR 1.80 [95% CI 1.77–1.83]). Other risk factors (after adjusting comorbidities) such as learning disability (adjusted OR 3.53 [3.35–3.73]), chronic liver disease (adjusted OR 3.08 [2.97–3.19]), chronic kidney disease (stage 4: adjusted OR 2.62 [2.55–2.70], stage 5: adjusted OR 6.23 [5.81–6.69]), cancer, neurological disease, immunosuppressive conditions were also associated with developing non-COVID-19 sepsis. The incidence rate of non-COVID-19 sepsis decreased during the COVID-19 pandemic and rebounded to pre-pandemic levels (April 2021) after national lockdowns had been lifted. The 30-day mortality risk in cases with non-COVID-19 sepsis was higher for the most deprived quintile across all periods.

**Interpretation:**

Socioeconomic deprivation, comorbidity and learning disabilities were associated with an increased odds of developing non-COVID-19 related sepsis and 30-day mortality in England. This study highlights the need to improve the prevention of sepsis, including more precise targeting of antimicrobials to higher-risk patients.

**Funding:**

The UK Health Security Agency, 10.13039/501100023699Health Data Research UK, and 10.13039/501100000272National Institute for Health Research.

**Research in context**

**Evidence before this study**

Sepsis, a life-threatening condition precipitated by infection, accounts for a significant portion of global mortality each year. Prior to undertaking this study, a comprehensive search was conducted focusing on peer-reviewed journal articles published between January 1, 2010 and January 31, 2023. We utilized the Embase database (accessed through Ovid) for sourcing relevant studies. Separate searches were carried out using the following terms in the titles of articles: (sepsis or septic) in combination with one of the following groups of terms: (depriv∗ or socioeconomic or socio-economic or socio or social or SES or IMD or income or occupation or education) OR (race or racial or ethnic∗ or minorit∗) OR (urban∗ or rural or coast∗) OR (residen∗ or care home or nursing home or care facility or living or social care or drug∗ or alcohol or disabil∗ or vulnerab∗). We found that the number of studies directly investigating the correlation between health inequalities, including factors such as deprivation and ethnicity, and the onset and management of sepsis was limited. Moreover, many of the studies were small-scale and lacked uniformity in defining health inequalities. The overall evidence indicated a knowledge gap regarding the interplay between health inequalities and sepsis recognition and management, particularly at a nationwide scale.

**Added value of this study**

This study is distinctive in its nationwide scope and focus on the intersection of health inequalities and community-acquired sepsis. Notably, it is the first to analyse fluctuations in the incidence of non-COVID-19 sepsis before, during, and after the COVID-19 pandemic within a large, high-income population. Our results show that factors such as socio-economic deprivation and clinical conditions, specifically chronic kidney, and liver disease, contribute to an increased risk of non-COVID-19 sepsis and subsequent 30-day mortality, irrespective of the COVID-19 pandemic. Furthermore, a history of extensive antibiotic exposure was identified as an additional risk factor.

**Implications of all the available evidence**

The evidence gathered in this study emphasizes the necessity for better prevention of sepsis using risk prediction models that factor in chronic disease status, factors commonly associated with health inequalities including deprivation status, and learning disabilities, alongside severity of infection. More precise targeting of antimicrobials could significantly optimise the prevention of sepsis, without increasing the risk of antimicrobial resistance. The implications of this research underscore the need for integrated, targeted strategies to address these risk factors, ultimately aiming to reduce the incidence and mortality associated with sepsis.

## Introduction

Sepsis is a complex syndrome encompassing physiological, pathological, and biochemical abnormalities induced by infection, characterised by life-threatening organ dysfunction resulting from a dysregulated host response.^1^ Efforts to prevent infections, both in the community and healthcare settings, can reduce the incidence of sepsis.^2^^,^^3^ In 2017, there were an estimated 48.9 million incident cases of sepsis globally, resulting in 11.0 million deaths, equating to 19.7% of all deaths globally.^4^ The World Health Organisation has called on member states to strengthen their efforts in identifying, documenting, preventing, and treating sepsis.^5^

Mounting evidence regarding the correlation between health inequalities and poor health outcomes highlights the need to address such disparities as increasingly urgent.^6^^,^^7^ To address health inequalities, the national health service commissioning body, NHS England has introduced the "Core20PLUS5" initiative, aimed at enabling local and national actions to identify and reduce disparities in key areas. The initiative comprises three components: Core20, which refers to the most deprived 20% of individuals based on the Index of Multiple Deprivation (IMD) quintile^8^; the PLUS component, which focuses on population groups (such as ethnic minorities, those with learning disabilities or those with high morbidity risk), and the 5 component, which focuses on improving clinical outcomes in five defined clinical areas.^9^^,^^10^ This study will explore the relationship between sepsis and Core20PLUS5 components. Research has highlighted a link between heightened deprivation and an increased occurrence of sepsis,^11^^,^^12^ Such studies indicate that individuals from lower socioeconomic backgrounds face an elevated risk of both developing sepsis and experiencing mortality post-sepsis diagnosis.^13^, ^14^, ^15^, ^16^ However, a limited number of investigations have holistically assessed the interplay between socioeconomic status (SES) and clinical risk factors before a sepsis hospital admission.

By March 2023, the global number of confirmed COVID-19 cases had exceeded 676 million, with approximately 6.9 million reported deaths.^17^ Whilst much research and public health efforts have concentrated on preventing COVID-19 infection and reducing mortality, there is a growing need to understand the indirect impacts of the pandemic due to national lockdowns, social restrictions, and changes in healthcare delivery. The indirect impacts include the changing prevalence of other infectious diseases as well as other non-communicable disease.^18^ This exploratory study investigated the incidence of non-COVID-19 sepsis amidst the complexities arising during the COVID-19 pandemic. Our primary objectives were: (1) to examine the association between health inequalities, such as SES, and the odds of developing non-COVID-19 sepsis or 30-day mortality, and (2) to evaluate the association between various clinical characteristics and the odds of developing non-COVID-19 sepsis or 30-day mortality. Our analysis primarily centred on community-acquired sepsis, which accounts for approximately 70% of cases.^19^^,^^20^

## Methods

### Data source

The primary care records managed by GP software provider TPP were retrieved through the OpenSAFELY platform. All data were linked, stored, and analysed securely within the OpenSAFELY platform (https://opensafely.org/). Data include pseudonymised data on 23.4 million people.^21^ The primary care data was linked to the death data from UK Office for National Statistics, SARS-CoV-2 testing data from Second Generation Surveillance System (SGSS) and hospital secondary care records through the Secondary Uses Services (SUS). All data were linked, stored and analysed securely within the OpenSAFELY platform: https://opensafely.org/. Data include pseudonymised data such as coded diagnoses, medications and physiological parameters. No free text data are included. All code is shared openly for review and re-use under MIT open license (https://github.com/opensafely/amr-uom-brit). Detailed pseudonymised patient data is potentially re-identifiable and therefore not shared.

This study was approved by the Health Research Authority and NHS Research Ethics Committee [REC reference 21/SC/0287].

### Study design and participants

Records between 1st January 2019 and 30th June 2022 were analysed. Patients diagnosed with sepsis were identified using ICD-10 codes from the hospital admissions record based on existing study codelists (available in eTable 1).^22^, ^23^, ^24^ For each patient, the date of their sepsis diagnosis was defined as the index date. Patients were excluded if they were not registered a primary care practice for at least one-year prior to the index date. For patients with more than one sepsis admission in the study period, their first episode only was selected for the analysis. Cases without a record of index of multiple deprivation (IMD) or region recorded were excluded. The non-COVID-19 sepsis cohort was defined as a sepsis diagnosis without a COVID-19 infection record from primary or secondary care six weeks before/after index date (eFigure 1).

A case-control study was conducted. Cases were all individuals in the cohort defined above. Controls included patients without any recorded diagnosis of sepsis, satisfying the other inclusion criteria (detailed criteria on potential controls can be found in eText 1). Cases were matched 1:6 to control on age (stepwise), sex, and calendar month. The initial step in the matching process was pairing cases with potential controls on exact age and broaden to a maximum ± five-year age interval until each case had a total of six controls (See eFigure 1 for the study design). This study has been diligently reported, adhering to the Strengthening the Reporting of Observational Studies in Epidemiology (STROBE) guideline.

### Outcomes

The primary outcome was a non-COVID-19 sepsis diagnosis during admission. Community-acquired sepsis was defined as a patient who had a sepsis diagnosis within the first two days of the hospital admission.^25^^,^^26^ If the patient’s sepsis episode started more than two days after hospital admission, it was categorised as hospital-acquired sepsis.^19^^,^^27^ A secondary outcome was 30-day-mortality in patients with sepsis (i.e., death record for any reason 30 days after sepsis diagnosis).

### Exposures

The primary exposure variable was socioeconomic deprivation, assessed by IMD quintile (1–5) (See eText 2). IMD score incorporates information on income, employment, crime rate, living environment, education, and barriers to services. Other exposure variables included multiple demographics and clinical factors. These were selected based on the Core20PLUS5, risk predictors highlighted by the National Institute for Health and Care Excellence (NICE), and findings from previous studies.^9^^,^^11^^,^^12^^,^^16^^,^^28^^,^^29^ We defined two sets of predictors, and a full list of variables and definitions can be found in eText 3. All comorbidities were extracted from patient records on, or before, the index date, further information of comorbidity definition and codelists can be found in the eTable 1. The COVID-19 pandemic was viewed as a potential effect modifier in this study. We defined a categorical variable with three calendar time periods: (1) before COVID-19: 2019-01-01 to 2020-03-25 (2) implementation of national lockdown: 2020-03-26 to 2021-03-08 (3) after national lockdown: 2021-03-09 to 2022-06-30. We interacted this categorical variable with exposure variables to examine potential effect modification.

### Statistical analysis

To ascertain changes in the incidence of non-COVID-19 sepsis, descriptive analysis assessed the changes in the incidence of cases with new non-COVID-19 sepsis in hospitalisation before and after COVID-19. The incidence rate was defined as the monthly count of cases with new non-COVID-19 sepsis per 1000 patients registered. To examine the different odds of developing non-COVID 19 sepsis in different groups of IMD (see eText 2 for definition), ethnicity, BMI, and smoking status (the first set of predictors, see eText 3 for definition), Conditional logistic regression models were utilised to gauge the association between specific risk factors and the odds of developing non-COVID-19 sepsis, with results articulated as odds ratios (ORs) alongside their 95% confidence intervals (95% CI). We assessed the unadjusted effects of these predictors, deliberately not adjusting for diseases potentially resulting directly from these variables. Given the possibility that certain diseases may mediate these associations (as illustrated in eFigure 15, for instance, diabetes might elevate sepsis risk and lower IMD could be linked with a reduced diabetes risk, adjusting for disease when analysing the influence of IMD on sepsis may nullify certain effects), we sequentially fitted four models from unadjusted to fully adjusted, as detailed in eFigure 4. When considering our secondary set of variables (encompassing clinical characteristics and prior antibiotic prescription counts, see eText 3 for definition), we employed two distinct conditional logistic regression models: one crude (unadjusted) model and another adjusting for all other variables in this secondary set, offering insight into the incremental influence of disease.

We conducted a descriptive analysis examining 30-day mortality following a non-COVID-19 sepsis diagnosis across various subgroups and distinct periods (eTables 6 and 7). Within cases with non-COVID-19 sepsis, we fitted separate logistic regression models in each COVID-19 period. Changes in health-seeking behaviours, healthcare delivery, and potential interaction of non-COVID-19 sepsis risk factors with COVID-19-related changes led us to investigate the impact on specific subgroups. For each period, and for each clinical and demographic covariate of interest, we applied a logistic regression model to better understand the pandemic's diverse impacts on these specific groups. The outcome was a binary variable indicating death or not within 30 days. For all the variables listed in exposure and secondary exposure in previous section, we fitted the model and adjusted by age (restricted cubic splines with 4 knots), sex and stratified by region. We calculated the relative ORs in different periods (ORs (Period 2 vs 1) and ORs (Period 3 vs 1)) to compare the changes in non-COVID-19 sepsis mortality before/during and after the peak of the COVID-19 pandemic. Additionally, fully adjusted models were fitted by adjusting for all comorbidities (the variables listed in second set in eText 2, eFigures 12–14). Additionally, we also fitted the models across three periods and the specific COVID-19 time periods were assumed to be a moderator variable and included in each model for the analysis of potential heterogeneity of the risk factor effects over time due to the pandemic (See eTable 13).

### Missing data

There were missing data for body mass index (BMI), smoking history, and ethnicity. In an initial (primary) approach these missing values were treated as a separate category in the regression analyses, an approach known as the ‘missing indicator' method. This approach is not based on statistical theory for missing data. However, we note that whilst multiple imputation is typically more appropriate, it may not be well suited to our context because previous studies concerning documentation in UK primary care records indicated violation of the ‘missing at random' assumption. For instance, individuals who are underweight or overweight are more likely to have their BMI documented in primary care (an example of data ‘missing not at random'). These were categorised as “Unknown” in the regression analyses. No data were missing for comorbidities; they were coded as either present or absent. Previous studies concerning their documentation in UK primary care records indicated that employing multiple imputation wouldn’t be suitable due to the violation of the missing at random assumption. For instance, individuals who are underweight or overweight are more likely to have their BMI documented in primary care.^30^

Density plots were used to check the matching process for age between cases and controls. We undertook three sensitivity analyses. Firstly, BMI was missing in all patients <18 years old and therefore the adult group was analysed separately. Secondly, multiple imputation was applied, generating five imputed datasets distinctively for all age cohort and adult patients only, incorporating missing variables through the multinomial regression model that encompassed all covariates and outcome indicators. Subsequent combination of estimates adhered to Rubin’s rules (eTables 15–19).

Thirdly, given that the missing data in primary care is attributed to specific reasons and cannot be simply assumed to be missing at random, a complete case analysis was conducted.^31^, ^32^, ^33^, ^34^ When data are missing not at random, complete case analysis might be less biased than multiple imputation (eTables 9–12).^35^

Data management and analysis was performed using Python 3.9.1 and R 4.0.2. All analysis code and codelists used are archived online (https://github.com/opensafely/amr-uom-brit/tree/sepsis). The published output can also be found online (https://jobs.opensafely.org/university-of-manchester/brit-antibiotic-research/sepsis_hosp_admission/logs/). The OpenSAFELY research platform adheres to the obligations of the UK General Data Protection Regulation (GDPR) and the Data Protection Act 2018. In March 2020, the Secretary of State for Health and Social Care used powers under the UK Health Service (Control of Patient Information) Regulations 2002 (COPI) to require organisations to process confidential patient information for the purposes of protecting public health, providing healthcare services to the public and monitoring and managing the COVID-19 outbreak and incidents of exposure; this sets aside the requirement for patient consent.^36^

### Role of the funding source

The funder of the study had no role in study design, data collection, data analysis, data interpretation, or writing of the report. XZ, VP, JM, PI, BM, AM, SB, TvS had access to dataset and TvS had final responsibility for the decision to submit for publication.

## Results

**Fig. 1:**
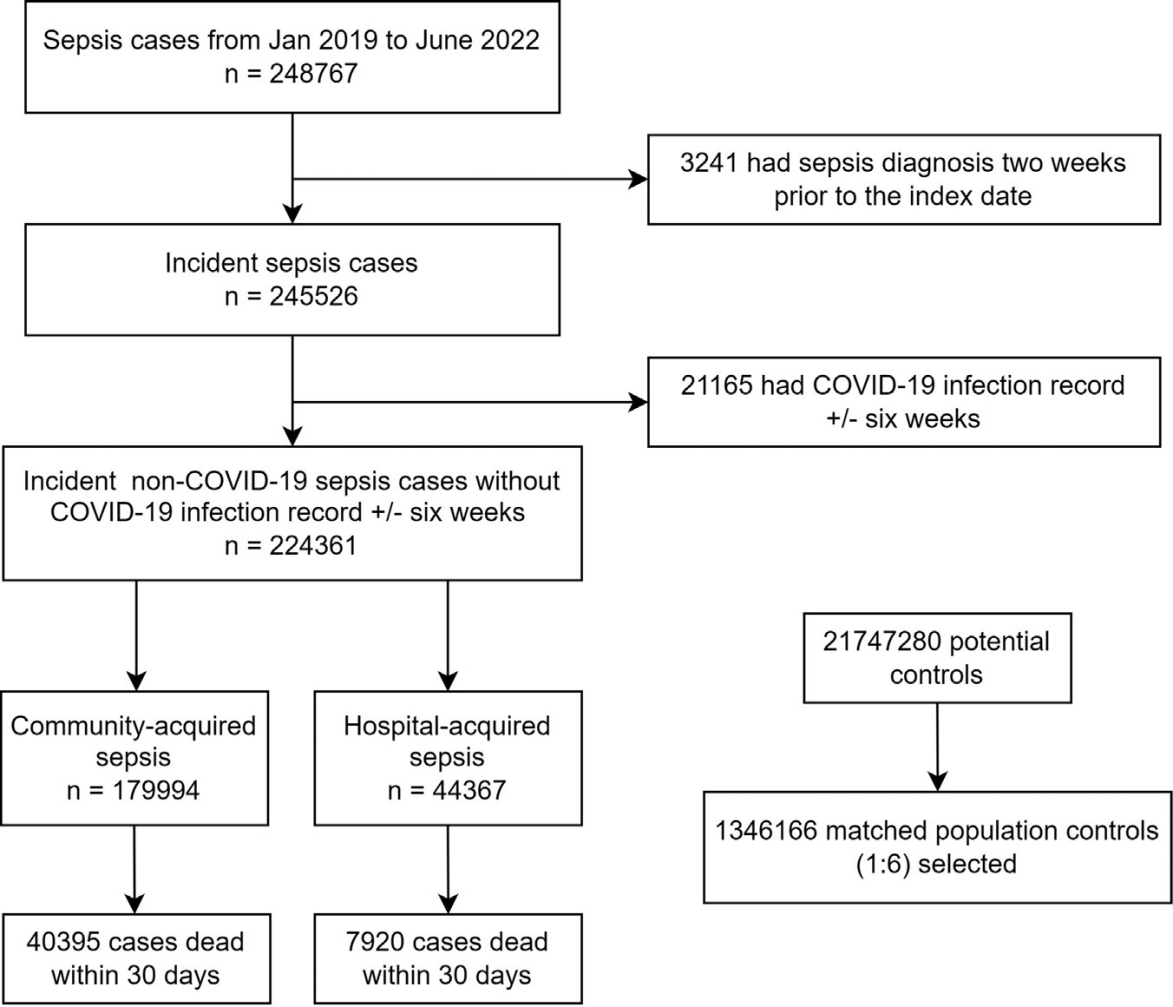
**Study flowchart.**

Between January 1, 2019 and June 31, 2022, there were 248,767 (11.3%) cases with non-COVID-19 sepsis from a cohort of 22.0 million individuals. 224,361 (10.2%) were eligible cases with incident non-COVID-19 sepsis (79.8% community-acquired, and 20.2% hospital-acquired). After matching, 1,346,166 eligible controls were found (Fig. 1).

The ICD-10 code for sepsis can be accessed directly via this link: [https://codelists.opensafely.org/codelist/user/BillyZhongUOM/codes_for_sepsis/00ab8f66/].

The baseline characteristics of the patients are shown in Table 1 (additional characteristics in eTables 2 and 3). A higher proportion of cases were of white ethnicity, and living with overweight or obesity (eText 3), came from the most deprived quintile, had a smoking history, or with hazardous alcohol drinking behaviour. The incidence of non-COVID-19 sepsis was greater in babies, low from age 3 to 17 and then steeply increased with higher age (eFigure 2). The incidence was higher in males during periods 1 and 3, but the values dropped to similar levels during the national lockdown (eTable 4).

**Table 1:** Baseline characteristics for cases with non-COVID-19 sepsis and controls.

	Cases		Controls	
**Age**				
Mean (SD)	69.6	19.3	69.6	19.3
	N	%	N	%
**Age groups**				
<18	4660	2.1	27,970	2.1
18–39	16,120	7.2	96,720	7.2
40–49	10,495	4.7	62,960	4.7
50–59	21,075	9.4	126,455	9.4
60–69	34,385	15.3	206,300	15.3
70–79	57,085	25.4	342,505	25.4
80+	80,545	35.9	483,260	35.9
**Sex**				
Female	108,935	48.6	653,600	48.6
Male	115,425	51.4	692,525	51.4
**Region**				
North East	10,275	4.6	63,645	4.7
North West	21,080	9.4	122,035	9.1
Yorkshire and the Humber	30,930	13.8	183,675	13.6
East Midlands	49,105	21.9	234,045	17.4
West Midlands	9040	4.0	49,820	3.7
East of England	52,080	23.2	315,410	23.4
London	10,530	4.7	59,815	4.4
South East	15,650	7.0	97,835	7.3
South West	25,660	11.4	219,890	16.3
**IMD quintile**^a^				
5 (least deprived)	37,735	16.8	292,047	21.7
4	44,320	19.8	301,378	22.4
3	48,700	21.7	302,716	22.4
2	46,030	20.5	242,459	18.0
1 (most deprived)	47,575	21.2	206,509	15.4
**Ethnicity**^b^				
White	205,900	91.8	1,170,750	87.0
Mixed	1430	0.6	8315	0.6
South Asian	9720	4.3	50,250	3.7
Black	2975	1.3	17,465	1.3
Other	2100	0.9	14,565	1.1
Unknown	2235	1.0	84,820	6.3
**BMI**^c^				
Healthy range (18.5–24.9 kg/m^2^)	63,910	28.5	379,745	28.2
Underweight (<18.5 kg/m^2^)	11,235	5.0	38,980	2.9
Overweight (25–29.9 kg/m^2^)	57,025	25.4	390,220	29.0
Obese I (30–34.9 kg/m^2^)	32,630	14.5	185,420	13.8
Obese II (35–39.9 kg/m^2^)	14,565	6.5	63,140	4.7
Obese III (40+ kg/m^2^)	11,405	5.1	30,980	2.3
Unknown	33,590	15.0	257,680	19.1
**Smoking status**^d^				
Never	74,360	33.1	547,190	40.6
Former	113,555	50.6	627,575	46.6
Current	31,075	13.9	129,430	9.6
Unknown	5365	2.4	41,970	3.1

To reduce the risk of secondary disclosure, all counted numbers in the baseline table were rounded to the nearest five.

a IMD (Index of Multiple Deprivation) quintile measured from patient-level address.

b Ethnicity in line with 2001 Census categories.

c BMI, body mass index groups based on the NICE definitions.

d Smoking status identified from the most recent clinical records.

**Fig. 2:**
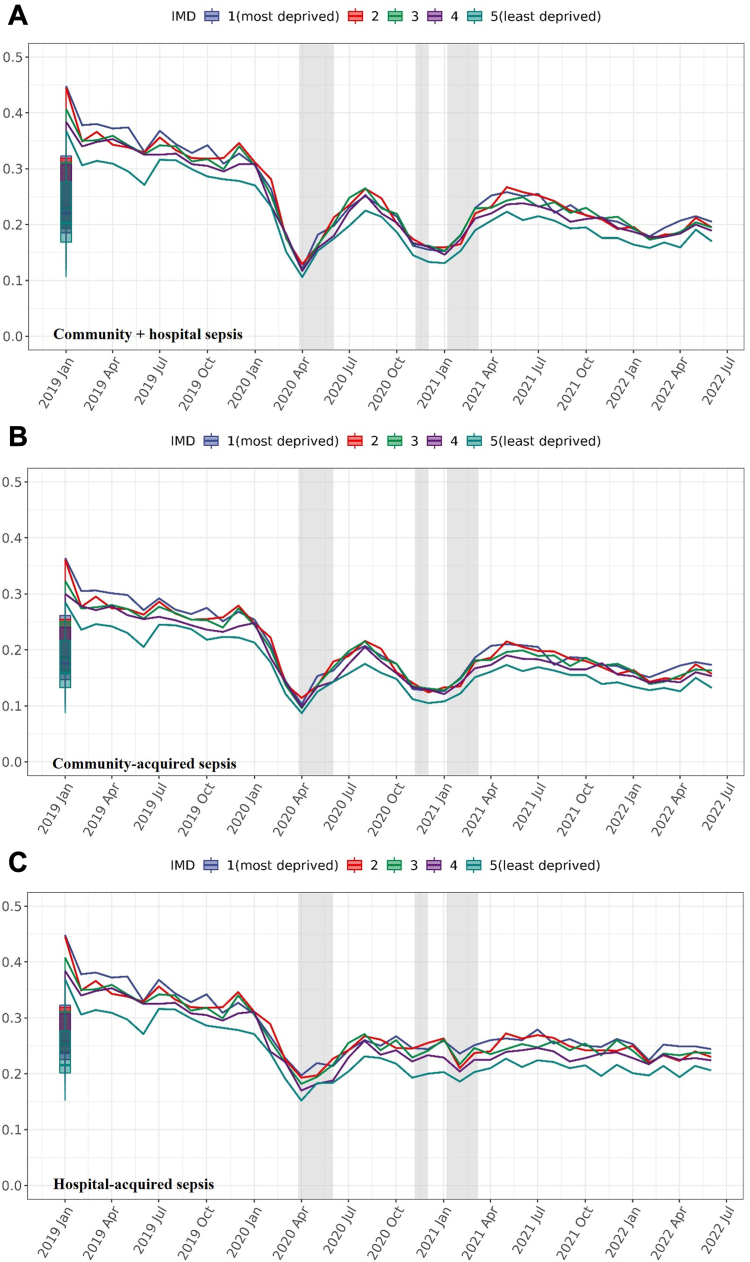
**Incidence rates of non-COVID-19 sepsis over time (calculated every month based on the number of new cases per 1000 registered persons).** IMD (Index of Multiple Deprivation) quintile measured from patient-level address. Numerator is the number of cases with sepsis (times 1000), and the denominator is the number of all baseline population, grouped by IMD quintiles. Boxplots represent the historical average (median and IQR) percentage of incidence rates of cases with new non-COVID-19 sepsis from January 2019 to June 2022. The shadow area indicating the periods of national lockdown. See underlying numbers in eTable 14.

In the incidence trend analysis, the monthly non-COVID-19 sepsis diagnosis rate dropped from 0.3 per 1000 registered person in February 2020 to 0.1 in April 2020 (compared to 0.4–0.35 in 2019). The rate fluctuated until April 2021 and then remained stable until the study end (Fig. 2). The least deprived quintile had the lowest risk of developing non-COVID-19 sepsis across all periods.

### Analysis of developing non-COVID-19 sepsis

**Fig. 3:**
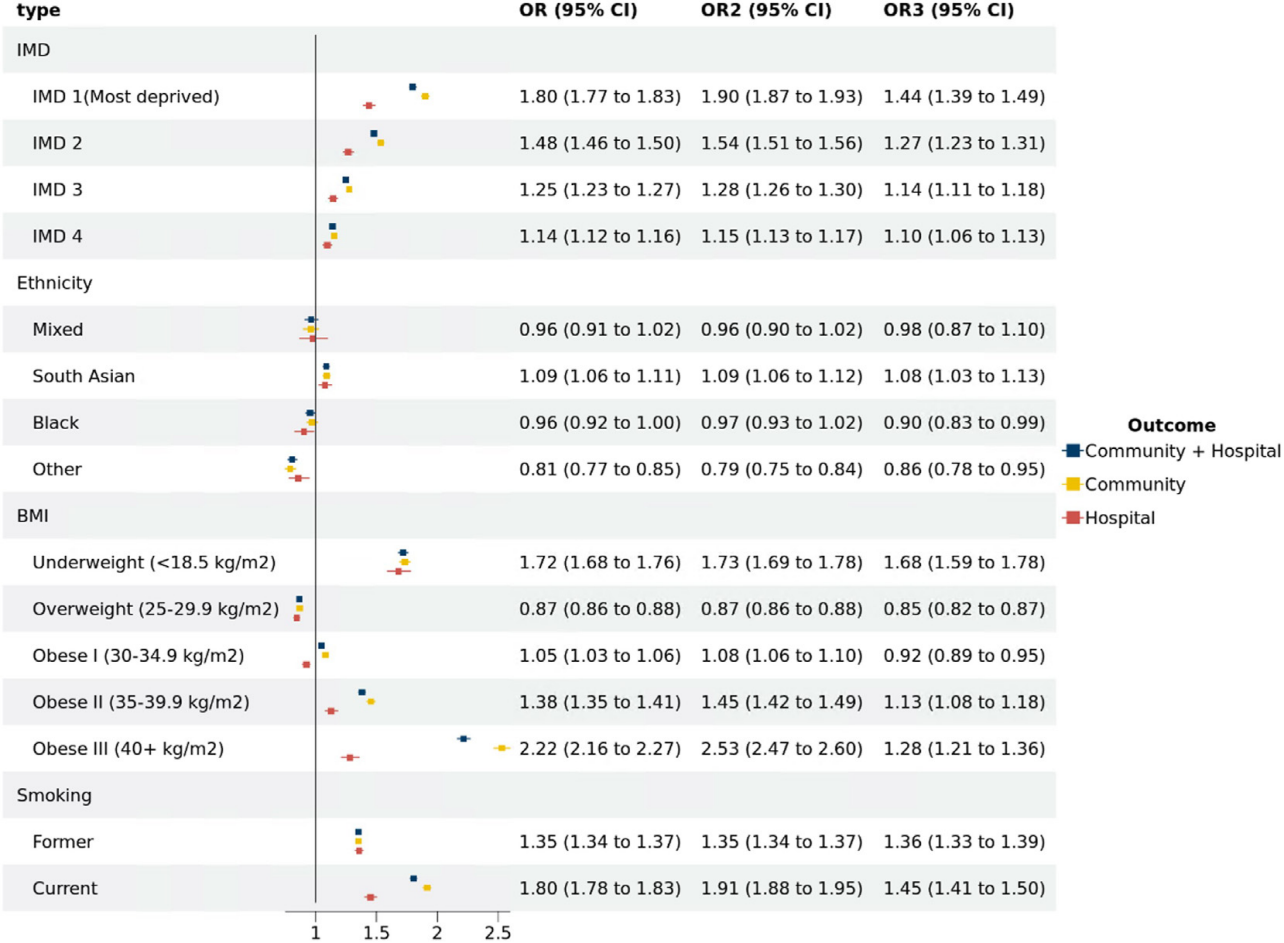
**ORs of developing non-COVID-19 sepsis for factors stratified by sepsis type.** Crude ORs of sepsis by IMD quintile, ethnicity, BMI, smoking history and stratified by type of sepsis. OR: Community + Hospital, OR2: Community, OR3: Hospital. Reference groups: IMD quintile: the least deprived quintile (IMD 5). Ethnicity: white. BMI: healthy range (18.5–24.9 kg/m^2^). Smoking: never (Smoking status identified from the most recent clinical records). Abbreviations: IMD, index of multiple deprivation (quintile measured from patient-level address); BMI, body mass index (from the most recent clinical records).

In analyses of the unadjusted model, the most deprived quintiles were associated with higher odds of developing non-COVID-19 sepsis (Fig. 3) (OR [95% CI] for IMD1 (most deprived) 1.80 [1.77–1.83], IMD 2 1.48 [1.46–1.50], IMD3 1.25 [1.23–1.27], IMD4 1.14 [1.12–1.16]). The OR in community-acquired non-COVID-19 sepsis was greater than hospital-acquired for the most deprived IMD quintile at 1.90 [95% CI 1.87–1.93] and 1.44 [95% CI 1.39–1.49], respectively. In fully adjusted models including all comorbidities, there was modest attenuation of the association between the most deprived quintile and odds of non-COVID-19 sepsis, with the OR still 1.4-fold higher than the least deprived group (eFigure 4). We observed no discernible moderating effect of the COVID-19 periods on the association between different IMD quintile and the odds of developing non-COVID-19 sepsis.

**Fig. 4:**
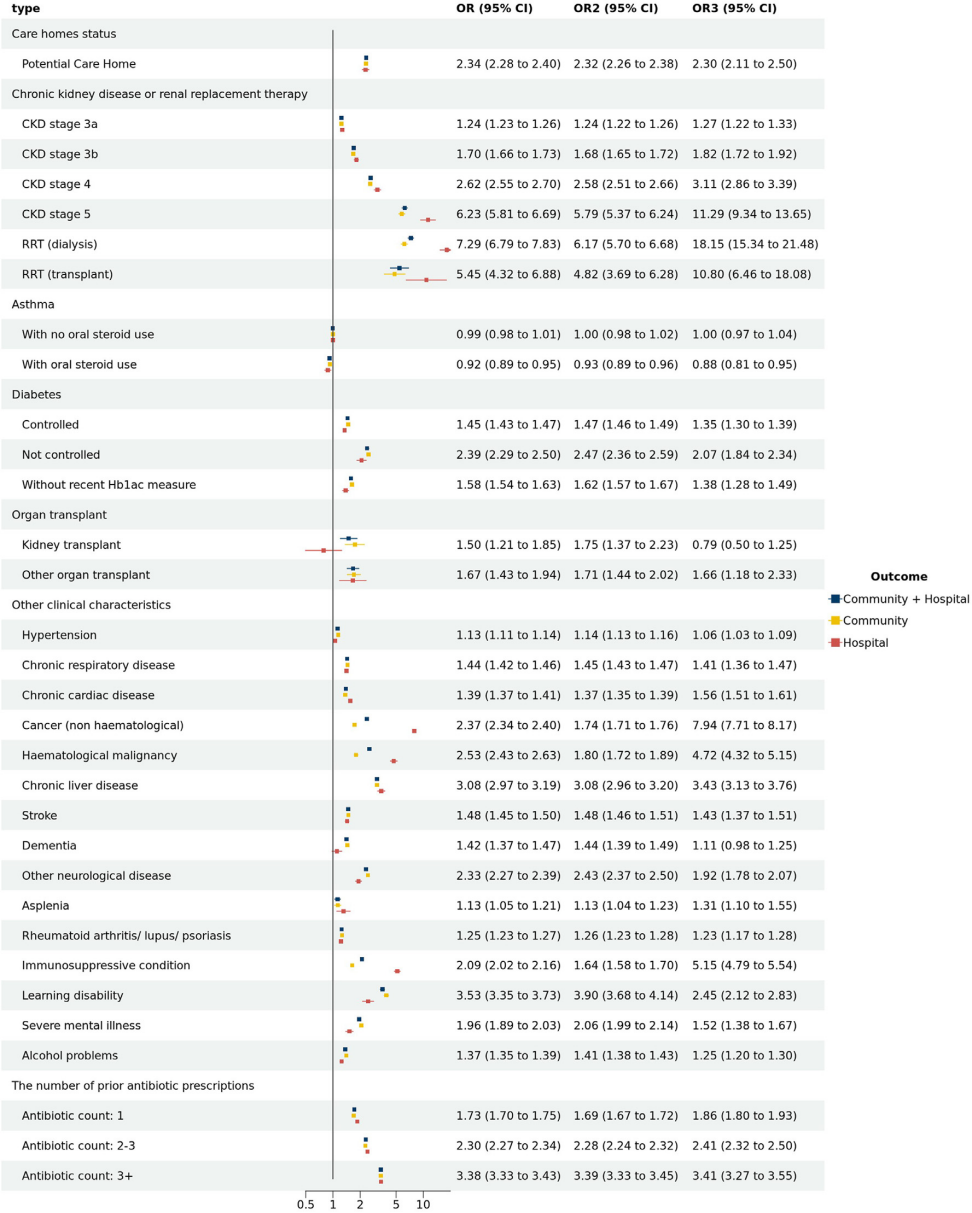
**Adjusted ORs of developing non-COVID-19 sepsis for clinical characteristics stratified by sepsis type.** Models were adjusted for all comorbidities∗. OR: Community + Hospital, OR2: Community, OR3: Hospital. Reference groups: Clinical characteristics: the patients without the clinical disease. The number of prior antibiotic prescriptions: antibiotic count: 0. Models were adjusted for all comorbidities∗. All comorbidities∗ Hypertension, chronic cardiac disease, diabetes, stroke, chronic kidney disease or renal replacement therapy and asthma, cancer (non-haematological and haematological), chronic liver disease, dementia, other neurological disease (including motor neuron disease, myasthenia gravis, multiple sclerosis, Parkinson's disease, cerebral palsy, quadriplegia or hemiplegia, and progressive cerebellar disease), organ kidney transplant, asplenia (due to splenectomy or spleen dysfunction, including sickle cell disease), rheumatoid arthritis/lupus/psoriasis, other immunosuppressive conditions, learning disability, several mental ill, the number of prior antibiotic prescription from one year and six week to six week before the index time (indicating the infection history). Abbreviations: CKD, chronic kidney disease; RRT, renal replacement therapy. The number of prior antibiotic prescriptions (one year plus six weeks to six weeks before the index date), The ORs for the number of antibiotics given within six weeks before the index date can be found in eFigure 5.

Individuals of South Asian descent were observed to have a higher incidence of sepsis, while those categorised under the ‘other' ethnic groups demonstrated a reduced incidence compared to individuals of white descent (Fig. 3). Patients living with underweight or obesity showed higher odds of developing non-COVID-19 sepsis. We also found higher odds of non-COVID-19 sepsis in patients with smoking history (Fig. 3), potential care home status, chronic kidney disease (CKD) or Renal Replacement Therapy (RRT) and organ transplantation. Other diseases, including diabetes (not controlled), malignancy (haematological and non-haematological), chronic liver disease, other neurological diseases, immunosuppressive condition, and learning disabilities had adjusted ORs greater than 2 (See Fig. 4, and same trend in crude ORs in eFigure 8). Patients with an antibiotic prescription within the last year had an adjusted ORs for community-acquired non-COVID-19 sepsis of 3.39 [95% CI 3.33–3.45] (crude OR was 5.13 [95% CI 5.05–5.21]). In eTable 5, the discriminatory capacity of conditional logistic models for clinical characteristics is shown. The model's efficacy in discerning community-acquired non-COVID-19 sepsis from control cases was quantified by a c-statistic value of 0.753. We found the result matched properly in the sensitivity analysis for 18+ study population (eFigures 6–9). The complete case analysis (eTables 9–12) and the analysis using multiple imputation (eTables 15–19) also presented a consistent result (see further statement in eText 6).

### Analysis of non-COVID-19 sepsis mortality

**Fig. 5:**
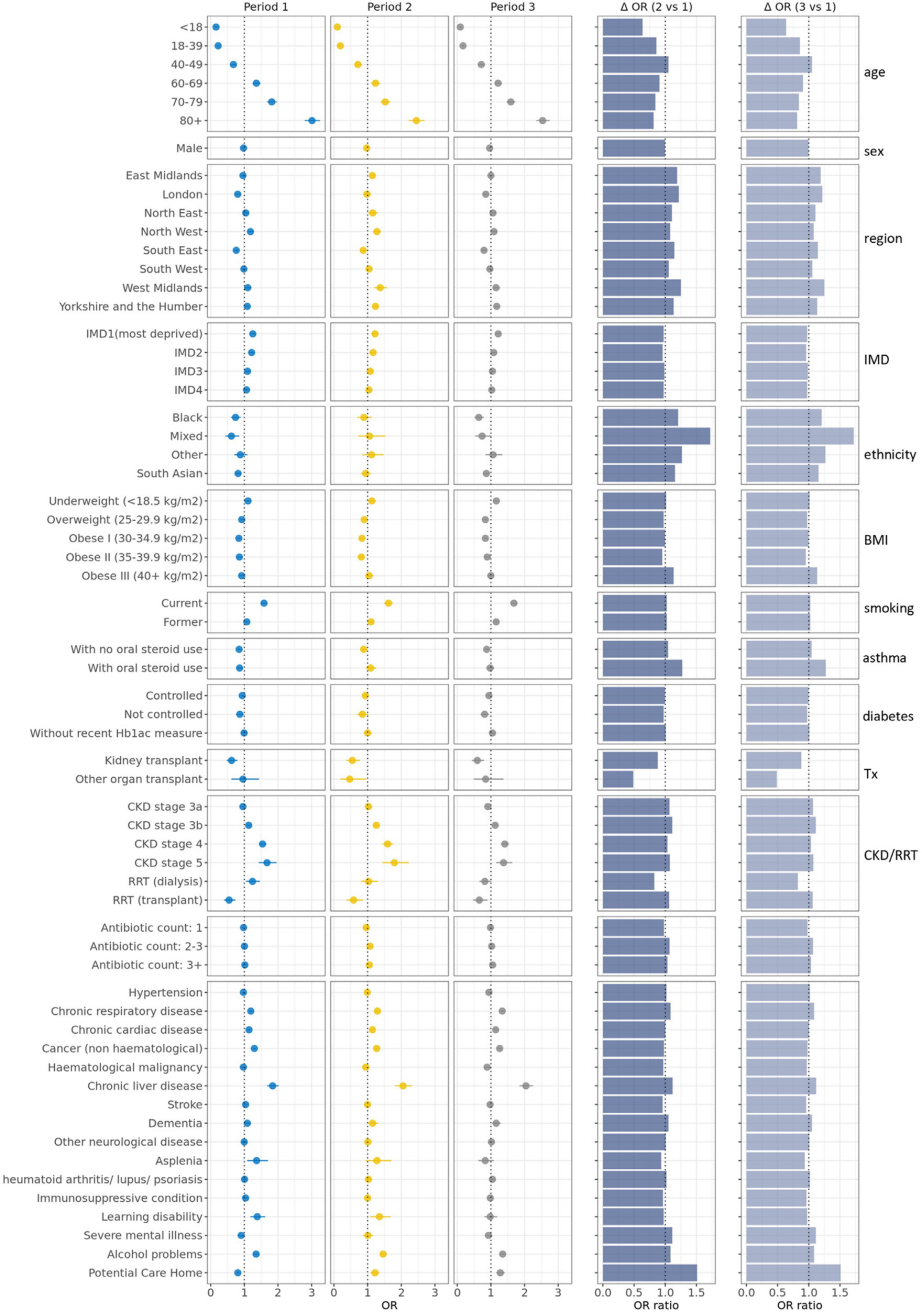
**Adjusted ORs of community-acquired non-COVID-19 sepsis 30-day mortality stratified by COVID-19 period.** Relative odds ratio of 30-day mortality after sepsis and 95% confidence intervals in OpenSAFELY-TPP in the three periods (Period 1: 2019-01-01 to 2020-03-25; Period 2: 2020-03-26 to 2021-03-08; Period 3: 2021-03-09 to 2022-06-30). Models were adjusted for age using a 4-knot restricted cubic spline, except for estimation of age group relative odds of 30-day mortality; and adjusted for sex, except for estimation of sex group relative odds of 30-day mortality; and stratified by region, except for IMD group relative odds of 30-day mortality. The two columns on the right present the ratio of the relative odds of 30-day mortality (fold-change: Δ OR) of period 2 vs 1 and period 3 vs 1. Abbreviations: IMD, index of multiple deprivation; BMI, body mass index; Tx, transplant; CKD, chronic kidney disease; RRT, renal replacement therapy. The relative odds of 30-day mortality presented in this figure can be found in eTable 8 of the Supplementary Material. The number of prior antibiotic prescriptions (one year plus six weeks to six weeks before the index date). Reference sub-group: age: 50–59 years, sex: female, region: East of England, IMD: the least deprived quintile, ethnicity: white, BMI: healthy range (18.5–24.9 kg/m^2^), smoking: never, prior antibiotic count: 0. Patients without the disease were used as the reference for other clinical conditions. The ORs and relative ORs can be found in eTable 8 in the supplement.

In community-acquired non-COVID-19 sepsis, the 30-day mortality was highest in the 80 years of age and over group, and patients of white ethnicity had the highest mortality (eTables 6 and 7). In Fig. 5, in most of the variables of interest subgroups, the relative adjusted OR of death 30 days after community-acquired non-COVID-19 sepsis diagnosis stayed comparably constant across the successive period. The adjusted OR of mortality for the most deprived quintile was 1.25 [95% CI 1.18–1.33] in period 1, decreased to 1.22 [95% CI 1.13–1.32] in period 2 and 1.12 [95% CI 1.14–1.30] in period 3. The ORs in fully adjusted model with all comorbidities also matched the adjusted OR above (eFigure 13). Additionally, when recognising the specific COVID-19 time periods as a modulating variable, the conclusions drawn remained in harmony with our primary findings (eTable 13).

## Discussion

Sepsis remains a global issue of significant concern. Understanding clinical and health inequality risk factors for sepsis remains essential to overall understanding of at-risk cohorts and effective public health mitigations. The analysis showed that non-COVID-19 sepsis diagnosis rate dropped significantly during the periods of national lockdown, fluctuated in the interim periods and returned to pre-pandemic levels after April 2021. Before the pandemic, more deprived subgroups had higher rates of non-COVID-19 sepsis, especially for community-acquired non-COVID-19 sepsis. The presence of several clinical characteristics including socioeconomic deprivation, underweight or obese, smoking history, potential care home status, CKD or RRT, organ transplantation, diabetes, malignancy (haematological and non-haematological), chronic liver disease, other neurological diseases, immunosuppressive condition, and learning disabilities increased this risk. No potential moderating effect of the COVID-19 regarding the association between risk factors and the odds of developing non-COVID-19 sepsis. The risk of mortality within 30 days of non-COVID-19 sepsis diagnosis was found to be moderately associated with deprivation, CKD, and chronic liver disease.

As the first nationwide investigation into health inequalities and the development of community-acquired sepsis, this study provides comprehensive data and findings of relevance to healthcare systems worldwide. It is the first study to analyse changes in non-COVID -19 sepsis incidence before, during and after the COVID-19 pandemic in a large high-income population. To date most published studies on sepsis have been hospital-based with analysis derived using hospital records, providing limited data on prior medical history and lacking population-based controls.^15^^,^^16^^,^^37^^,^^38^ A nationwide case-control study in Sweden found that low socio-economic status, psychiatric illness, substance abuse, and certain somatic co-morbidities (excluding myocardial infarction) were risk factors for ICU-admission for community-acquired sepsis. The Swedish study also highlighted the strongest risk factors were end-stage renal disease, liver disease, metastatic malignancy, substance abuse, and congestive heart failure, which is consistent with the present study.^12^ A global analysis conducted by Rudd et al. utilized data from 109 million death records to determine the mortality rates associated with sepsis and its correlation to underlying causes of death. The study revealed a notable difference in the cases vs deaths, particularly in regions with a lower socio-demographic index (SDI), where individuals may have a higher risk of mortality due to sepsis.^4^

Nearly half of all sepsis-related deaths occurred secondary to sepsis complicating an underlying injury or non-communicable disease.^4^^,^^39^ Recent research in Norway and Australia has explored the impact of socioeconomic status on the risk and mortality of sepsis.^13^^,^^27^ Consistently, these studies reveal that a lower SES correlates with increased sepsis risk and mortality. Another study from Australia underscores the persistent clinical challenges posed by sepsis/bacteraemia-related morbidity and mortality in patients with cirrhosis.^40^ In contrast to prior studies, our research encompasses a broader age range, utilises a more extensive national dataset, and provides a more comprehensive angle by considering both community-acquired and hospital-acquired non-COVID-19 sepsis. Furthermore, our work offers a novel viewpoint on this matter by contemplating the potential effects of the recent pandemic. The present study fills an important research gap on changes in the incidence of non-COVID-19 sepsis during the COVID-19 pandemic. Furthermore, whilst the study identifies that the pandemic did not have a significant impact on the relationship between specific risk factors and the diagnosis of non-COVID-19 sepsis and death, the rates of non-COVID-19 sepsis decreased during national lockdowns. The decrease in incidence of sepsis during this period could be attributable to reduced social mixing, or lack of ascertainment due to changes in healthcare delivery. The potential reason that we did not observe a moderating effect of the COVID-19 time periods regarding the association between risk factors and non-COVID-19 sepsis may be attributed to the overarching effects of the pandemic on the general populace, rather than isolated subgroups. Indeed, recent research elucidates that shifts in healthcare delivery—particularly antibiotic prescription patterns—remain consistent across various age, sex, IMD, and ethnicity groups.^18^^,^^41^ This further substantiates our premise that the pandemic's broader effects span across diverse population segments rather than only influencing specific subgroups.

NICE in England developed a guideline for the identification, diagnosis, and early management of sepsis in 2016 (2017 last updated).^28^ The Academy of Medical Royal Colleges also recently published a statement focusing on early diagnosis and management of sepsis and initial antimicrobial treatment.^39^ Although the NICE guideline lists risk factors for developing sepsis, it offers limited considerations and does not include information on their relative importance. Notably, the considerations of health inequalities is not considered in the statement from the Academy of Medical Royal Colleges.

The present study found that patients with multiple prior courses of antibiotics have higher risks of developing sepsis. One explanation could be underlying differences in immune status or underlying comorbidities predisposing to repeated infection. Another explanation could be adverse antibiotic effects on microbiota leading to increased susceptibility to infection.^42^ Given the potential adverse effects of (repeated) antibiotic courses, there is a need to target antibiotics to those patients who would most need and benefit from them.^43^^,^^44^ However, research has found that antibiotics are often not targeted appropriately.^45^ There is an urgent need for better risk prediction of infection-related complications, correlating clinical characteristics (including those reported in this study) with infection severity.

This observational study delineates the association between various factors and sepsis in the context of the COVID-19 pandemic. This study suggests better prevention strategies through the use of risk prediction models, and improved targeting of antimicrobial treatments. This study underscores the potential utility of tailored, patient-level clinical predictions incorporating individual demographic data and long-term conditions to enhance responsiveness to challenges such as sepsis. However, it is crucial to recognise that these findings are primarily descriptive and necessitate further research to establish causality and inform the development of effective prevention and treatment strategies for sepsis. Furthermore, by harnessing this knowledge, the healthcare system can be better equipped to face potential future pandemics or global health crises, ensuring that vulnerable groups are not disproportionately affected, and that the overall quality of care is maintained or even improved. This aligns with the objectives of the Core20PLUS5 approach, which aims to reduce healthcare inequalities by identifying target populations and clinical areas that require accelerated improvement.^9^^,^^10^ The Core20 population consists of those who are most deprived, which overlaps with the high comorbidity burden and low socio-economic status group of patients in this study. Additionally, the PLUS population groups identified by the Core20PLUS5 approach include those experiencing social exclusion, such as homeless individuals and those with drug and alcohol dependence, who are also at high risk for sepsis.

Our study has limitations. We did not collect information on the number of people in a household, occupation, availability of personal protective material, and adherence to social distancing measures, which could impact exposure risk and confound our results. An inherent limitation of our study stems from the utilisation of ICD-10 codes to identify cases with sepsis. Relying on such codes can both underestimate and overestimate sepsis incidence across different populations when juxtaposed against the backdrop of clinical observations data and established criteria such as SIRS or sepsis-3.^46^, ^47^, ^48^ Unfortunately, the nature of the OpenSAFELY platform restricts our ability to directly access comprehensive clinical data for sepsis identification. In this study, the missing data mechanism is most likely missing not at random (MNAR) due to ‘informative observations'. There is no robust method to handle MNAR data, we expect some residual bias both when using the missing indicator method and in our sensitivity analyses. Furthermore, the MNAR nature complicates the use of multiple imputation. To enhance the reliability of our results, we undertook additional analyses using multiple imputation and complete case analysis and focused on the 18+ age group. No major differences were observed between the different missingness approach in the ORs of developing non-COVID-19 sepsis with the variables of interest. Additionally, as an observational study, we could not randomise patients between different categories, and thus could not distinguish total causal effects from direct effects unmediated by other variables on the causal pathway.^49^ Furthermore, our study only assessed mortality within 30 days of sepsis onset, and deaths could have resulted from other causes, suggesting that they were not solely due to sepsis. Another limitation is that the reduction in sepsis diagnosis during the COVID-19 pandemic may have been related to e.g., lower hospital admission rates for patients in nursing homes. As an exploratory analysis, our study mainly showcases associations, reporting odds changes across different subgroups and spotlighting key risk factors warranting heightened attention in sepsis prevention. However, it does not offer precise estimates for the impact of specific risk factors.

In conclusion, patient with higher socioeconomic deprivation and clinical morbidities such as chronic kidney disease, organ transplantation, uncontrolled diabetes, various forms of malignancy, chronic liver disease, neurological diseases, immunosuppressive conditions, and learning disabilities, were associated with development of community-acquired non-COVID-19 sepsis. During the COVID-19 pandemic, the incidence rate of sepsis fluctuated significantly, decreasing initially and then returning to pre-epidemic levels after April 2021. These findings underscore the urgent need for sepsis risk prediction models that account for chronic disease status, deprivation status, and learning disabilities, along with infection severity. This study highlights the need to improve the prevention of sepsis, importance of considering factors commonly associated with health inequalities and the need for more precise targeting of antimicrobials.

## Contributors

Conceptualisation: TvS, DAO, CB, MM, NC, KH, BMK, VP, AP; Methodology: XZ, VP, AP, GM, SB, JM, PI; Formal analysis: XZ, VP, AP; Diagnostic codelists: TvS and OpenSAFELY Collective; Software: JM, PI, LF, OpenSAFELY Collective, BG, BMK; Writing—original draft: XZ; Writing—revising, review and editing: all authors. All read and approved the final manuscript. TvS is the guarantor for the article and accepts full responsibility for the work and/or the conduct of the study, XZ, VP, JM, PI, BM, AM, SB, TvS had access to and verify the underlying data, TvS controlled the decision to publish. The corresponding author attests that all listed authors meet authorship criteria and that no others meeting the criteria have been omitted.

## Data sharing statement

All data were linked, stored and analysed securely within the OpenSAFELY platform https://opensafely.org/. All code is shared openly for review and re-use under MIT open license (https://github.com/opensafely/amr-uom-brit/). Detailed pseudonymised patient data is potentially re-identifiable and therefore not shared.

## Declaration of interests

BG has received research funding from the Laura and John Arnold Foundation, the NHS National Institute for Health Research (NIHR), the NIHR School of Primary Care Research, NHS England, the NIHR Oxford Biomedical Research Centre, the Mohn-Westlake Foundation, NIHR Applied Research Collaboration Oxford and Thames Valley, the Wellcome Trust, the Good Thinking Foundation, Health Data Research UK, the Health Foundation, the World Health Organisation, UKRI MRC, Asthma UK, the British Lung Foundation, and the Longitudinal Health and Wellbeing strand of the National Core Studies programme; he is a Non-Executive Director at NHS Digital; he also receives personal income from speaking and writing for lay audiences on the misuse of science. AM has received consultancy fees (from https://inductionhealthcare.com) and is member of RCGP health informatics group and the NHS Digital GP data Professional Advisory Group that advises on access to GP Data for Pandemic Planning and Research (GDPPR). For the latter, he received payment for the GDPPR role. All other authors declare no competing interests. BMK is a trustee for IMMIGRANT COUNSELLING AND PSYCHOTHERAPY (ICAP), all other declarations can be viewed openly online at https://www.whopaysthisdoctor.org/doctor/491/active.

## Appendix A Supplementary data

Supplementary Materials

List of OpenSAFELY Collaborative
