# Supplementary material for: Clinical and health inequality risk factors for non-COVID-related sepsis during the global COVID-19 pandemic: a national case-control and cohort study
Source: eClinicalMedicine. 2023 Nov 23;66:102321. doi: 10.1016/j.eclinm.2023.102321 (PMC10772239; doi:10.1016/j.eclinm.2023.102321)
Supplement: Supplementary Materials [file mmc1.pdf]

Supplementary materials

Table count: 19
Text count: 6
Figure count: 15

eTable 1. Code lists used for variable definition ... 3
eTable 2. Baseline characteristic for cases with non-COVID-19 sepsis and controls (continued from Table 1 in the main text) ... 4
eTable 3. Baseline demographics and comorbidities for cases in community-acquired/hospital-acquired sepsis ... 5
eTable 4. The total number of cases with new non-COVID-19 sepsis for each specified period ... 6
eTable 5. Discrimination in fully adjusted (all comorbidities*) condition logistic models for differentiating cases with non-COVID-19 and controls ... 6
eTable 6. Percentage of cases with community-acquired non-COVID-19 sepsis dying within 30 days in different COVID-19 period ... 7
eTable 7. Percentage of cases with hospital-acquired non-COVID-19 sepsis dying within 30 days in different COVID-19 period ... 8
eTable 8. Adjusted ORs of community-acquired sepsis 30-day mortality stratified by COVID-19 period ... 9
eTable 9. ORs of developing non-COVID-19 sepsis for factors stratified by sepsis type (Complete case analysis) ... 11
eTable 10. Adjusted ORs of developing non-COVID-19 sepsis for clinical characteristics stratified by sepsis type (Complete case analysis) ... 12
eTable 11. Association between index of multiple deprivations (IMD) quintile and risk of hospital admission for Sepsis (Complete case analysis) ... 13
eTable 12. Adjusted ORs of community-acquired sepsis 30-day mortality stratified by COVID-19 period (Complete risk analysis) ... 14
eTable 13. Adjusted ORs of community-acquired sepsis 30-day mortality (COVID-19 period as a moderator) ... 16
eTable 14. The underlying number of Figure 2 ... 18
eTable 15. ORs of developing non-COVID-19 sepsis for factors stratified by sepsis type (Multiple imputation) ... 23
eTable 16. ORs of developing non-COVID-19 sepsis for factors stratified by sepsis type (Multiple imputation, 18+ years old study population) ... 24
eTable 17. Adjusted ORs of developing non-COVID-19 sepsis for clinical characteristics stratified by sepsis type (Multiple imputation) ... 25
eTable 18. Adjusted ORs of developing non-COVID-19 sepsis for clinical characteristics stratified by sepsis type (Multiple imputation, 18+ years old study population) ... 26
eTable 19. Adjusted ORs of community-acquired sepsis 30-day mortality stratified by COVID-19 period (Multiple imputation) ... 27

eText 1. The selection criteria for potential controls ... 29
eText 2. The definition of Index of Multiple Deprivation (IMD) quintile ... 29
eText 3. The definition of predictors ... 29
eText 4. Missing data ... 29
eText 5. Model adjustments for IMD ... 30
eText 6. Comparing the different approaches to dealing with missing data ... 30

eFigure 1. Diagram of patient selection (index date) and prior antibiotic measurements ... 31
eFigure 2. Age-specific incidence rate of community-acquired sepsis ... 32
eFigure 3. Age-specific incidence rate of hospital-acquired sepsis ... 33
eFigure 4. Association between index of multiple deprivations (IMD) quintile and risk of hospital admission for Sepsis 34
eFigure 5. ORs for the number of antibiotics given within six weeks before the index date ... 35
eFigure 6. Association between index of multiple deprivations (IMD) quintile and risk of hospital admission for Sepsis (18+ years old study population) ... 36
eFigure 7. Crude ORs of sepsis by region, ethnicity, BMI, smoking history and stratified by type of sepsis (18+ years old study population) ... 37
eFigure 8. Crude ORs of developing sepsis for clinical characteristics stratified by sepsis type (18+ years old study population) ... 39
eFigure 9. Adjusted ORs of developing sepsis for clinical characteristics stratified by sepsis type (18+ years old study population) ... 41
eFigure 10. Adjusted ORs of all sepsis mortality stratified by COVID-19 period ... 43
eFigure 11. Adjusted ORs of hospital-acquired sepsis mortality stratified by COVID-19 period ... 45
eFigure 12. Fully adjusted ORs of all sepsis mortality stratified by COVID-19 period ... 47

eFigure 13. Fully adjusted ORs of community-acquired sepsis mortality stratified by COVID-19 period 49
eFigure 14. Fully adjusted ORs of hospital-acquired sepsis mortality stratified by COVID-19 period.............................. 51
eFigure 15. Directed acyclic graph (DAG) of the association between IMD (socioeconomic status) and the risk of developing non-COVID-19 sepsis 52

Supplement: the OpenSAFELY collaborative ………………………………………………………………………… 54

eTable 1. Code lists used for variable definition

1. Code lists are available at https://www.opencodelists.org/
2. Link: https://www.opencodelists.org/codelist/ add code list version

variable	Codelist version
Ethnicity	https://codelists.opensafely.org/codelist/opensafely/ethnicity/2020-04-27/
Sepsis (hospital)	https://codelists.opensafely.org/codelist/user/BillyZhongUOM/codes_for_sepsis/00ab8f66/
Sepsis (primary care)	https://codelists.opensafely.org/codelist/user/rriefu/sepsis/7224264f/
**Covid diagnosis**	
Covid<br>(Primary care)	https://codelists.opensafely.org/codelist/opensafely/covid-identification-in-primary-care-probable-covid-clinical-code/24391856
Covid positive test<br>(Primary care)	https://codelists.opensafely.org/codelist/opensafely/covid-identification-in-primary-care-probable-covid-positive-test/3d488b8b/
Covid (primary care sequalae)	https://codelists.opensafely.org/codelist/opensafely/covid-identification-in-primary-care-probable-covid-sequelae/0b29a521/
covid_codelist	codelist(["U071", "U072"], system="icd10")
confirmed_covid_codelist	codelist(["U071"], system="icd10")
suspected_covid_codelist	codelist(["U072"], system="icd10")
Smoking	"https://codelists.opensafely.org/codelist/opensafely/smoking-clear/2020-04-29/"
Hazardous alcohol use	"https://codelists.opensafely.org/codelist/opensafely/hazardous-alcohol-drinking/6364474b/"
**Comorbidities**	
Hypertension diagnosis	https://codelists.opensafely.org/codelist/opensafely/hypertension/2020-04-28/
Chronic respiratory disease diagnosis	https://codelists.opensafely.org/codelist/opensafely/chronic-respiratory-disease/2020-04-10/
Asthma diagnosis	"https://codelists.opensafely.org/codelist/opensafely/asthma-diagnosis/2020-04-15/"<br>https://codelists.opensafely.org/codelist/opensafely/asthma-oral-prednisolone-medication/2020-04-27/
Blood pressure	Systolic ["2469."], system="ctv3",)<br>Diastolic ["246A."],system="ctv3")
Chronic cardiac disease diagnosis	https://codelists.opensafely.org/codelist/opensafely/chronic-cardiac-disease/2020-04-08/
Diabetes diagnosis	https://codelists.opensafely.org/codelist/opensafely/diabetes/2020-04-15/
Measures of hba1c	https://codelists.opensafely.org/codelist/opensafely/glycated-haemoglobin-hba1c-tests-ifcc/6d7287f8/
Cancer diagnosis	https://codelists.opensafely.org/codelist/opensafely/haematological-cancer/2020-04-15/<br>https://codelists.opensafely.org/codelist/opensafely/lung-cancer/2020-04-15/<br>"https://codelists.opensafely.org/codelist/opensafely/cancer-excluding-lung-and-haematological/2020-04-15/"
Dialysis	https://codelists.opensafely.org/codelist/opensafely/dialysis/3ce108ac/
Kidney transplant	https://codelists.opensafely.org/codelist/opensafely/kidney-transplant/2020-07-15/
Creatinine codes	codelist(["XE2q5"], system="ctv3")
Chronic liver disease diagnosis	https://codelists.opensafely.org/codelist/opensafely/chronic-liver-disease/2020-06-02/
Stroke	https://codelists.opensafely.org/codelist/opensafely/stroke-updated/2020-06-02/
Dementia diagnosis	https://codelists.opensafely.org/codelist/opensafely/dementia/2020-04-22/
Other neurolgoical conditions	https://codelists.opensafely.org/codelist/opensafely/other-neurological-conditions/2020-06-02/"
Presence of organ transplant (excluding kidney transplants)	https://codelists.opensafely.org/codelist/opensafely/other-organ-transplant/79caeeee/
Asplenia or dysplenia (acquired or congenital) diagnosis	https://codelists.opensafely.org/codelist/opensafely/asplenia/2020-06-02/
Sickle cell disease diagnosis	https://codelists.opensafely.org/codelist/opensafely/sickle-cell-disease/2020-04-14/
Rheumatoid/Lupus/Psoriasis diagnosis	https://codelists.opensafely.org/codelist/opensafely/ra-sle-psoriasis/2020-04-14/
Immunosuppressive condition	https://codelists.opensafely.org/codelist/primis-covid19-vacc-uptake/immdx_cov/v1/<br>https://codelists.opensafely.org/codelist/primis-covid19-vacc-uptake/immrx/v1/
Learning disabilities	https://codelists.opensafely.org/codelist/nhsd-primary-care-domain-refsets/ld_cod/20210127/
Severe mental illness	https://codelists.opensafely.org/codelist/primis-covid19-vacc-uptake/sev_mental/v1/
Antibacterial	https://codelists.opensafely.org/codelist/user/BillyZhongUOM/brit_new_dmd/792101bd

eTable 2. Baseline characteristic for cases with non-COVID-19 sepsis and controls (continued from Table 1 in the main text)

Variables[a,b]		Case		Control	
		Number of cases n(%)		Number of controls n(%)	
**Alcohol problems**	No	198540	88.5	1245100	92.5
	Yes	25820	11.5	101065	7.5
**Carehome status**	No	210170	94.0	1314195	97.8
	Yes	13505	6.0	30020	2.2
**Learning disability**	No	221225	98.6	1342300	99.7
	Yes	3135	1.4	3865	0.3
**Asthma**	No asthma	188610	84.1	1166400	86.6
	With no oral steroid use	29125	13.0	156495	11.6
	With oral steroid use	6625	3.0	23275	1.7
**Diabetes**	No diabetes	152555	68.0	1077195	80.0
	Controlled	60035	26.8	230035	17.1
	Not controlled	3820	1.7	8725	0.6
	Without recent Hb1ac measure	7950	3.5	30215	2.2
**Organ transplant**	No transplant	221865	98.9	1344825	99.9
	Kidney transplant	1950	0.9	950	0.1
	Other organ transplant	545	0.2	390	0.0
**Chronic kidney disease or renal replacement therapy**	No CKD or RRT	152590	68.0	1062410	78.9
	CKD stage 3a	32315	14.4	172860	12.8
	CKD stage 3b	22825	10.2	84390	6.3
	CKD stage 4	10185	4.5	21980	1.6
	CKD stage 5	2140	1.0	1920	0.1
	RRT (dialysis)	2800	1.2	1805	0.1
	RRT (transplant)	1505	0.7	800	0.1
**High blood pressure or diagnosed hypertension**	No	100255	44.7	708230	52.6
	Yes	124110	55.3	637935	47.4
**Chronic respiratroy disease**	No	183900	82.0	1219635	90.6
	Yes	40460	18.0	126530	9.4
**Chronic cardiac disease**	No	157070	70.0	1088355	80.8
	Yes	67290	30.0	257815	19.2
**Cancer (non haematological)**	No	168480	75.1	1179105	87.6
	Yes	55880	24.9	167060	12.4
**Haematological malignancy**	No	210235	93.7	1329150	98.7
	Yes	14125	6.3	17020	1.3
**Chronic liver disease**	No	217025	96.7	1336550	99.3
	Yes	7335	3.3	9620	0.7
**Stroke**	No	197065	87.8	1256620	93.3
	Yes	27295	12.2	89545	6.7
**Dementia**	No	218735	97.5	1331000	98.9
	Yes	5625	2.5	15165	1.1
**Other neurological disease**	No	213100	95.0	1322375	98.2
	Yes	11260	5.0	23795	1.8
**Asplenia**	No	222865	99.3	1343155	99.8
	Yes	1495	0.7	3010	0.2
**Rheumatoid arthritis/lupus/psoriasis**	No	203735	90.8	1258265	93.5
	Yes	20625	9.2	87900	6.5
**Immunosuppressive condition**	No	203895	90.9	1315285	97.7
	Yes	20465	9.1	30880	2.3
**Severe mental illness**	No	218210	97.3	1331465	98.9
	Yes	6150	2.7	14700	1.1

To reduce the risk of secondary disclosure, all counted numbers in the baseline table were rounded to the nearest five

a. The codelist of the variable definition was listed in eTable 1.
b. The definition of the variables was described in eText 3

eTable 3. Baseline demographics and comorbidities for cases in community-acquired/hospital-acquired sepsis

		Community-acquired cases		Hospital-acquired cases	
**Age**	Mean(SD)	70.9	19.0	64.4	19.6
		**Number of cases n(%)**		**Number of cases n(%)**	
**Sex**	Female	87400	48.6	21536	48.5
**Region**	North East	8195	4.6	2080	4.7
	North West	16795	9.3	4285	9.7
	Yorkshire and the Humber	24905	13.8	6025	13.6
	East Midlands	39660	22.0	9445	21.3
	West Midlands	7380	4.1	1665	3.7
	East of England	41845	23.2	10235	23.1
	London	8260	4.6	2275	5.1
	South East	12955	7.2	2695	6.1
	South West	20005	11.1	5660	12.8
**IMD[a] quintile**	5 (least deprived)	29665	16.5	8070	18.2
	4	35200	19.6	9120	20.6
	3	39080	21.7	9620	21.7
	2	37230	20.7	8805	19.8
	1 (most deprived)	38825	21.6	8750	19.7
**Ethnicity[b]**	White	165475	91.9	40425	91.1
	Mixed	1095	0.6	335	0.8
	South Asian	7540	4.2	2180	4.9
	Black	2355	1.3	620	1.4
	Other	1585	0.9	515	1.2
	Unknown	1940	1.1	295	0.7
**BMI[c]**	Healthy range (18.5-24.9kg/m2)	51030	28.4	12880	29
	Underweight (<18.5 kg/m2)	9415	5.2	1820	4.1
	Overweight (25-29.9 kg/m2)	45035	25	11985	27
	Obese I (30-34.9 kg/m2)	26050	14.5	6580	14.8
	Obese II (35-39.9 kg/m2)	11705	6.5	2860	6.4
	Obese III (40+ kg/m2)	9665	5.4	1740	3.9
	Unknown	27090	15.1	6500	14.6
**Smoking status[d]**	Never	59215	32.9	15150	34.1
	Former	91650	50.9	21905	49.4
	Current	25055	13.9	6020	13.6
	unknown	4075	2.3	1295	2.9

To reduce the risk of secondary disclosure, all counted numbers in the baseline table were rounded to the nearest five
a. IMD (Index of Multiple Deprivation) quintile measured from patient-level address
b. Ethnicity in line with 2001 Census categories
c. BMI, Body Mass Index groups based on the NICE definitions
d. Smoking status identified from the most recent clinical records

eTable 4. The total number of cases with new non-COVID-19 sepsis for each specified period

		Period 1	Period 2	Period 3
Community-acquired	Male	40120	19570	30500
	Female	42525	18555	28720
Hospital-acquired	Male	10915	4580	7335
	Female	10430	4215	6890

To reduce the risk of secondary disclosure, all counted numbers in the baseline table were rounded to the nearest five
Period 1: 2019-01-01 to 2020-03-25; Period 2: 2020-03-26 to 2021-03-08; Period 3: 2021-03-09 to 2022-06-30

eTable 5. Discrimination in fully adjusted (all comorbidities*) condition logistic models for differentiating cases with non-COVID-19 and controls

	C-statistic
All	0.769
Community-acquired	0.753
Hospital-acquired	0.857
All (18+ only)	0.769
Community-acquired (18+ only)	0.754
Hospital-acquired (18+ only)	0.855

All comorbidities* Hypertension, chronic cardiac disease, diabetes, stroke, chronic kidney disease or renal replacement therapy) and asthma, cancer (non-haematological and haematological), chronic liver disease, dementia, other neurological disease (including motor neuron disease, myasthenia gravis, multiple sclerosis, Parkinson's disease, cerebral palsy, quadriplegia or hemiplegia, and progressive cerebellar disease), organ kidney transplant, asplenia (due to splenectomy or spleen dysfunction, including sickle cell disease), rheumatoid arthritis/lupus/psoriasis, other immunosuppressive conditions, learning disability, several mental ill, the number of prior antibiotic prescription from one year and six week to six week before the index time (indicating the infection history)

eTable 6. Percentage of cases with community-acquired non-COVID-19 sepsis dying within 30 days in different COVID-19 period

**Variables**		**Period 1**	**Period 2**	**Period 3**
Number of deaths	N	16560	9365	14470
Age	<18	2.1%	2.2%	1.8%
	18-39	2.9%	3.9%	3.3%
	40-49	8.4%	12.9%	12.7%
	50-59	11.8%	17.3%	16.8%
	60-69	15.4%	20.5%	19.6%
	70-79	19.6%	24.1%	24.2%
	80+	28.8%	33.7%	33.8%
Sex	Female	20.3%	24.7%	24.7%
	Male	19.8%	24.5%	24.2%
Region	East of England	20.6%	23.4%	24.9%
	East Midlands	19.6%	25.4%	24.4%
	London	16.0%	20.9%	19.8%
	North East	20.0%	24.2%	24.5%
	North West	22.2%	26.7%	25.0%
	South East	16.8%	21.5%	21.2%
	South West	19.9%	24.0%	24.0%
	West Midlands	20.4%	26.5%	24.9%
	Yorkshire and The Humber	21.3%	26.6%	26.7%
IMD	1 (Most deprived)	19.7%	24.2%	24.1%
	2	20.7%	24.9%	24.0%
	3	20.0%	24.6%	24.4%
	4	20.1%	24.6%	24.8%
	5 (Least deprived)	19.6%	24.5%	24.9%
Ethnicity	Black	11.8%	16.7%	13.4%
	Mixed	8.6%	15.9%	12.2%
	Other	13.7%	20.3%	18.4%
	South Asian	12.9%	17.7%	16.4%
	Unknown	32.8%	31.8%	35.0%
	White	20.5%	24.9%	25.0%

To reduce the risk of secondary disclosure, all counted numbers in the baseline table were rounded to the nearest five
Period 1: 2019-01-01 to 2020-03-25; Period 2: 2020-03-26 to 2021-03-08; Period 3: 2021-03-09 to 2022-06-30
IMD (Index of Multiple Deprivation) quintile measured from patient-level address
BMI, Body Mass Index groups based on the NICE definitions

eTable 7. Percentage of cases with hospital-acquired non-COVID-19 sepsis dying within 30 days in different COVID-19 period

**Variables**		**Period 1**	**Period 2**	**Period 3**
Number of deaths	N	3480	1685	2750
**Age**	<18	2.6%	2.0%	2.6%
	18-39	2.1%	2.1%	2.3%
	40-49	7.1%	9.3%	11.8%
	50-59	10.8%	12.8%	13.2%
	60-69	14.5%	18.3%	18.2%
	70-79	18.6%	23.0%	22.7%
	80+	28.9%	33.1%	32.2%
**Sex**	Female	14.4%	17.3%	18.1%
	Male	18.1%	20.9%	20.5%
**Region**	East of England	16.2%	18.7%	20.3%
	East Midlands	15.5%	20.0%	17.5%
	London	14.7%	10.1%	16.1%
	North East	17.4%	16.5%	20.6%
	North West	16.3%	22.0%	21.8%
	South East	17.1%	21.1%	17.0%
	South West	15.5%	18.8%	19.5%
	West Midlands	18.9%	22.4%	21.6%
	Yorkshire and The Humber	17.4%	19.8%	20.2%
**IMD**	1 (Most deprived)	15.3%	18.1%	19.0%
	2	16.5%	19.1%	19.5%
	3	16.4%	20.1%	19.7%
	4	16.7%	18.6%	19.6%
	5 (Least deprived)	16.7%	19.6%	18.9%
**Ethnicity**	Black	14.5%	12.5%	11.1%
	Mixed	12.9%	0.0%	8.7%
	Other	8.3%	4.8%	14.7%
	South Asian	8.6%	9.8%	10.3%
	Unknown	25.8%	27.3%	29.4%
	White	16.8%	19.9%	20.1%

To reduce the risk of secondary disclosure, all counted numbers in the baseline table were rounded to the nearest five
Period 1: 2019-01-01 to 2020-03-25; Period 2: 2020-03-26 to 2021-03-08; Period 3: 2021-03-09 to 2022-06-30
IMD (Index of Multiple Deprivation) quintile measured from patient-level address
BMI, Body Mass Index groups based on the NICE definitions

eTable 8. Adjusted ORs of community-acquired sepsis 30-day mortality stratified by COVID-19 period

Group	Category	OR (Community + Hospital)	OR2 (Community)	OR3 (Hospital)
Age	18-39	0.19(0.16,0.23)	0.19(0.15,0.23)	0.17(0.14,0.20)
Age	40-49	0.59(0.51,0.68)	0.71(0.60,0.83)	0.71(0.62,0.82)
Age	60-69	1.36(1.24,1.48)	1.23(1.10,1.37)	1.21(1.10,1.33)
Age	70-79	1.81(1.67,1.96)	1.52(1.37,1.68)	1.59(1.46,1.72)
Age	80+	3.01(2.79,3.25)	2.44(2.22,2.69)	2.53(2.34,2.74)
Age	<18	0.15(0.11,0.22)	0.10(0.05,0.16)	0.09(0.05,0.13)
Antibiotic count	Antibiotic count: 1	0.97(0.92,1.02)	0.95(0.89,1.02)	0.98(0.93,1.04)
Antibiotic count	Antibiotic count: 2-3	1.00(0.95,1.05)	1.07(1.00,1.14)	1.02(0.97,1.08)
Antibiotic count	Antibiotic count: 3+	1.01(0.97,1.06)	1.05(0.98,1.12)	1.05(0.99,1.10)
Asthma	With no oral steroid use	0.84(0.79,0.89)	0.88(0.81,0.95)	0.87(0.82,0.92)
Asthma	With oral steroid use	0.85(0.77,0.95)	1.09(0.94,1.25)	0.97(0.86,1.09)
BMI	Obese I (30-34.9 kg/m2)	0.83(0.78,0.88)	0.83(0.76,0.90)	0.83(0.78,0.89)
BMI	Obese II (35-39.9 kg/m2)	0.85(0.78,0.92)	0.81(0.72,0.90)	0.89(0.81,0.97)
BMI	Obese III (40+ kg/m2)	0.92(0.83,1.01)	1.04(0.92,1.17)	0.99(0.90,1.09)
BMI	Overweight (25-29.9 kg/m2)	0.92(0.87,0.96)	0.89(0.84,0.96)	0.83(0.79,0.88)
BMI	Underweight (<18.5 kg/m2)	1.10(1.02,1.19)	1.12(1.00,1.25)	1.16(1.06,1.27)
CKD/RRT	CKD stage 3a	0.95(0.91,1.00)	1.02(0.95,1.09)	0.90(0.86,0.96)
CKD/RRT	CKD stage 3b	1.13(1.07,1.19)	1.25(1.16,1.35)	1.12(1.06,1.19)
CKD/RRT	CKD stage 4	1.54(1.43,1.65)	1.59(1.44,1.75)	1.41(1.30,1.53)
CKD/RRT	CKD stage 5	1.67(1.42,1.96)	1.79(1.44,2.22)	1.37(1.15,1.63)
CKD/RRT	RRT (dialysis)	1.24(1.05,1.46)	1.02(0.80,1.30)	0.82(0.65,1.01)
CKD/RRT	RRT (transplant)	0.54(0.39,0.73)	0.58(0.37,0.86)	0.65(0.46,0.89)
Diabetes	Controlled	0.93(0.90,0.97)	0.93(0.88,0.98)	0.93(0.89,0.98)
Diabetes	Not controlled	0.86(0.75,0.98)	0.84(0.69,1.02)	0.81(0.69,0.94)
Diabetes	Without recent Hb1ac measure	0.99(0.89,1.1)	0.99(0.88,1.12)	1.04(0.953,1.14)
Ethnicity	Black	0.73(0.60,0.89)	0.89(0.69,1.13)	0.64(0.51,0.79)
Ethnicity	Mixed	0.61(0.43,0.84)	1.05(0.71,1.52)	0.73(0.53,1.00)
Ethnicity	Other	0.88(0.70,1.09)	1.11(0.83,1.47)	1.06(0.83,1.34)
Ethnicity	South Asian	0.81(0.73,0.90)	0.94(0.80,1.09)	0.86(0.77,0.96)
IMD	IMD1(most deprived)	1.25(1.18,1.32)	1.21(1.12,1.32)	1.21(1.14,1.29)
IMD	IMD2	1.21(1.14,1.29)	1.16(1.07,1.25)	1.08(1.01,1.15)
IMD	IMD3	1.09(1.03,1.16)	1.07(0.99,1.16)	1.04(0.98,1.11)
IMD	IMD4	1.06(1.00,1.13)	1.03(0.95,1.12)	1.02(0.96,1.09)
Others	Alcohol problems	1.35(1.27,1.43)	1.46(1.35,1.57)	1.35(1.27,1.43)
Others	Asplenia	1.36(1.08,1.70)	1.27(0.94,1.70)	0.83(0.62,1.08)
Others	Cancer (non haematological)	1.29(1.24,1.35)	1.26(1.2,1.34)	1.26(1.20,1.31)
Others	Chronic cardiac disease	1.13(1.09,1.18)	1.14(1.08,1.20)	1.14(1.09,1.18)
Others	Chronic liver disease	1.84(1.67,2.02)	2.05(1.81,2.32)	2.04(1.84,2.25)
Others	Chronic respiratory disease	1.19(1.14,1.24)	1.29(1.21,1.37)	1.33(1.27,1.40)
Others	Dementia	1.08(0.99,1.18)	1.14(1.00,1.31)	1.15(1.03,1.29)
Others	Haematological malignancy	0.97(0.88,1.06)	0.94(0.83,1.06)	0.88(0.80,0.98)
Others	Hypertension	0.97(0.93,1.01)	0.98(0.93,1.04)	0.94(0.90,0.98)

Others	Immunosuppressive condition	1.03(0.96,1.11)	0.99(0.90,1.10)	0.98(0.90,1.06)
Others	Learning disability	1.38(1.17,1.61)	1.35(1.07,1.68)	0.98(0.80,1.18)
Others	Other neurological disease	0.99(0.92,1.07)	1.00(0.90,1.11)	1.01(0.93,1.11)
Others	Potential Care Home	0.80(0.76,0.85)	1.21(1.08,1.36)	1.28(1.15,1.41)
Others	Rheumatoid arthritis/ lupus/ psoriasis	1.00(0.94,1.06)	1.02(0.94,1.11)	1.04(0.98,1.11)
Others	Severe mental illness	0.90(0.81,1.01)	1.00(0.86,1.16)	0.92(0.81,1.04)
Others	Stroke	1.03(0.98,1.09)	0.99(0.93,1.06)	0.97(0.92,1.03)
Region	East Midlands	0.95(0.91,1.01)	1.14(1.06,1.22)	1.00(0.94,1.05)
Region	London	0.80(0.72,0.88)	0.98(0.86,1.11)	0.84(0.76,0.94)
Region	North East	1.04(0.95,1.14)	1.15(1.01,1.30)	1.06(0.96,1.17)
Region	North West	1.18(1.10,1.26)	1.27(1.16,1.39)	1.09(1.01,1.17)
Region	South East	0.76(0.70,0.82)	0.86(0.78,0.96)	0.79(0.72,0.86)
Region	South West	0.98(0.92,1.05)	1.04(0.95,1.13)	0.96(0.90,1.03)
Region	West Midlands	1.10(0.99,1.21)	1.37(1.19,1.57)	1.15(1.04,1.26)
Region	Yorkshire and the Humber	1.08(1.02,1.15)	1.23(1.13,1.33)	1.17(1.10,1.25)
Sex	Male	0.97(0.94,1.01)	0.97(0.92,1.02)	0.96(0.92,1.00)
Smoking	Current	1.58(1.49,1.68)	1.61(1.49,1.75)	1.68(1.57,1.79)
Smoking	Former	1.07(1.03,1.11)	1.1(1.04,1.16)	1.15(1.10,1.20)
Tx	Kidney transplant	0.62(0.47,0.79)	0.54(0.37,0.77)	0.59(0.43,0.79)
Tx	Other organ transplant	0.95(0.61,1.43)	0.46(0.19,0.95)	0.84(0.49,1.37)

Models were adjusted for age using a 4-knot cubic spline, except for estimation of age group relative odds of 30-day mortality; and adjusted for sex, except for estimation of sex group relative odds of 30-day mortality; and stratified by region, except for IMD group relative odds of 30-day mortality.
Abbreviations: IMD, index of multiple deprivation; BMI, body mass index;
Tx, transplant; CKD, chronic kidney disease; RRT, renal replacement therapy.

The number of prior antibiotic prescriptions (one year plus six weeks to six weeks before the index date)

Reference sub-group:
age: 50-59 years
sex: female
region: East of England
IMD: the least deprived quintile
ethnicity: white
BMI: healthy range (18.5 – 24.9 kg/m2)
smoking: never
prior antibiotic count: 0
Patients without the disease were used as the reference for other clinical conditions.

eTable 9. ORs of developing non-COVID-19 sepsis for factors stratified by sepsis type (Complete case analysis)

	OR (Community + Hospital)	OR2 (Community)	OR3 (Hospital)
IMD			
IMD 1 (Most deprived)	1.79 (1.76-1.82)	1.88 (1.85-1.92)	1.45 (1.39-1.50)
IMD 2	1.48 (1.45-1.50)	1.53 (1.50-1.56)	1.29 (1.24-1.34)
IMD 3	1.24 (1.22-1.26)	1.27 (1.25-1.29)	1.14 (1.10-1.18)
IMD 4	1.14 (1.12-1.16)	1.15 (1.13-1.17)	1.12 (1.08-1.16)
Ethnicity			
Mixed	0.95 (0.90-1.01)	0.96 (0.90-1.02)	0.97 (0.86-1.09)
South Asian	1.08 (1.05-1.11)	1.08 (1.05-1.11)	1.07 (1.02-1.12)
Black	0.96 (0.92-1.00)	0.96 (0.92-1.01)	0.89 (0.82-0.98)
Other	0.80 (0.76-0.85)	0.78 (0.74-0.83)	0.86 (0.77-0.94)
BMI			
Underweight (<18.5 kg/m2)	1.72 (1.67-1.75)	1.73 (1.68-1.79)	1.67 (1.58-1.77)
Overweight (25-29.9 kg/m2)	0.86 (0.85-0.87)	0.86 (0.85-0.87)	0.84 (0.81-0.86)
Obese I (30-34.9 kg/m2)	1.04 (1.02-1.05)	1.07 (1.05-1.10)	0.92 (0.89-0.95)
Obese II (35-39.9 kg/m2)	1.39 (1.36-1.41)	1.46 (1.43-1.50)	1.12 (1.07-1.17)
Obese III (40+ kg/m2)	2.23 (2.17-2.28)	2.54 (2.48-2.61)	1.29 (1.22-1.37)
Smoking			
Former	1.34 (1.33-1.36)	1.34 (1.34-1.36)	1.35 (1.32-1.38)
Current	1.80 (1.78-1.84)	1.92 (1.89-1.96)	1.46 (1.42-1.51)

Crude ORs of sepsis by IMD quintile, ethnicity, BMI, smoking history and stratified by type of sepsis

Reference groups:
IMD quintile: the least deprived quintile (IMD 5)
Ethnicity: white
BMI: healthy range (18.5 – 24.9 kg/m2)
Smoking: never (Smoking status identified from the most recent clinical records)

Abbreviations:
IMD (Index of Multiple Deprivation) quintile measured from patient-level address
BMI, Body Mass Index (from the most recent clinical records)

eTable 10. Adjusted ORs of developing non-COVID-19 sepsis for clinical characteristics stratified by sepsis type (Complete case analysis)

type	OR (Community + Hospital)	OR2 (Community)	OR3 (Hospital)
Potential Care Home	2.95 (2.86-3.05)	2.94 (2.85-3.04)	2.87 (2.6-3.17)
CKD stage 3a	1.23 (1.2-1.26)	1.23 (1.2-1.27)	1.19 (1.12-1.27)
CKD stage 3b	1.67 (1.62-1.71)	1.66 (1.61-1.71)	1.76 (1.63-1.9)
CKD stage 4	2.53 (2.43-2.64)	2.45 (2.34-2.56)	3.22 (2.86-3.63)
CKD stage 5	6.01 (5.41-6.67)	5.45 (4.86-6.1)	12.3 (9.38-16.15)
RRT (dialysis)	7.52 (6.79-8.34)	6.43 (5.73-7.22)	18.66 (14.56-23.92)
RRT (transplant)	6.72 (4.78-9.44)	6.1 (4.15-8.99)	12.59 (5.92-26.74)
With no oral steroid use	1.01 (0.99-1.03)	1.02 (0.99-1.04)	1.02 (0.97-1.08)
With oral steroid use	0.86 (0.83-0.9)	0.87 (0.83-0.91)	0.86 (0.77-0.96)
Controlled	1.46 (1.44-1.49)	1.5 (1.47-1.53)	1.31 (1.25-1.38)
Not controlled	2.54 (2.38-2.7)	2.69 (2.51-2.88)	1.92 (1.61-2.28)
Without recent Hb1ac measure	1.67 (1.59-1.75)	1.72 (1.63-1.81)	1.41 (1.24-1.6)
Kidney transplant	1.43 (1.05-1.96)	1.61 (1.13-2.3)	0.84 (0.43-1.65)
Other organ transplant	2.06 (1.65-2.56)	2.03 (1.58-2.6)	2.53 (1.53-4.17)
Hypertension	1.1 (1.08-1.12)	1.11 (1.09-1.13)	1.07 (1.02-1.11)
Chronic respiratory disease	1.49 (1.46-1.52)	1.5 (1.47-1.54)	1.46 (1.38-1.54)
Chronic cardiac disease	1.39 (1.36-1.41)	1.37 (1.34-1.4)	1.55 (1.48-1.62)
Cancer (non haematological)	2.38 (2.34-2.42)	1.72 (1.69-1.76)	7.71 (7.4-8.04)
Haematological malignancy	2.57 (2.42-2.73)	1.83 (1.7-1.96)	4.72 (4.15-5.36)
Chronic liver disease	2.98 (2.82-3.14)	2.97 (2.8-3.15)	3.35 (2.93-3.84)
Stroke	1.48 (1.45-1.52)	1.49 (1.45-1.53)	1.44 (1.34-1.55)
Dementia	1.34 (1.28-1.41)	1.35 (1.28-1.43)	1.24 (1.05-1.46)
Other neurological disease	2.30 (2.22-2.39)	2.41 (2.31-2.51)	1.94 (1.75-2.16)
Asplenia	1.05 (0.94-1.16)	1.05 (0.93-1.19)	1.33 (1.04-1.71)
Rheumatoid arthritis/ lupus/ psoriasis	1.28 (1.24-1.31)	1.3 (1.27-1.34)	1.2 (1.12-1.28)
Immunosuppressive condition	2.16 (2.06-2.27)	1.67 (1.57-1.77)	5.27 (4.73-5.86)
Learning disability	3.66 (3.38-3.95)	4 (3.67-4.37)	2.7 (2.21-3.31)
Severe mental illness	1.92 (1.83-2.02)	2.06 (1.95-2.17)	1.4 (1.22-1.6)
Alcohol problems	1.39 (1.35-1.42)	1.42 (1.38-1.46)	1.26 (1.18-1.34)
Antibiotic count: 1	1.68 (1.65-1.71)	1.65 (1.62-1.69)	1.76 (1.68-1.84)
Antibiotic count: 2-3	2.26 (2.22-2.31)	2.24 (2.19-2.29)	2.34 (2.22-2.47)
Antibiotic count: 3+	3.45 (3.37-3.52)	3.43 (3.35-3.52)	3.57 (3.37-3.79)

Reference groups:
Clinical characteristics: the patients without the clinical disease.
The number of prior antibiotic prescriptions: antibiotic count: 0.
Models were adjusted for all comorbidities*

All comorbidities* Hypertension, chronic cardiac disease, diabetes, stroke, chronic kidney disease or renal replacement therapy) and asthma, cancer (non-haematological and haematological), chronic liver disease, dementia, other neurological disease (including motor neuron disease, myasthenia gravis, multiple sclerosis, Parkinson's disease, cerebral palsy, quadriplegia or hemiplegia, and progressive cerebellar disease), organ kidney transplant, asplenia (due to splenectomy or spleen dysfunction, including sickle cell disease), rheumatoid arthritis/lupus/psoriasis, other immunosuppressive conditions, learning disability, several mental ill, the number of prior antibiotic prescription from one year and six week to six week before the index time (indicating the infection history)

Abbreviations:
CKD, chronic kidney disease; RRT, renal replacement therapy.
The number of prior antibiotic prescriptions (one year plus six weeks to six weeks before the index date), The ORs for the number of antibiotics given within six weeks before the index date can be found in Supplementary eFigure 5.

eTable 11. Association between index of multiple deprivations (IMD) quintile and risk of hospital admission for Sepsis (Complete case analysis)

	OR (Community + Hospital)	OR2 (Community)	OR3 (Hospital)
IMD 1 (Most deprived)			
Unadjusted	1.79 (1.76-1.82)	1.88 (1.85-1.92)	1.45 (1.39-1.50)
Model 1	1.75 (1.71-1.80)	1.83 (1.79-1.88)	1.48 (1.41-1.56)
Model 2	1.59 (1.56-1.62)	1.67 (1.64-1.71)	1.31 (1.26-1.36)
Model 3 (fully adjusted)	1.38 (1.36-1.40)	1.42 (1.40-1.44)	1.25 (1.21-1.30)
IMD 2			
Unadjusted	1.48 (1.45-1.50)	1.53 (1.50-1.56)	1.29 (1.24-1.34)
Model 1	1.46 (1.42-1.49)	1.50 (1.46-1.54)	1.30 (1.24-1.37)
Model 2	1.36 (1.34-1.39)	1.41 (1.38-1.44)	1.20 (1.16-1.25)
Model 3 (fully adjusted)	1.26 (1.23-1.28)	1.28 (1.25-1.30)	1.16 (1.12-1.21)
IMD 3			
Unadjusted	1.24 (1.22-1.26)	1.27 (1.25-1.29)	1.14 (1.10-1.18)
Model 1	1.22 (1.19-1.25)	1.24 (1.21-1.28)	1.15 (1.09-1.21)
Model 2	1.18 (1.16-1.20)	1.21 (1.19-1.23)	1.09 (1.05-1.13)
Model 3 (fully adjusted)	1.12 (1.11-1.14)	1.14 (1.12-1.16)	1.09 (1.05-1.13)
IMD 4			
Unadjusted	1.14 (1.12-1.16)	1.15 (1.13-1.17)	1.12 (1.08-1.16)
Model 1	1.13 (1.10-1.16)	1.13 (1.10-1.17)	1.12 (1.06-1.18)
Model 2	1.11 (1.10-1.13)	1.12 (1.10-1.14)	1.09 (1.05-1.14)
Model 3 (fully adjusted)	1.09 (1.07-1.10)	1.09 (1.07-1.11)	1.08 (1.04-1.11)

IMD (Index of Multiple Deprivation) quintile measured from patient-level address.
Odds ratios are compared to the least deprived quintile (IMD 5)
Model 1 – adjusted for interaction term between IMD and time (Period 1: 2019-01-01 to 2020-03-25; Period 2: 2020-03-26 to 2021-03-08; Period 3: 2021-03-09 to 2022-06-30)
Model 2 – adjusted for cardiometabolic comorbidities*
Model 3 (fully adjusted model) – adjust for all comorbidities**

Cardiometabolic comorbidities* Hypertension, chronic cardiac disease, diabetes, stroke, chronic kidney disease or renal replacement therapy)
All comorbidities** Cardiometabolic comorbidities and asthma, cancer (non-haematological and haematological), chronic liver disease, dementia, other neurological disease (including motor neuron disease, myasthenia gravis, multiple sclerosis, Parkinson's disease, cerebral palsy, quadriplegia or hemiplegia, and progressive cerebellar disease), organ kidney transplant, asplenia (due to splenectomy or spleen dysfunction, including sickle cell disease), rheumatoid arthritis/lupus/psoriasis, other immunosuppressive conditions, learning disability, several mental ill, the number of prior antibiotic prescription from one year and six week to six week before the index time (indicating the infection history)

eTable 12. Adjusted ORs of community-acquired sepsis 30-day mortality stratified by COVID-19 period (Complete risk analysis)

Group	Category	OR (Community + Hospital)	OR2 (Community)	OR3 (Hospital)
Age	18-39	0.20(0.16,0.24)	0.16(0.11,0.28)	0.17(0.14,0.26)
Age	40-49	0.65(0.58,0.73)	0.71(0.62,0.89)	0.75(0.62,0.83)
Age	60-69	1.38(1.25,1.43)	1.23(1.13,1.39)	1.24(1.19,1.33)
Age	70-79	1.85(1.67,1.96)	1.54(1.38,1.67)	1.59(1.45,1.78)
Age	80+	3.02(2.74,3.25)	2.48(2.26,2.67)	2.58(2.38,2.76)
Age	<18	0.19(0.11,0.22)	0.11(0.06,0.15)	0.09(0.05,0.17)
Antibiotic count	Antibiotic count: 1	0.99(0.91,1.07)	0.99(0.85,1.06)	0.97(0.94,1.02)
Antibiotic count	Antibiotic count: 2-3	1.04(0.96,1.04)	1.03(0.99,1.06)	1.04(0.97,1.02)
Antibiotic count	Antibiotic count: 3+	1.02(0.97,1.06)	1.03(0.98,1.13)	1.02(0.97,1.19)
Asthma	With no oral steroid use	0.85(0.78,0.84)	0.82(0.87,0.91)	0.85(0.84,0.99)
Asthma	With oral steroid use	0.89(0.72,0.94)	1.02(0.96,1.26)	0.97(0.86,1.09)
BMI	Obese I (30-34.9 kg/m2)	0.87(0.79,0.88)	0.82(0.78,0.92)	0.86(0.74,0.91)
BMI	Obese II (35-39.9 kg/m2)	0.82(0.73,0.96)	0.82(0.76,0.97)	0.89(0.86,0.91)
BMI	Obese III (40+ kg/m2)	0.91(0.88,1.01)	1.03(0.95,1.14)	0.98(0.95,1.09)
BMI	Overweight (25-29.9 kg/m2)	0.92(0.89,0.98)	0.88(0.81,0.96)	0.85(0.72,0.81)
BMI	Underweight (<18.5 kg/m2)	1.18(1.07,1.20)	1.15(1.06,1.28)	1.16(1.06,1.27)
CKD/RRT	CKD stage 3a	0.97(0.91,1.06)	1.03(0.95,1.05)	0.94(0.86,1.01)
CKD/RRT	CKD stage 3b	1.11(1.01,1.23)	1.29(1.18,1.37)	1.12(1.06,1.17)
CKD/RRT	CKD stage 4	1.51(1.43,1.67)	1.52(1.41,1.79)	1.43(1.32,1.53)
CKD/RRT	CKD stage 5	1.65(1.48,1.96)	1.75(1.44,2.24)	1.36(1.15,1.65)
CKD/RRT	RRT (dialysis)	1.23(1.02,1.41)	1.08(0.81,1.35)	0.82(0.66,1.05)
CKD/RRT	RRT (transplant)	0.58(0.39,0.74)	0.53(0.37,0.84)	0.66(0.46,0.89)
Diabetes	Controlled	0.96(0.91,0.98)	0.92(0.83,0.94)	0.94(0.89,0.98)
Diabetes	Not controlled	0.86(0.76,0.98)	0.84(0.61,1.04)	0.83(0.63,0.99)
Diabetes	Without recent Hb1ac measure	0.92(0.84,1.10)	0.99(0.84,1.17)	1.06(0.93,1.17)
Ethnicity	Black	0.79(0.66,0.84)	0.89(0.63,1.13)	0.64(0.52,0.71)
Ethnicity	Mixed	0.66(0.46,0.87)	1.09(0.79,1.52)	0.78(0.52,1.04)
Ethnicity	Other	0.81(0.73,1.02)	1.17(0.89,1.47)	1.04(0.88,1.34)
Ethnicity	South Asian	0.85(0.73,0.93)	0.92(0.88,1.05)	0.85(0.71,0.98)
IMD	IMD1(most deprived)	1.25(1.18,1.32)	1.21(1.12,1.32)	1.21(1.14,1.29)
IMD	IMD2	1.21(1.14,1.29)	1.16(1.07,1.25)	1.08(1.01,1.15)
IMD	IMD3	1.08(1.02,1.15)	1.06(0.99,1.16)	1.03(0.97,1.11)
IMD	IMD4	1.05(0.99,1.13)	1.02(0.95,1.12)	1.01(0.95,1.09)
Others	Alcohol problems	1.35(1.24,1.43)	1.41(1.36,1.53)	1.31(1.24,1.42)
Others	Asplenia	1.35(1.04,1.73)	1.28(0.93,1.79)	0.81(0.67,1.08)
Others	Cancer (non haematological)	1.28(1.26,1.32)	1.29(1.20,1.32)	1.26(1.24,1.37)
Others	Chronic cardiac disease	1.12(1.07,1.19)	1.13(1.06,1.23)	1.11(1.05,1.19)
Others	Chronic liver disease	1.82(1.67,2.24)	2.04(1.85,2.31)	2.01(1.88,2.23)
Others	Chronic respiratory disease	1.18(1.13,1.24)	1.22(1.27,1.31)	1.37(1.25,1.42)
Others	Dementia	1.09(0.97,1.19)	1.16(1.00,1.32)	1.17(1.05,1.21)
Others	Haematological malignancy	0.92(0.88,1.03)	0.94(0.84,1.06)	0.88(0.78,0.93)

Others	Hypertension	0.96(0.93,1.01)	0.99(0.97,1.03)	0.91(0.89,0.98)
Others	Immunosuppressive condition	1.08(0.96,1.14)	0.99(0.96,1.11)	0.92(0.87,1.03)
Others	Learning disability	1.34(1.18,1.69)	1.31(1.06,1.62)	0.98(0.85,1.15)
Others	Other neurological disease	0.96(0.93,1.05)	1.05(0.94,1.16)	1.06(0.93,1.11)
Others	Potential Care Home	0.88(0.74,0.83)	1.28(1.09,1.32)	1.28(1.19,1.42)
Others	Rheumatoid arthritis/ lupus/ psoriasis	1.06(0.98,1.07)	1.05(0.95,1.11)	1.06(0.98,1.16)
Others	Severe mental illness	0.96(0.81,1.01)	1.06(0.86,1.13)	0.97(0.89,1.06)
Others	Stroke	1.08(0.99,1.09)	0.97(0.91,1.07)	0.96(0.93,1.01)
Region	East Midlands	0.99(0.91,1.01)	1.11(1.06,1.27)	1.01(0.96,1.09)
Region	London	0.84(0.79,0.86)	0.91(0.82,1.14)	0.88(0.73,0.94)
Region	North East	1.03(0.93,1.14)	1.12(1.04,1.36)	1.02(0.93,1.17)
Region	North West	1.12(1.17,1.21)	1.25(1.14,1.36)	1.01(1.02,1.17)
Region	South East	0.76(0.73,0.82)	0.89(0.74,0.93)	0.80(0.75,0.87)
Region	South West	0.96(0.95,1.01)	1.05(0.98,1.18)	0.98(0.91,1.09)
Region	West Midlands	1.10(0.98,1.21)	1.34(1.17,1.54)	1.13(1.08,1.27)
Region	Yorkshire and the Humber	1.08(1.06,1.13)	1.23(1.19,1.35)	1.13(1.10,1.21)
Sex	Male	0.96(0.82,1.01)	0.96(0.92,1.02)	0.95(0.92,1.02)
Smoking	Current	1.55(1.43,1.62)	1.69(1.44,1.74)	1.63(1.57,1.76)
Smoking	Former	1.04(1.01,1.18)	1.10(1.04,1.12)	1.17(1.06,1.29)
Tx	Kidney transplant	0.61(0.47,0.76)	0.56(0.32,0.75)	0.56(0.46,0.78)
Tx	Other organ transplant	0.99(0.66,1.45)	0.45(0.13,0.96)	0.85(0.41,1.33)

Models were adjusted for age using a 4-knot cubic spline, except for estimation of age group relative odds of 30-day mortality; and adjusted for sex, except for estimation of sex group relative odds of 30-day mortality; and stratified by region, except for IMD group relative odds of 30-day mortality.
Abbreviations: IMD, index of multiple deprivation; BMI, body mass index;
Tx, transplant; CKD, chronic kidney disease; RRT, renal replacement therapy.

The number of prior antibiotic prescriptions (one year plus six weeks to six weeks before the index date)

Reference sub-group:
age: 50-59 years
sex: female
region: East of England
IMD: the least deprived quintile
ethnicity: white
BMI: healthy range (18.5 – 24.9 kg/m2)
smoking: never
prior antibiotic count: 0
Patients without the disease were used as the reference for other clinical conditions.

eTable 13. Adjusted ORs of community-acquired sepsis 30-day mortality (COVID-19 period as a moderator)

Group	Category	OR (95% CI)
Age	< 18	0.13(0.12,0.14)
Age	18-39	0.17(0.12,0.22)
Age	40-49	0.21(0.18,0.24)
Age	60-69	1.37(1.27,1.47)
Age	70-79	1.84(1.72,1.97)
Age	> 80	3.10(2.90,3.31)
Antibiotic count	Antibiotic count: 1	1.00(0.95,1.04)
Antibiotic count	Antibiotic count: 2-3	1.02(0.97,1.06)
Antibiotic count	Antibiotic count: 3+	1.04(0.99,1.08)
Asthma	With no oral steroid use	0.85(0.81,0.90)
Asthma	With oral steroid use	0.89(0.81,0.98)
BMI	Obese I (30-34.9 kg/m2)	0.84(0.80,0.88)
BMI	Obese II (35-39.9 kg/m2)	0.85(0.79,0.92)
BMI	Obese III (40+ kg/m2)	0.94(0.86,1.02)
BMI	Overweight (25-29.9 kg/m2)	0.92(0.88,0.96)
BMI	Underweight (<18.5 kg/m2)	1.10(1.02,1.18)
CKD/RRT	CKD stage 3a	0.98(0.94,1.03)
CKD/RRT	CKD stage 3b	1.17(1.12,1.23)
CKD/RRT	CKD stage 4	1.57(1.47,1.68)
CKD/RRT	CKD stage 5	1.69(1.46,1.93)
CKD/RRT	RRT (dialysis)	1.27(1.10,1.46)
CKD/RRT	RRT (transplant)	0.61(0.47,0.79)
Diabetes	Controlled	0.96(0.93,0.99)
Diabetes	Not controlled	0.89(0.78,1.00)
Diabetes	Without recent Hb1ac measure	1.02(0.93,1.12)
Ethnicity	Black	0.82(0.69,0.97)
Ethnicity	Mixed	0.74(0.55,0.97)
Ethnicity	Other	0.83(0.67,1.00)
Ethnicity	South Asian	0.79(0.72,0.87)
IMD	IMD1(most deprived)	1.18(1.12,1.24)
IMD	IMD2	1.20(1.14,1.26)
IMD	IMD3	1.09(1.04,1.15)
IMD	IMD4	1.06(1.01,1.12)
Others	Alcohol problems	1.33(1.26,1.39)
Others	Asplenia	1.30(1.06,1.58)
Others	Cancer (non haematological)	1.21(1.17,1.26)
Others	Chronic cardiac disease	1.16(1.13,1.20)
Others	Chronic liver disease	1.82(1.67,1.98)
Others	Chronic respiratory disease	1.21(1.17,1.26)
Others	Dementia	1.10(1.01,1.19)
Others	Haematological malignancy	0.90(0.84,0.96)
Others	Hypertension	1.01(0.97,1.04)
Others	Immunosuppressive condition	0.98(0.92,1.03)
Others	Learning disability	1.36(1.17,1.58)
Others	Other neurological disease	1.00(0.93,1.07)
Others	Potential Care Home	0.81(0.77,0.86)

Others	Rheumatoid arthritis/ lupus/ psoriasis	1.03(0.98,1.09)
Others	Severe mental illness	0.93(0.84,1.03)
Others	Stroke	1.07(1.02,1.11)
Region	East Midlands	0.96(0.92,1.01)
Region	London	0.84(0.77,0.91)
Region	North East	1.06(0.98,1.15)
Region	North West	1.15(1.08,1.22)
Region	South East	0.80(0.75,0.86)
Region	South West	0.98(0.93,1.04)
Region	West Midlands	1.13(1.03,1.23)
Region	Yorkshire and the Humber	1.10(1.04,1.16)
Sex	Male	1.00(0.97,1.03)
Smoking	Current	1.60(1.51,1.68)
Smoking	Former	1.10(1.06,1.14)
Tx	Kidney transplant	0.70(0.56,0.86)
Tx	Other organ transplant	0.96(0.66,1.37)

Models were adjusted for age using a 4-knot cubic spline, except for estimation of age group relative odds of 30-day mortality; and adjusted for sex, except for estimation of sex group relative odds of 30-day mortality; and stratified by region, except for IMD group relative odds of 30-day mortality.
Abbreviations: IMD, index of multiple deprivation; BMI, body mass index;
Tx, transplant; CKD, chronic kidney disease; RRT, renal replacement therapy.

The relative odds of 30-day mortality presented in this figure can be found in table S6 of the Supplementary material.

The number of prior antibiotic prescriptions (one year plus six weeks to six weeks before the index date)

Reference sub-group:
age: 50-59 years
sex: female
region: East of England
IMD: the least deprived quintile
ethnicity: white
BMI: healthy range (18.5 – 24.9 kg/m2)
smoking: never
prior antibiotic count: 0
Patients without the disease were used as the reference for other clinical conditions.

eTable 14. The underlying number of Figure 2

		Community + hospital		community		hospital		
Calendar month	IMD	count	incidence rate	count	incidence rate	count	incidence rate	population
01/01/2019	1(most deprived)	1965	0.448	1595	0.364	365	0.083	4386440
01/01/2019	2	1915	0.445	1555	0.361	360	0.084	4303400
01/01/2019	3	1885	0.407	1495	0.323	390	0.084	4630650
01/01/2019	4	1680	0.384	1315	0.3	360	0.082	4378515
01/01/2019	5(least deprived)	1510	0.368	1170	0.285	340	0.083	4104170
01/02/2019	1(most deprived)	1660	0.378	1340	0.305	320	0.073	4392915
01/02/2019	2	1505	0.349	1195	0.277	310	0.072	4309910
01/02/2019	3	1625	0.35	1270	0.274	350	0.075	4637510
01/02/2019	4	1490	0.34	1220	0.278	270	0.062	4384475
01/02/2019	5(least deprived)	1255	0.306	970	0.236	280	0.068	4107515
01/03/2019	1(most deprived)	1670	0.38	1345	0.306	325	0.074	4397065
01/03/2019	2	1580	0.366	1275	0.295	305	0.071	4316175
01/03/2019	3	1630	0.351	1280	0.276	350	0.075	4641915
01/03/2019	4	1525	0.348	1190	0.271	330	0.075	4387325
01/03/2019	5(least deprived)	1290	0.314	1010	0.246	280	0.068	4108040
01/04/2019	1(most deprived)	1635	0.372	1325	0.301	310	0.07	4398475
01/04/2019	2	1480	0.343	1185	0.274	295	0.068	4318495
01/04/2019	3	1665	0.359	1300	0.28	365	0.079	4641910
01/04/2019	4	1550	0.353	1220	0.278	330	0.075	4387120
01/04/2019	5(least deprived)	1270	0.309	995	0.242	275	0.067	4107460
01/05/2019	1(most deprived)	1650	0.374	1315	0.298	340	0.077	4408515
01/05/2019	2	1465	0.338	1180	0.273	285	0.066	4328265
01/05/2019	3	1590	0.342	1270	0.273	320	0.069	4649560
01/05/2019	4	1495	0.34	1150	0.262	345	0.079	4394270
01/05/2019	5(least deprived)	1215	0.295	945	0.23	270	0.066	4112875
01/06/2019	1(most deprived)	1455	0.33	1195	0.271	260	0.059	4414400
01/06/2019	2	1425	0.329	1140	0.263	285	0.066	4334510
01/06/2019	3	1520	0.326	1190	0.256	335	0.072	4655555
01/06/2019	4	1430	0.325	1120	0.255	310	0.07	4399275
01/06/2019	5(least deprived)	1115	0.271	845	0.205	265	0.064	4116645
01/07/2019	1(most deprived)	1625	0.368	1290	0.292	340	0.077	4416270
01/07/2019	2	1545	0.356	1240	0.286	305	0.07	4336400
01/07/2019	3	1595	0.342	1290	0.277	305	0.065	4657600
01/07/2019	4	1430	0.325	1140	0.259	290	0.066	4400790
01/07/2019	5(least deprived)	1300	0.316	1010	0.245	290	0.07	4117830
01/08/2019	1(most deprived)	1520	0.344	1205	0.272	315	0.071	4423065
01/08/2019	2	1445	0.333	1150	0.265	300	0.069	4341670
01/08/2019	3	1585	0.34	1240	0.266	345	0.074	4662925
01/08/2019	4	1440	0.327	1115	0.253	320	0.073	4405960
01/08/2019	5(least deprived)	1300	0.315	1005	0.244	295	0.072	4121995
01/09/2019	1(most deprived)	1455	0.328	1170	0.264	285	0.064	4430425
01/09/2019	2	1385	0.319	1105	0.254	280	0.064	4347850

01/09/2019	3	1460	0.313	1185	0.254	275	0.059	4669755
01/09/2019	4	1360	0.308	1075	0.244	285	0.065	4412485
01/09/2019	5(least deprived)	1235	0.299	980	0.237	255	0.062	4128345
01/10/2019	1(most deprived)	1515	0.342	1220	0.275	295	0.067	4431635
01/10/2019	2	1385	0.318	1110	0.255	270	0.062	4352525
01/10/2019	3	1480	0.317	1180	0.253	305	0.065	4670350
01/10/2019	4	1345	0.305	1040	0.236	305	0.069	4413530
01/10/2019	5(least deprived)	1180	0.286	900	0.218	280	0.068	4126135
01/11/2019	1(most deprived)	1370	0.309	1115	0.251	255	0.057	4440560
01/11/2019	2	1390	0.319	1125	0.258	265	0.061	4362020
01/11/2019	3	1400	0.299	1125	0.24	275	0.059	4681260
01/11/2019	4	1305	0.295	1025	0.232	280	0.063	4421960
01/11/2019	5(least deprived)	1160	0.281	920	0.223	235	0.057	4132870
01/12/2019	1(most deprived)	1455	0.327	1190	0.268	265	0.06	4445405
01/12/2019	2	1510	0.346	1220	0.279	290	0.066	4368410
01/12/2019	3	1595	0.34	1285	0.274	305	0.065	4686565
01/12/2019	4	1365	0.308	1070	0.242	295	0.067	4427140
01/12/2019	5(least deprived)	1150	0.278	920	0.222	230	0.056	4136235
01/01/2020	1(most deprived)	1360	0.306	1130	0.254	230	0.052	4445480
01/01/2020	2	1360	0.311	1080	0.247	280	0.064	4368335
01/01/2020	3	1430	0.305	1145	0.244	290	0.062	4686390
01/01/2020	4	1365	0.308	1100	0.248	265	0.06	4427860
01/01/2020	5(least deprived)	1115	0.27	880	0.213	240	0.058	4135515
01/02/2020	1(most deprived)	1170	0.263	930	0.209	235	0.053	4454015
01/02/2020	2	1230	0.281	970	0.222	260	0.059	4377410
01/02/2020	3	1185	0.252	950	0.202	235	0.05	4694790
01/02/2020	4	1035	0.233	825	0.186	210	0.047	4434515
01/02/2020	5(least deprived)	960	0.232	735	0.178	225	0.054	4140550
01/03/2020	1(most deprived)	800	0.179	640	0.144	155	0.035	4458125
01/03/2020	2	765	0.175	610	0.139	155	0.035	4381935
01/03/2020	3	830	0.177	645	0.137	185	0.039	4699120
01/03/2020	4	810	0.183	630	0.142	175	0.039	4437775
01/03/2020	5(least deprived)	625	0.151	500	0.121	125	0.03	4142225
01/04/2020	1(most deprived)	540	0.121	460	0.103	80	0.018	4465285
01/04/2020	2	565	0.129	500	0.114	70	0.016	4389060
01/04/2020	3	550	0.117	450	0.096	100	0.021	4704765
01/04/2020	4	520	0.117	435	0.098	85	0.019	4442745
01/04/2020	5(least deprived)	440	0.106	360	0.087	80	0.019	4146210
01/05/2020	1(most deprived)	815	0.182	685	0.153	130	0.029	4477995
01/05/2020	2	715	0.162	600	0.136	115	0.026	4401530
01/05/2020	3	775	0.164	645	0.137	130	0.028	4716780
01/05/2020	4	705	0.158	595	0.134	110	0.025	4453025
01/05/2020	5(least deprived)	635	0.153	520	0.125	115	0.028	4154645
01/06/2020	1(most deprived)	890	0.198	730	0.163	160	0.036	4490650
01/06/2020	2	940	0.213	790	0.179	145	0.033	4413775
01/06/2020	3	955	0.202	800	0.169	155	0.033	4729205

01/06/2020	4	805	0.18	640	0.143	160	0.036	4463340
01/06/2020	5(least deprived)	730	0.175	595	0.143	135	0.032	4163160
01/07/2020	1(most deprived)	1040	0.231	870	0.193	170	0.038	4506290
01/07/2020	2	1040	0.235	835	0.189	205	0.046	4426450
01/07/2020	3	1175	0.248	940	0.198	235	0.05	4740830
01/07/2020	4	1005	0.225	785	0.175	220	0.049	4473985
01/07/2020	5(least deprived)	825	0.198	660	0.158	165	0.04	4172040
01/08/2020	1(most deprived)	1135	0.251	935	0.207	200	0.044	4520120
01/08/2020	2	1170	0.264	960	0.216	205	0.046	4439075
01/08/2020	3	1260	0.265	1020	0.215	240	0.05	4752795
01/08/2020	4	1135	0.253	920	0.205	215	0.048	4484735
01/08/2020	5(least deprived)	940	0.225	730	0.175	210	0.05	4181465
01/09/2020	1(most deprived)	1045	0.231	860	0.19	185	0.041	4526170
01/09/2020	2	1100	0.247	900	0.202	200	0.045	4444825
01/09/2020	3	1090	0.229	880	0.185	210	0.044	4757860
01/09/2020	4	990	0.22	805	0.179	185	0.041	4490400
01/09/2020	5(least deprived)	895	0.214	665	0.159	230	0.055	4186700
01/10/2020	1(most deprived)	970	0.214	795	0.175	175	0.039	4536530
01/10/2020	2	895	0.201	715	0.16	185	0.042	4456980
01/10/2020	3	1045	0.219	835	0.175	210	0.044	4768475
01/10/2020	4	915	0.203	710	0.158	205	0.046	4496980
01/10/2020	5(least deprived)	780	0.186	620	0.148	160	0.038	4191605
01/11/2020	1(most deprived)	735	0.162	590	0.13	150	0.033	4548750
01/11/2020	2	780	0.174	630	0.141	150	0.034	4470945
01/11/2020	3	790	0.165	645	0.135	145	0.03	4781010
01/11/2020	4	755	0.167	600	0.133	150	0.033	4507555
01/11/2020	5(least deprived)	610	0.145	470	0.112	140	0.033	4200485
01/12/2020	1(most deprived)	705	0.155	580	0.127	130	0.029	4553855
01/12/2020	2	710	0.159	555	0.124	150	0.034	4475285
01/12/2020	3	775	0.162	625	0.131	150	0.031	4784395
01/12/2020	4	720	0.16	580	0.129	140	0.031	4510095
01/12/2020	5(least deprived)	560	0.133	440	0.105	120	0.029	4201745
01/01/2021	1(most deprived)	695	0.152	575	0.126	120	0.026	4559760
01/01/2021	2	710	0.159	595	0.133	115	0.026	4478800
01/01/2021	3	730	0.153	615	0.128	120	0.025	4786370
01/01/2021	4	660	0.146	545	0.121	115	0.025	4510635
01/01/2021	5(least deprived)	550	0.131	455	0.108	95	0.023	4201845
01/02/2021	1(most deprived)	825	0.181	685	0.15	140	0.031	4563670
01/02/2021	2	740	0.165	605	0.135	135	0.03	4479870
01/02/2021	3	870	0.182	710	0.148	160	0.033	4784095
01/02/2021	4	785	0.174	635	0.141	155	0.034	4506620
01/02/2021	5(least deprived)	640	0.153	510	0.122	135	0.032	4196310
01/03/2021	1(most deprived)	1050	0.23	850	0.186	200	0.044	4565725
01/03/2021	2	985	0.22	800	0.179	185	0.041	4479245
01/03/2021	3	1095	0.229	865	0.181	225	0.047	4780435
01/03/2021	4	950	0.211	750	0.167	200	0.044	4502970

01/03/2021	5(least deprived)	795	0.19	635	0.151	160	0.038	4191510
01/04/2021	1(most deprived)	1150	0.252	945	0.207	205	0.045	4555625
01/04/2021	2	1035	0.232	830	0.186	205	0.046	4465770
01/04/2021	3	1095	0.23	865	0.182	230	0.048	4765615
01/04/2021	4	990	0.22	775	0.173	215	0.048	4490420
01/04/2021	5(least deprived)	865	0.207	675	0.161	190	0.045	4181850
01/05/2021	1(most deprived)	1170	0.258	955	0.21	215	0.047	4541775
01/05/2021	2	1190	0.267	955	0.215	235	0.053	4451305
01/05/2021	3	1155	0.243	930	0.196	225	0.047	4750140
01/05/2021	4	1055	0.236	850	0.19	200	0.045	4476915
01/05/2021	5(least deprived)	930	0.223	720	0.173	210	0.05	4170325
01/06/2021	1(most deprived)	1135	0.251	940	0.208	190	0.042	4524915
01/06/2021	2	1145	0.258	910	0.205	235	0.053	4433555
01/06/2021	3	1180	0.249	940	0.199	240	0.051	4732030
01/06/2021	4	1060	0.238	820	0.184	240	0.054	4459145
01/06/2021	5(least deprived)	865	0.208	675	0.162	190	0.046	4154755
01/07/2021	1(most deprived)	1150	0.255	925	0.205	225	0.05	4508825
01/07/2021	2	1115	0.252	875	0.198	240	0.054	4416830
01/07/2021	3	1095	0.232	890	0.189	205	0.043	4715560
01/07/2021	4	1035	0.233	815	0.183	220	0.049	4444790
01/07/2021	5(least deprived)	890	0.215	700	0.169	190	0.046	4142580
01/08/2021	1(most deprived)	995	0.221	780	0.173	215	0.048	4503720
01/08/2021	2	1065	0.242	870	0.197	195	0.044	4409405
01/08/2021	3	1130	0.24	895	0.19	235	0.05	4711070
01/08/2021	4	995	0.224	780	0.176	215	0.048	4441220
01/08/2021	5(least deprived)	855	0.207	675	0.163	175	0.042	4139510
01/09/2021	1(most deprived)	1055	0.235	840	0.187	215	0.048	4496075
01/09/2021	2	990	0.225	810	0.184	180	0.041	4399840
01/09/2021	3	1040	0.221	805	0.171	235	0.05	4703900
01/09/2021	4	910	0.205	730	0.165	180	0.041	4435805
01/09/2021	5(least deprived)	800	0.193	640	0.155	160	0.039	4135535
01/10/2021	1(most deprived)	970	0.216	830	0.185	135	0.03	4492800
01/10/2021	2	955	0.217	790	0.18	165	0.038	4397485
01/10/2021	3	1080	0.23	875	0.186	205	0.044	4701710
01/10/2021	4	930	0.21	730	0.165	200	0.045	4433150
01/10/2021	5(least deprived)	805	0.195	640	0.155	170	0.041	4130170
01/11/2021	1(most deprived)	945	0.21	775	0.173	165	0.037	4492060
01/11/2021	2	920	0.209	745	0.169	175	0.04	4398120
01/11/2021	3	990	0.211	810	0.172	180	0.038	4702975
01/11/2021	4	945	0.213	780	0.176	165	0.037	4432875
01/11/2021	5(least deprived)	725	0.176	575	0.139	150	0.036	4129350
01/12/2021	1(most deprived)	920	0.205	765	0.171	155	0.035	4483130
01/12/2021	2	845	0.192	690	0.157	155	0.035	4396715
01/12/2021	3	1005	0.214	825	0.175	180	0.038	4701180
01/12/2021	4	860	0.194	690	0.156	170	0.038	4429035
01/12/2021	5(least deprived)	725	0.176	585	0.142	140	0.034	4127320

01/01/2022	1(most deprived)	860	0.192	715	0.159	145	0.032	4483010
01/01/2022	2	860	0.196	720	0.164	140	0.032	4397300
01/01/2022	3	910	0.194	760	0.162	150	0.032	4702040
01/01/2022	4	830	0.187	680	0.153	150	0.034	4430320
01/01/2022	5(least deprived)	675	0.164	555	0.134	120	0.029	4127915
01/02/2022	1(most deprived)	805	0.179	675	0.151	125	0.028	4484865
01/02/2022	2	765	0.174	630	0.143	130	0.03	4402690
01/02/2022	3	815	0.173	655	0.139	160	0.034	4708190
01/02/2022	4	790	0.178	625	0.141	165	0.037	4437200
01/02/2022	5(least deprived)	655	0.158	530	0.128	130	0.031	4133115
01/03/2022	1(most deprived)	865	0.194	720	0.161	145	0.032	4463290
01/03/2022	2	800	0.182	655	0.149	145	0.033	4403500
01/03/2022	3	840	0.178	675	0.143	165	0.035	4711140
01/03/2022	4	790	0.178	645	0.145	150	0.034	4442520
01/03/2022	5(least deprived)	695	0.168	545	0.132	155	0.037	4139760
01/04/2022	1(most deprived)	925	0.207	770	0.172	155	0.035	4473470
01/04/2022	2	815	0.184	655	0.148	165	0.037	4418810
01/04/2022	3	885	0.187	730	0.154	155	0.033	4727410
01/04/2022	4	820	0.184	635	0.142	185	0.041	4459075
01/04/2022	5(least deprived)	660	0.159	525	0.126	135	0.033	4152850
01/05/2022	1(most deprived)	965	0.215	800	0.178	165	0.037	4481920
01/05/2022	2	940	0.212	770	0.174	170	0.038	4429340
01/05/2022	3	965	0.204	780	0.165	185	0.039	4738415
01/05/2022	4	895	0.2	715	0.16	175	0.039	4469625
01/05/2022	5(least deprived)	795	0.191	625	0.15	165	0.04	4161795
01/06/2022	1(most deprived)	920	0.205	775	0.173	150	0.033	4484315
01/06/2022	2	865	0.195	695	0.157	170	0.038	4435285
01/06/2022	3	925	0.195	775	0.163	155	0.033	4744825
01/06/2022	4	845	0.189	685	0.153	165	0.037	4476915
01/06/2022	5(least deprived)	710	0.17	550	0.132	160	0.038	4167785

To reduce the risk of secondary disclosure, all counted numbers in the baseline table were rounded to the nearest five

eTable 15. ORs of developing non-COVID-19 sepsis for factors stratified by sepsis type (Multiple imputation)

	**OR (Community + Hospital)**	**OR2 (Community)**	**OR3 (Hospital)**
IMD			
IMD 1 (Most deprived)	1.80 (1.77-1.83)	1.90 (1.87-1.93)	1.44 (1.39-1.49)
IMD 2	1.48 (1.46-1.50)	1.54 (1.51-1.56)	1.27 (1.23-1.31)
IMD 3	1.25 (1.23-1.27)	1.28 (1.26-1.30)	1.14 (1.11-1.18)
IMD 4	1.14 (1.12-1.16)	1.15 (1.13-1.17)	1.10 (1.06-1.13)
Ethnicity			
Mixed	0.98 (0.92-1.04)	0.97 (0.91-1.04)	0.99 (0.88-1.11)
South Asian	1.09 (1.06-1.11)	1.10 (1.07-1.13)	1.08 (1.03-1.13)
Black	0.97 (0.93-1.01)	0.99 (0.94-1.03)	0.91 (0.84-0.99)
Other	0.81 (0.77-0.85)	0.80 (0.75-0.84)	0.85 (0.77-0.94)
BMI			
Underweight (<18.5 kg/m2)	1.79 (1.75-1.83)	1.68 (1.64-1.73)	1.69 (1.59-1.80)
Overweight (25-29.9 kg/m2)	0.88 (0.87-0.89)	0.87 (0.86-0.88)	0.86 (0.83-0.88)
Obese I (30-34.9 kg/m2)	1.06 (1.05-1.08)	1.06 (1.04-1.08)	0.94 (0.91-0.98)
Obese II (35-39.9 kg/m2)	1.38 (1.35-1.41)	1.44 (1.40-1.47)	1.16 (1.11-1.21)
Obese III (40+ kg/m2)	2.23 (2.17-2.28)	2.44 (2.38-2.51)	1.30 (1.23-1.37)
Smoking			
Former	1.34 (1.33-1.35)	1.34 (1.33-1.36)	1.34 (1.31-1.37)
Current	1.79 (1.76-1.81)	1.89 (1.86-1.93)	1.44 (1.39-1.48)

Crude ORs of sepsis by IMD quintile, ethnicity, BMI, smoking history and stratified by type of sepsis

Reference groups:
IMD quintile: the least deprived quintile (IMD 5)
Ethnicity: white
BMI: healthy range (18.5 – 24.9 kg/m2)
Smoking: never (Smoking status identified from the most recent clinical records)

Abbreviations:
IMD (Index of Multiple Deprivation) quintile measured from patient-level address
BMI, Body Mass Index (from the most recent clinical records)

eTable 16. ORs of developing non-COVID-19 sepsis for factors stratified by sepsis type (Multiple imputation, 18+ years old study population)

	OR (Community + Hospital)	OR2 (Community)	OR3 (Hospital)
IMD			
IMD 1 (Most deprived)	1.81 (1.78-1.84)	1.91 (1.88-1.95)	1.45 (1.40-1.50)
IMD 2	1.48 (1.46-1.51)	1.54 (1.52-1.57)	1.28 (1.23-1.32)
IMD 3	1.25 (1.23-1.27)	1.28 (1.26-1.30)	1.14 (1.11-1.18)
IMD 4	1.14 (1.12-1.16)	1.15 (1.13-1.17)	1.10 (1.06-1.13)
Ethnicity			
Mixed	0.98 (0.92-1.06)	0.98 (0.91-1.05)	1.00 (0.88-1.13)
South Asian	1.08 (1.05-1.10)	1.09 (1.06-1.12)	1.06 (1.01-1.11)
Black	0.97 (0.93-1.01)	0.99 (0.94-1.03)	0.92 (0.84-1.01)
Other	0.80 (0.76-0.84)	0.79 (0.75-0.84)	0.84 (0.76-0.93)
BMI			
Underweight (<18.5 kg/m2)	1.79 (1.75-1.83)	1.71 (1.66-1.75)	1.65 (1.55-1.75)
Overweight (25-29.9 kg/m2)	0.87 (0.86-0.88)	0.84 (0.83-0.85)	0.85 (0.83-0.88)
Obese I (30-34.9 kg/m2)	1.06 (1.05-1.08)	1.07 (1.05-1.08)	0.94 (0.91-0.97)
Obese II (35-39.9 kg/m2)	1.36 (1.33-1.39)	1.43 (1.40-1.46)	1.15 (1.10-1.20)
Obese III (40+ kg/m2)	2.25 (2.19-2.30)	2.38 (2.32-2.44)	1.30 (1.23-1.38)
Smoking			
Former	1.35 (1.34-1.36)	1.35 (1.33-1.37)	1.35 (1.32-1.38)
Current	1.80 (1.77-1.83)	1.91 (1.88-1.94)	1.45 (1.40-1.50)

Crude ORs of sepsis by IMD quintile, ethnicity, BMI, smoking history and stratified by type of sepsis

Reference groups:
IMD quintile: the least deprived quintile (IMD 5)
Ethnicity: white
BMI: healthy range (18.5 – 24.9 kg/m2)
Smoking: never (Smoking status identified from the most recent clinical records)

Abbreviations:
IMD (Index of Multiple Deprivation) quintile measured from patient-level address
BMI, Body Mass Index (from the most recent clinical records)

eTable 17. Adjusted ORs of developing non-COVID-19 sepsis for clinical characteristics stratified by sepsis type (Multiple imputation)

type	OR (Community + Hospital)	OR2 (Community)	OR3 (Hospital)
Potential Care Home	2.34 (2.28-2.40)	2.32 (2.26-2.38)	2.30 (2.11-2.50)
CKD stage 3a	1.24 (1.23-1.26)	1.24 (1.22-1.26)	1.27 (1.22-1.33)
CKD stage 3b	1.70 (1.66-1.73)	1.68 (1.65-1.72)	1.82 (1.72-1.92)
CKD stage 4	2.62 (2.55-2.70)	2.58 (2.51-2.66)	3.11 (2.86-3.39)
CKD stage 5	6.23 (5.81-6.69)	5.79 (5.37-6.24)	11.29 (9.34-13.65)
RRT (dialysis)	7.29 (6.79-7.83)	6.17 (5.70-6.68)	18.15 (15.34-21.48)
RRT (transplant)	5.45 (4.32-6.88)	4.82 (3.69-6.28)	10.80 (6.46-18.08)
With no oral steroid use	0.99 (0.98-1.01)	1.00 (0.98-1.02)	1.00 (0.97-1.04)
With oral steroid use	0.92 (0.89-0.95)	0.93 (0.89-0.96)	0.88 (0.81-0.95)
Controlled	1.45 (1.43-1.47)	1.47 (1.46-1.49)	1.35 (1.30-1.39)
Not controlled	2.39 (2.29-2.50)	2.47 (2.36-2.59)	2.07 (1.84-2.34)
Without recent Hb1ac measure	1.58 (1.54-1.63)	1.62 (1.57-1.67)	1.38 (1.28-1.49)
Kidney transplant	1.50 (1.21-1.85)	1.75 (1.37-2.23)	0.79 (0.50-1.25)
Other organ transplant	1.67 (1.43-1.94)	1.71 (1.44-2.02)	1.66 (1.18-2.33)
Hypertension	1.13 (1.11-1.14)	1.14 (1.13-1.16)	1.06 (1.03-1.09)
Chronic respiratory disease	1.44 (1.42-1.46)	1.45 (1.43-1.47)	1.41 (1.36-1.47)
Chronic cardiac disease	1.39 (1.37-1.41)	1.37 (1.35-1.39)	1.56 (1.51-1.61)
Cancer (non haematological)	2.37 (2.34-2.40)	1.74 (1.71-1.76)	7.94 (7.71-8.17)
Haematological malignancy	2.53 (2.43-2.63)	1.80 (1.72-1.89)	4.72 (4.32-5.15)
Chronic liver disease	3.08 (2.97-3.19)	3.08 (2.96-3.20)	3.43 (3.13-3.76)
Stroke	1.48 (1.45-1.50)	1.48 (1.46-1.51)	1.43 (1.37-1.51)
Dementia	1.42 (1.37-1.47)	1.44 (1.39-1.49)	1.11 (0.98-1.25)
Other neurological disease	2.33 (2.28-2.39)	2.43 (2.37-2.50)	1.92 (1.78-2.07)
Asplenia	1.13 (1.05-1.21)	1.13 (1.04-1.23)	1.31 (1.10-1.55)
Rheumatoid arthritis/ lupus/ psoriasis	1.25 (1.23-1.27)	1.26 (1.23-1.28)	1.23 (1.17-1.28)
Immunosuppressive condition	2.09 (2.02-2.16)	1.64 (1.58-1.70)	5.15 (4.79-5.54)
Learning disability	3.53 (3.35-3.73)	3.90 (3.68-4.14)	2.45 (2.12-2.83)
Severe mental illness	1.96 (1.89-2.03)	2.06 (1.99-2.14)	1.52 (1.38-1.67)
Alcohol problems	1.37 (1.35-1.39)	1.41 (1.38-1.43)	1.25 (1.20-1.30)
Antibiotic count: 1	1.73 (1.70-1.75)	1.69 (1.67-1.72)	1.86 (1.80-1.93)
Antibiotic count: 2-3	2.30 (2.27-2.34)	2.28 (2.24-2.32)	2.41 (2.32-2.50)
Antibiotic count: 3+	3.38 (3.33-3.43)	3.39 (3.33-3.45)	3.41 (3.37-3.55)

Reference groups:
Clinical characteristics: the patients without the clinical disease.
The number of prior antibiotic prescriptions: antibiotic count: 0.
Models were adjusted for all comorbidities*

All comorbidities* Hypertension, chronic cardiac disease, diabetes, stroke, chronic kidney disease or renal replacement therapy) and asthma, cancer (non-haematological and haematological), chronic liver disease, dementia, other neurological disease (including motor neuron disease, myasthenia gravis, multiple sclerosis, Parkinson's disease, cerebral palsy, quadriplegia or hemiplegia, and progressive cerebellar disease), organ kidney transplant, asplenia (due to splenectomy or spleen dysfunction, including sickle cell disease), rheumatoid arthritis/lupus/psoriasis, other immunosuppressive conditions, learning disability, several mental ill, the number of prior antibiotic prescription from one year and six week to six week before the index time (indicating the infection history)

Abbreviations:
CKD, chronic kidney disease; RRT, renal replacement therapy.
The number of prior antibiotic prescriptions (one year plus six weeks to six weeks before the index date)

eTable 18. Adjusted ORs of developing non-COVID-19 sepsis for clinical characteristics stratified by sepsis type (Multiple imputation, 18+ years old study population)

type	OR (Community + Hospital)	OR2 (Community)	OR3 (Hospital)
Potential Care Home	2.34 (2.28-2.40)	2.32 (2.26-2.38)	2.30 (2.11-2.50)
CKD stage 3a	1.24 (1.23-1.26)	1.24 (1.22-1.26)	1.27 (1.22-1.33)
CKD stage 3b	1.70 (1.67-1.73)	1.68 (1.65-1.72)	1.82 (1.72-1.92)
CKD stage 4	2.62 (2.55-2.70)	2.58 (2.51-2.66)	3.10 (2.85-3.38)
CKD stage 5	6.23 (5.81-6.68)	5.79 (5.37-6.24)	11.15 (9.23-13.47)
RRT (dialysis)	7.22 (6.73-7.75)	6.13 (5.66-6.64)	17.71 (14.97-20.94)
RRT (transplant)	5.39 (4.27-6.80)	4.80 (3.68-6.25)	10.29 (6.15-17.21)
With no oral steroid use	0.99 (0.98-1.01)	1.00 (0.98-1.01)	1.00 (0.96-1.04)
With oral steroid use	0.91 (0.89-0.94)	0.92 (0.89-0.96)	0.88 (0.81-0.95)
Controlled	1.45 (1.43-1.47)	1.47 (1.45-1.49)	1.35 (1.30-1.39)
Not controlled	2.39 (2.28-2.49)	2.47 (2.36-2.59)	2.07 (1.84-2.33)
Without recent Hb1ac measure	1.58 (1.53-1.62)	1.61 (1.56-1.66)	1.38 (1.28-1.49)
Kidney transplant	1.49 (1.21-1.85)	1.74 (1.37-2.22)	0.79 (0.50-1.25)
Other organ transplant	1.55 (1.33-1.81)	1.62 (1.36-1.92)	1.44 (1.02-2.04)
Hypertension	1.12 (1.11-1.14)	1.14 (1.13-1.16)	1.06 (1.02-1.09)
Chronic respiratory disease	1.44 (1.41-1.46)	1.45 (1.43-1.47)	1.41 (1.36-1.47)
Chronic cardiac disease	1.38 (1.37-1.40)	1.37 (1.35-1.38)	1.55 (1.50-1.60)
Cancer (non haematological)	2.35 (2.32-2.38)	1.73 (1.71-1.76)	7.73 (7.51-7.96)
Haematological malignancy	2.47 (2.37-2.57)	1.79 (1.70-1.88)	4.58 (4.19-5.00)
Chronic liver disease	3.06 (2.95-3.17)	3.07 (2.95-3.19)	3.38 (3.08-3.71)
Stroke	1.47 (1.45-1.50)	1.48 (1.46-1.51)	1.43 (1.36-1.50)
Dementia	1.41 (1.36-1.47)	1.44 (1.39-1.49)	1.10 (0.97-1.25)
Other neurological disease	2.30 (2.24-2.36)	2.41 (2.35-2.48)	1.86 (1.72-2.01)
Asplenia	1.11 (1.03-1.19)	1.12 (1.03-1.21)	1.27 (1.07-1.51)
Rheumatoid arthritis/ lupus/ psoriasis	1.25 (1.23-1.27)	1.26 (1.24-1.28)	1.22 (1.17-1.28)
Immunosuppressive condition	2.05 (1.98-2.12)	1.61 (1.55-1.67)	5.01 (4.66-5.39)
Learning disability	3.39 (3.21-3.58)	3.76 (3.54-3.99)	2.25 (1.93-2.61)
Severe mental illness	1.96 (1.89-2.03)	2.06 (1.99-2.14)	1.52 (1.39-1.67)
Alcohol problems	1.37 (1.35-1.39)	1.41 (1.38-1.43)	1.25 (1.20-1.30)
Antibiotic count: 1	1.73 (1.71-1.76)	1.70 (1.68-1.73)	1.86 (1.80-1.93)
Antibiotic count: 2-3	2.31 (2.27-2.34)	2.28 (2.25-2.32)	2.40 (2.31-2.49)
Antibiotic count: 3+	3.36 (3.31-3.42)	3.38 (3.32-3.44)	3.35 (3.21-3.50)

Reference groups:
Clinical characteristics: the patients without the clinical disease.
The number of prior antibiotic prescriptions: antibiotic count: 0.
Models were adjusted for all comorbidities*

All comorbidities* Hypertension, chronic cardiac disease, diabetes, stroke, chronic kidney disease or renal replacement therapy) and asthma, cancer (non-haematological and haematological), chronic liver disease, dementia, other neurological disease (including motor neuron disease, myasthenia gravis, multiple sclerosis, Parkinson's disease, cerebral palsy, quadriplegia or hemiplegia, and progressive cerebellar disease), organ kidney transplant, asplenia (due to splenectomy or spleen dysfunction, including sickle cell disease), rheumatoid arthritis/lupus/psoriasis, other immunosuppressive conditions, learning disability, several mental ill, the number of prior antibiotic prescription from one year and six week to six week before the index time (indicating the infection history)

Abbreviations:
CKD, chronic kidney disease; RRT, renal replacement therapy.
The number of prior antibiotic prescriptions (one year plus six weeks to six weeks before the index date)

eTable 19. Adjusted ORs of community-acquired sepsis 30-day mortality stratified by COVID-19 period (Multiple imputation)

Group	Category	OR (Community + Hospital)	OR2 (Community)	OR3 (Hospital)
Age	18-39	0.18(0.15,0.22)	0.19(0.15,0.23)	0.17(0.14,0.20)
Age	40-49	0.59(0.51,0.68)	0.70(0.59,0.81)	0.71(0.62,0.82)
Age	60-69	1.36(1.24,1.48)	1.23(1.10,1.37)	1.21(1.10,1.33)
Age	70-79	1.80(1.66,1.95)	1.52(1.37,1.68)	1.59(1.46,1.72)
Age	80+	3.01(2.79,3.25)	2.44(2.22,2.69)	2.53(2.34,2.74)
Age	<18	0.15(0.11,0.22)	0.10(0.05,0.16)	0.09(0.05,0.13)
Antibiotic count	Antibiotic count: 1	0.96(0.91,1.03)	0.95(0.89,1.02)	0.98(0.93,1.04)
Antibiotic count	Antibiotic count: 2-3	1.00(0.95,1.05)	1.07(1.00,1.14)	1.02(0.97,1.08)
Antibiotic count	Antibiotic count: 3+	1.01(0.97,1.06)	1.05(0.98,1.12)	1.05(0.99,1.10)
Asthma	With no oral steroid use	0.85(0.80,0.90)	0.88(0.81,0.95)	0.87(0.82,0.92)
Asthma	With oral steroid use	0.86(0.79,0.97)	1.09(0.94,1.25)	0.97(0.86,1.09)
BMI	Obese I (30-34.9 kg/m2)	0.83(0.78,0.88)	0.83(0.75,0.91)	0.83(0.77,0.90)
BMI	Obese II (35-39.9 kg/m2)	0.84(0.70,1.00)	0.81(0.72,0.90)	0.89(0.81,0.97)
BMI	Obese III (40+ kg/m2)	0.91(0.82,1.00)	1.04(0.92,1.17)	0.99(0.90,1.09)
BMI	Overweight (25-29.9 kg/m2)	0.90(0.81,0.99)	0.89(0.82,0.98)	0.83(0.76,0.91)
BMI	Underweight (<18.5 kg/m2)	1.10(1.02,1.19)	1.12(1.00,1.25)	1.16(1.06,1.27)
CKD/RRT	CKD stage 3a	0.95(0.91,1.00)	1.02(0.95,1.09)	0.90(0.86,0.96)
CKD/RRT	CKD stage 3b	1.13(1.07,1.19)	1.25(1.16,1.35)	1.12(1.06,1.19)
CKD/RRT	CKD stage 4	1.54(1.43,1.65)	1.59(1.44,1.75)	1.41(1.30,1.53)
CKD/RRT	CKD stage 5	1.67(1.42,1.96)	1.79(1.44,2.22)	1.37(1.15,1.63)
CKD/RRT	RRT (dialysis)	1.24(1.05,1.46)	1.02(0.80,1.30)	0.82(0.65,1.01)
CKD/RRT	RRT (transplant)	0.54(0.39,0.73)	0.58(0.37,0.86)	0.65(0.46,0.89)
Diabetes	Controlled	0.93(0.90,0.97)	0.93(0.88,0.98)	0.93(0.89,0.98)
Diabetes	Not controlled	0.86(0.75,0.98)	0.84(0.69,1.02)	0.81(0.69,0.94)
Diabetes	Without recent Hb1ac measure	0.99(0.89,1.1)	0.99(0.88,1.12)	1.04(0.953,1.14)
Ethnicity	Black	0.73(0.60,0.89)	0.89(0.69,1.13)	0.64(0.51,0.79)
Ethnicity	Mixed	0.61(0.43,0.84)	1.05(0.71,1.52)	0.73(0.53,1.00)
Ethnicity	Other	0.88(0.70,1.09)	1.11(0.83,1.47)	1.06(0.83,1.34)
Ethnicity	South Asian	0.81(0.73,0.90)	0.94(0.80,1.09)	0.86(0.77,0.96)
IMD	IMD1(most deprived)	1.25(1.18,1.32)	1.21(1.12,1.32)	1.21(1.14,1.29)
IMD	IMD2	1.21(1.14,1.29)	1.16(1.07,1.25)	1.08(1.01,1.15)
IMD	IMD3	1.09(1.03,1.16)	1.06(0.98,1.15)	1.04(0.98,1.11)
IMD	IMD4	1.06(1.00,1.13)	1.03(0.95,1.12)	1.02(0.96,1.09)
Others	Alcohol problems	1.35(1.27,1.43)	1.46(1.35,1.57)	1.35(1.27,1.43)
Others	Asplenia	1.36(1.08,1.70)	1.27(0.94,1.70)	0.83(0.62,1.08)
Others	Cancer (non haematological)	1.29(1.24,1.35)	1.26(1.2,1.34)	1.26(1.20,1.31)
Others	Chronic cardiac disease	1.13(1.09,1.18)	1.14(1.08,1.20)	1.14(1.09,1.18)
Others	Chronic liver disease	1.84(1.67,2.02)	2.05(1.81,2.32)	2.04(1.84,2.25)
Others	Chronic respiratory disease	1.19(1.14,1.24)	1.29(1.21,1.37)	1.33(1.27,1.40)
Others	Dementia	1.08(0.99,1.18)	1.14(1.00,1.31)	1.15(1.03,1.29)
Others	Haematological malignancy	0.97(0.88,1.06)	0.94(0.83,1.06)	0.88(0.80,0.98)

Others	Hypertension	0.97(0.93,1.01)	0.98(0.93,1.04)	0.94(0.90,0.98)
Others	Immunosuppressive condition	1.03(0.96,1.11)	0.99(0.90,1.10)	0.98(0.90,1.06)
Others	Learning disability	1.38(1.17,1.61)	1.35(1.07,1.68)	0.98(0.80,1.18)
Others	Other neurological disease	0.99(0.92,1.07)	1.00(0.90,1.11)	1.01(0.93,1.11)
Others	Potential Care Home	0.80(0.76,0.85)	1.21(1.08,1.36)	1.28(1.15,1.41)
Others	Rheumatoid arthritis/ lupus/ psoriasis	1.00(0.94,1.06)	1.02(0.94,1.11)	1.04(0.98,1.11)
Others	Severe mental illness	0.90(0.81,1.01)	1.00(0.85,1.17)	0.92(0.81,1.04)
Others	Stroke	1.03(0.96,1.10)	0.99(0.91,1.08)	0.96(0.91,1.02)
Region	East Midlands	0.95(0.91,1.01)	1.14(1.06,1.22)	1.00(0.94,1.05)
Region	London	0.80(0.72,0.88)	0.98(0.86,1.11)	0.84(0.76,0.94)
Region	North East	1.04(0.95,1.14)	1.15(1.01,1.30)	1.06(0.96,1.17)
Region	North West	1.18(1.10,1.26)	1.27(1.16,1.39)	1.09(1.01,1.17)
Region	South East	0.76(0.70,0.82)	0.86(0.78,0.96)	0.79(0.72,0.86)
Region	South West	0.98(0.92,1.05)	1.04(0.95,1.13)	0.96(0.90,1.03)
Region	West Midlands	1.10(0.99,1.21)	1.37(1.19,1.57)	1.15(1.04,1.26)
Region	Yorkshire and the Humber	1.08(1.02,1.15)	1.23(1.13,1.33)	1.17(1.10,1.25)
Sex	Male	0.97(0.94,1.01)	0.97(0.92,1.02)	0.96(0.92,1.00)
Smoking	Current	1.58(1.49,1.68)	1.61(1.49,1.75)	1.68(1.57,1.79)
Smoking	Former	1.07(1.03,1.11)	1.1(1.04,1.16)	1.15(1.10,1.20)
Tx	Kidney transplant	0.62(0.47,0.79)	0.54(0.37,0.77)	0.59(0.43,0.79)
Tx	Other organ transplant	0.95(0.61,1.43)	0.46(0.19,0.95)	0.84(0.49,1.37)

Models were adjusted for age using a 4-knot cubic spline, except for estimation of age group relative odds of 30-day mortality; and adjusted for sex, except for estimation of sex group relative odds of 30-day mortality; and stratified by region, except for IMD group relative odds of 30-day mortality.
Abbreviations: IMD, index of multiple deprivation; BMI, body mass index;
Tx, transplant; CKD, chronic kidney disease; RRT, renal replacement therapy.

The number of prior antibiotic prescriptions (one year plus six weeks to six weeks before the index date)

Reference sub-group:
age: 50-59 years
sex: female
region: East of England
IMD: the least deprived quintile
ethnicity: white
BMI: healthy range (18.5 – 24.9 kg/m2)
smoking: never
prior antibiotic count: 0
Patients without the disease were used as the reference for other clinical conditions.

eText 1. The selection criteria for potential controls

Every six months, we selected and extracted potential controls. We extracted patients who did not have a diagnosis of sepsis from 15 days prior to the start date up to 15 days after the end date. (The start date and end date were the cut-off dates for each six-month period.)

eText 2. The definition of Index of Multiple Deprivation (IMD) quintile

This variable was determined by accessing the patient's address at the individual level and calculating their ranking relative to the 32,800 Lower Layer Super Output Areas[1]. IMD score incorporates information on various indicators such as income, employment, crime rate, living environment, education, and barriers to services.

eText 3. The definition of predictors

The first set of predictors:
Region, ethnicity (white, mixed, Asian, black, other, unknown), and body mass index (BMI) were all considered in the analysis. To minimize the loss of information resulting from categorizing a continuous variable and missing data, BMI was classified into six groups (plus one group for missing data) based on the NICE definition: underweight (<18.5 kg/m2), healthy range (18.5-24.9 kg/m2), overweight (25-29.9 kg/m2), obese I (30-34.9 kg/m2), II (35-39.9 kg/m2), III (≥40kg/m2)[2,3]. Additionally, smoking status (never, former, current), alcohol problems, and care home status(TPP have matched patient addresses to care homes in the CQC database[4]) were also measured.

The second set of predictors:
The following comorbidities were included: high blood pressure or diagnosed hypertension, chronic respiratory diseases (excluding asthma), asthma (classified based on with or without recent use of oral steroids), chronic heart disease, diabetes (classified based on the most recent HbA1c measurement within the 15 months before the index date), cancer (non-haematological and haematological), chronic kidney disease (CKD) or renal replacement therapy(RRT) (classified based on estimated glomerular filtration rates of ≥60 [absent], <60 and ≥45 [stage 3a],<45 and ≥30 [stage 3b], <30 and ≥15 [stage 4], and <15 [stage 5], respectively, or diagnostic codes indicative of dialysis and kidney transplant), chronic liver disease, stroke, dementia, other neurological disease (including motor neuron disease, myasthenia gravis, multiple sclerosis, Parkinson's disease, cerebral palsy, quadriplegia or hemiplegia, and progressive cerebellar disease), organ transplant (kidney or other), asplenia (due to splenectomy or spleen dysfunction, including sickle cell disease), rheumatoid arthritis/lupus/psoriasis, learning disabilities, severe mental illness, and other immunosuppressive conditions.[5]

The antibiotics included in this were systemic antibiotics for common infections listed in chapter 5.1 (antibacterial drugs) of the British National Formulary (BNF), except for BNF 5.1.9 (antituberculosis drugs) and BNF 5.1.10 (antileprotic drugs). A total of 55 unique antibiotics (based on molecular structure) was prescribed to the study population, referred to as "antibiotic types" to indicate the diversity of antibiotics used. Any antibiotic issued within six weeks before the index date was excluded from the study as they may have been potentially related to treating developing sepsis. The antibiotic exposure was evaluated by total count of antibiotics in the one year before (from one year and six weeks to six weeks before the index date).

eText 4. Missing data

There were missing data for body mass index (BMI), smoking history and ethnicity, which were treated as a separate category in the regression analyses. There was no missing data in comorbidities as they were coded as present or absent.

There were missing data for BMI in 18.5% (N=291,270) in the overall study population of cases and controls, for smoking, 3.0% (N= 47,335) and for ethnicity 5.5 % (N 88,055). With respect to BMI, 28,144 cases and controls had missing BMI as they were patients aged ⩽ 15 years. This occurred because the earliest data extraction for BMI started from 16 years old. In patients aged 16 years or older, the missing data represented 16.8% (N=263,126) of cases and controls for BMI. Regarding smoking, 29,088 of the missing values were from patients aged < 18 years old (with smoking history only infrequently assessed in children in primary care). In patients aged 18 years or older, the missing data represented 1.2% (N=19,375) of cases and controls for smoking history. When examining the missing percentages between cases and controls, we observed that controls typically had more missing records compared to cases. Regarding ethnicity, 2,623 cases and controls had missing ethnicity in patients aged <18 years old. In patients aged 18 years or older, the missing data represent 5.4 % (N = 84,433) of cases and controls for missing ethnicity. These results indicating non-random missingness is not unexpected as typically some risk factor information is only being collected for patients who consult their GP for e.g. health

complaints. The exception is the NHS Health check program which aims to be a population screening of risk factor information involving a check-up in primary care for adults in England aged 40 to 74. It can help spot early signs of stroke, kidney disease, heart disease, type 2 diabetes or dementia[6]. However, a recent study reported that the uptake in health checks is differential, even after adjustment for patient and practice level.[7] For these reasons, it was concluded that data for BMI, ethnicity and smoking history was not missing at random (MNAR) in the study population.

eText 5. Model adjustments for IMD

Sequentially, four models were implemented. The first was an unadjusted model with IMD as the sole predictor (called Unadjusted). In Model 1, the specific COVID-19 time periods were assumed to be a moderator variable and included in model 1 for the analysis of potential heterogeneity of the IMD effects over time due to pandemic. Given the emergent understanding of shared molecular regulatory pathways between sepsis and cardiometabolic conditions—and noting that individuals with such conditions are predisposed to severe manifestations of both COVID-19 and sepsis—Model 2 made adjustments for demographic information from our primary set of predictors (detailed in eText 3) and cardiometabolic comorbidities.[8,9] Lastly, Model 3 accounted for all comorbidities from our secondary set of predictors (refer to eText 3) and also considered the frequency of prior antibiotic prescriptions (spanning the period from one year and six weeks prior to the index date up until six weeks before this date). By following this approach, We aimed to assess whether the association between IMD and the development of non-COVID-19 sepsis remains significant when adjusting for various variables.

eText 6. Comparing the different approaches to dealing with missing data

When using the missing indicator method, the results were as follows: Higher odds of developing non-COVID-19 sepsis were notably associated with the most deprived quintiles (Figure 3) in the all-age population. The crude ORs [95% Confidence Intervals (CI)] were as follows: For IMD1 (most deprived), it was 1.80 [1.77-1.83]; IMD2, 1.48 [1.46-1.51]; IMD3, 1.25 [1.23-1.27]; and IMD4, 1.14 [1.12-1.16]. In the 18+ population, the crude ORs were consistent: 1.81 [1.78-1.84] for IMD1, 1.48 [1.46-1.50] for IMD2, 1.25 [1.23-1.27] for IMD3, and 1.14 [1.12-1.16] for IMD4 (eFigure 6).

Upon the application of multiple imputations, in the all-age cohort, the crude ORs were consistent: 1.80 [1.77-1.83] for IMD1, 1.48 [1.46-1.50] for IMD2, 1.25 [1.23-1.27] for IMD3, and 1.14 [1.12-1.16] for IMD4 (eTable 15). Similarly, in the 18+ age category, the crude ORs after multiple imputations were 1.81 [1.78-1.84] for IMD1, 1.48 [1.46-1.51] for IMD2, 1.25 [1.23-1.27] for IMD3, and 1.14 [1.12-1.16] for IMD4 (eTable 16).

In the competing risk analysis: 1.79 [1.76-1.82] for IMD1, 1.48 [1.45-1.50] for IMD2, 1.24 [1.22-1.26] for IMD3, and 1.14 [1.12-1.16] for IMD4 (eTable 11)."

We also addressed the additional sensitivity result in the result section: "We found the result matched properly in the sensitivity analysis for 18+ study population (eFigure 6-9). The complete case analysis (eTable 9-12) and the analysis using multiple imputation (eTable 15-19) also presented a consistent result (see further statement in eText 6).

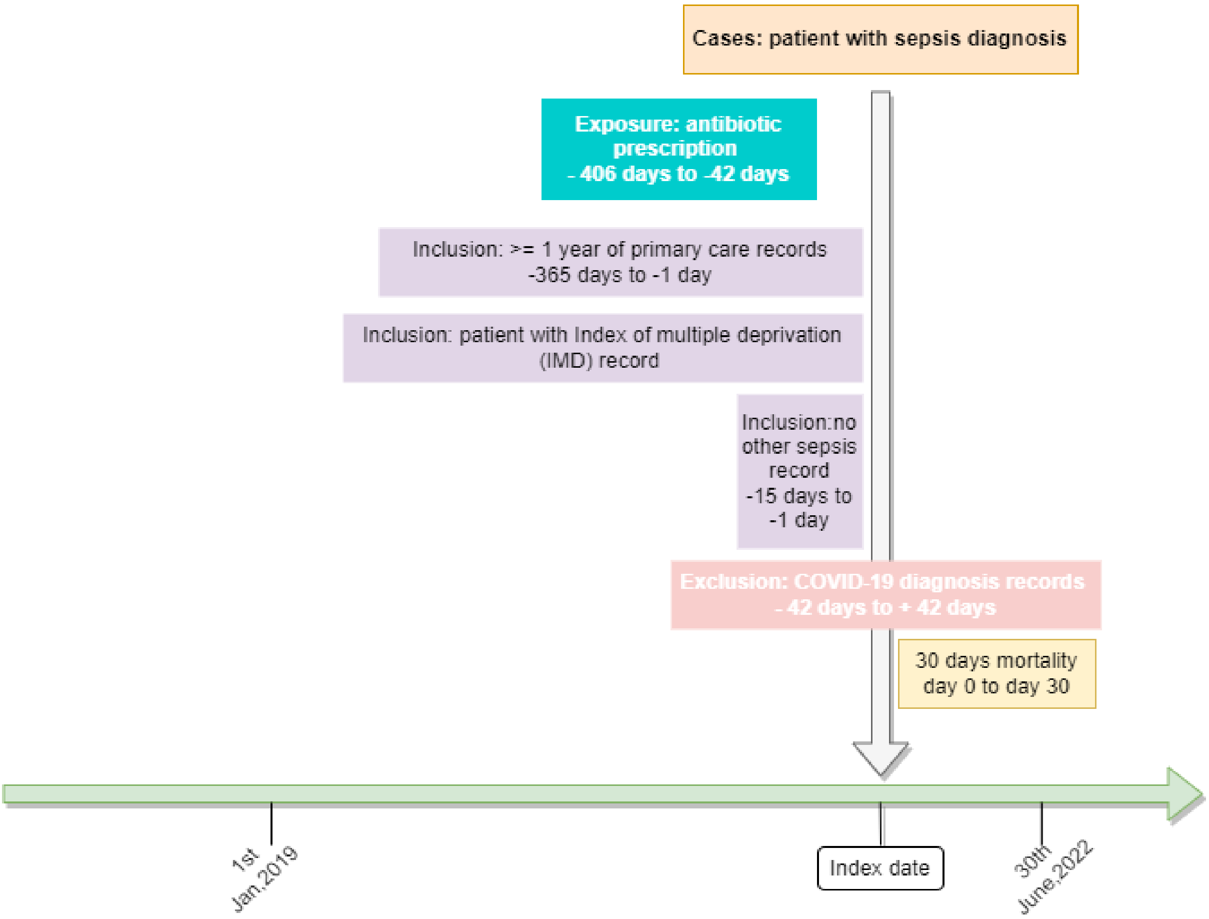

eFigure 1. Diagram of patient selection (index date) and prior antibiotic measurements

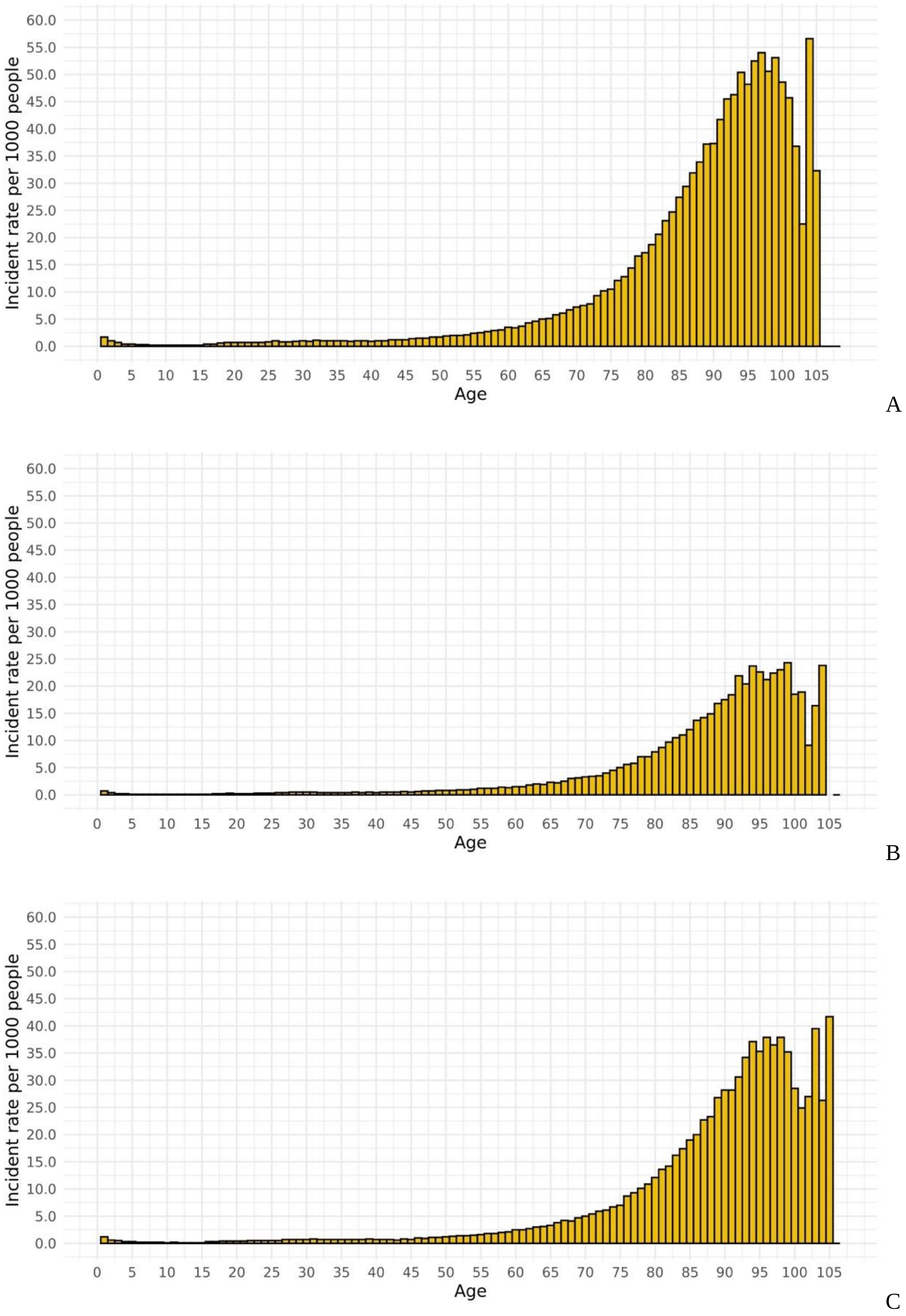

eFigure 2. Age-specific incidence rate of community-acquired sepsis

To reduce the risk of secondary disclosure, all counted numbers in the graph were rounded to the nearest five, the incident rate was calculated after rounded

The incidence rate of sepsis was calculated as the number of cases per 1000 registered persons

(A. Period 1: 2019-01-01 to 2020-03-25; B. Period 2: 2020-03-26 to 2021-03-08; C. Period 3: 2021-03-09 to 2022-06-30)

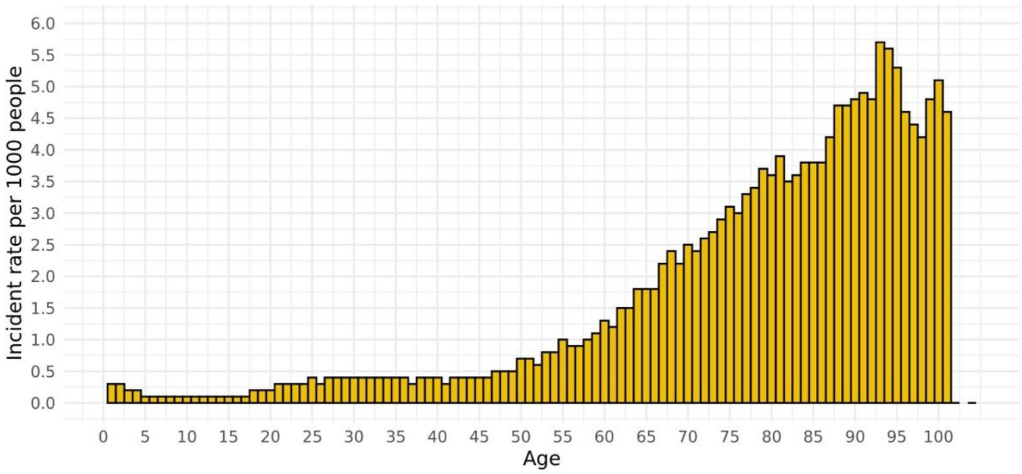

A

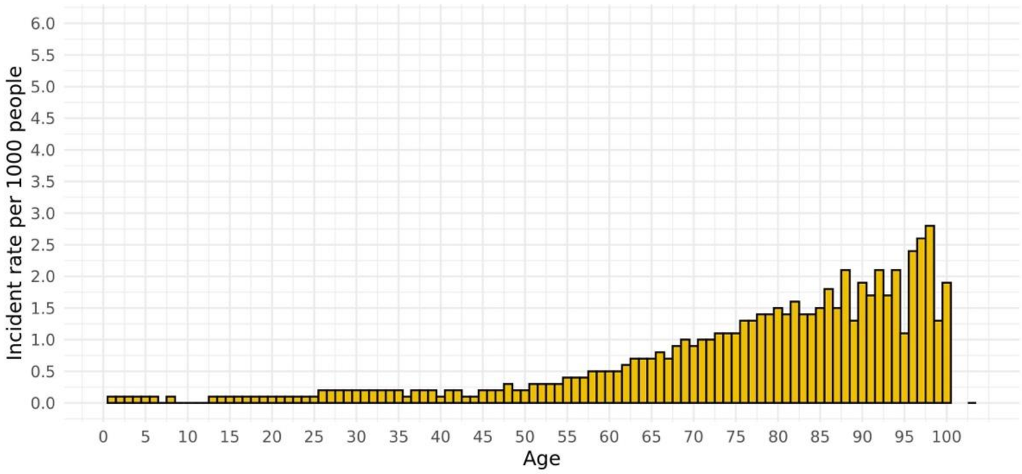

B

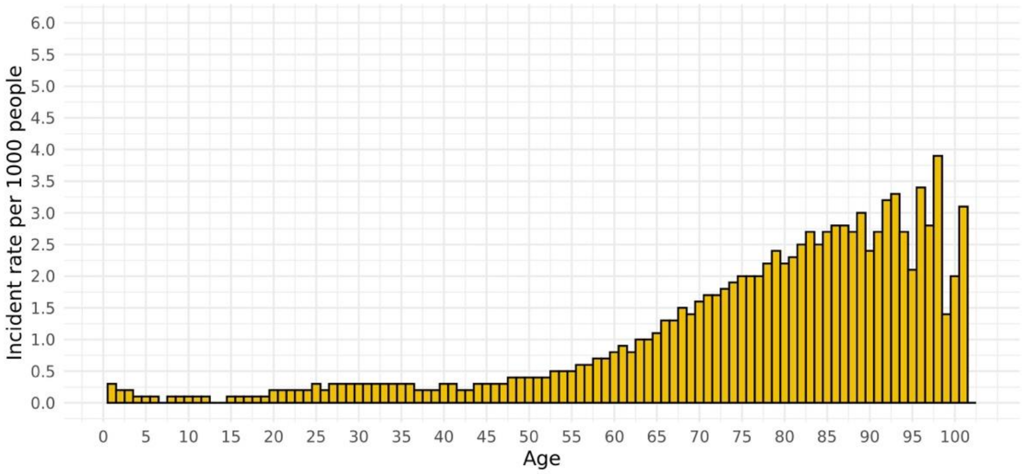

C

eFigure 3. Age-specific incidence rate of hospital-acquired sepsis

To reduce the risk of secondary disclosure, all counted numbers in the graph were rounded to the nearest five, the incident rate was calculated after rounded
The incidence rate of sepsis was calculated as the number of cases per 1000 registered persons
(A. Period 1: 2019-01-01 to 2020-03-25; B. Period 2: 2020-03-26 to 2021-03-08; C. Period 3: 2021-03-09 to 2022-06-30)

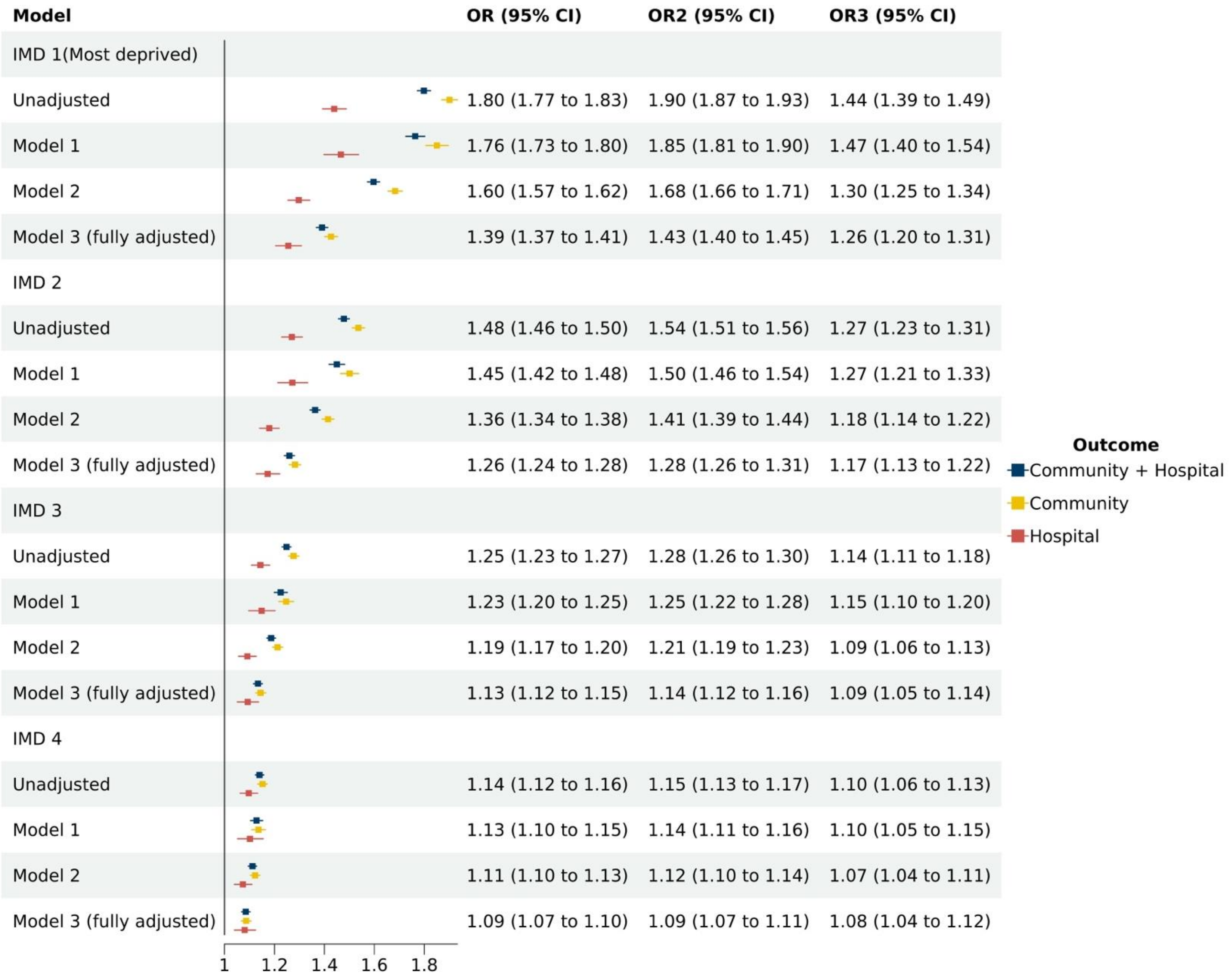

eFigure 4. Association between index of multiple deprivations (IMD) quintile and risk of hospital admission for Sepsis

OR: Community + Hospital, OR2: Community, OR3: Hospital
IMD (Index of Multiple Deprivation) quintile measured from patient-level address.
Odds ratios are compared to the least deprived quintile (IMD 5)
Model 1 – adjusted for interaction term between IMD and time (Period 1: 2019-01-01 to 2020-03-25; Period 2: 2020-03-26 to 2021-03-08; Period 3: 2021-03-09 to 2022-06-30)
Model 2 – adjusted for cardiometabolic comorbidities*
Model 3 (fully adjusted model) – adjust for all comorbidities**

Cardiometabolic comorbidities* Hypertension, chronic cardiac disease, diabetes, stroke, chronic kidney disease or renal replacement therapy)
All comorbidities** Cardiometabolic comorbidities and asthma, cancer (non-haematological and haematological), chronic liver disease, dementia, other neurological disease (including motor neuron disease, myasthenia gravis, multiple sclerosis, Parkinson's disease, cerebral palsy, quadriplegia or hemiplegia, and progressive cerebellar disease), organ kidney transplant, asplenia (due to splenectomy or spleen dysfunction, including sickle cell disease), rheumatoid arthritis/lupus/psoriasis, other immunosuppressive conditions, learning disability, several mental ill, the number of prior antibiotic prescription from one year and six week to six week before the index time (indicating the infection history)

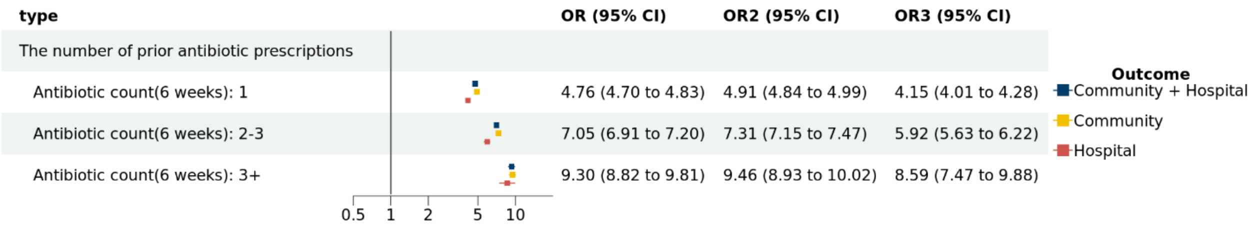

type	OR (95% CI)	OR2 (95% CI)	OR3 (95% CI)
The number of prior antibiotic prescriptions			
Antibiotic count(6 weeks): 1	4.76 (4.70 to 4.83)	4.91 (4.84 to 4.99)	4.15 (4.01 to 4.28)
Antibiotic count(6 weeks): 2-3	7.05 (6.91 to 7.20)	7.31 (7.15 to 7.47)	5.92 (5.63 to 6.22)
Antibiotic count(6 weeks): 3+	9.30 (8.82 to 9.81)	9.46 (8.93 to 10.02)	8.59 (7.47 to 9.88)

A

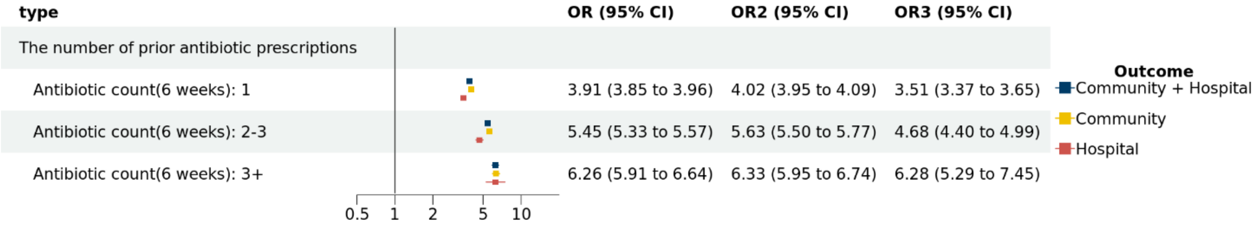

type	OR (95% CI)	OR2 (95% CI)	OR3 (95% CI)
The number of prior antibiotic prescriptions			
Antibiotic count(6 weeks): 1	3.91 (3.85 to 3.96)	4.02 (3.95 to 4.09)	3.51 (3.37 to 3.65)
Antibiotic count(6 weeks): 2-3	5.45 (5.33 to 5.57)	5.63 (5.50 to 5.77)	4.68 (4.40 to 4.99)
Antibiotic count(6 weeks): 3+	6.26 (5.91 to 6.64)	6.33 (5.95 to 6.74)	6.28 (5.29 to 7.45)

B

eFigure 5. ORs for the number of antibiotics given within six weeks before the index date

OR: Community + Hospital, OR2: Community, OR3: Hospital
A. crude ORs
B. adjusted for all comorbidities*

All comorbidities* Hypertension, chronic cardiac disease, diabetes, stroke, chronic kidney disease or renal replacement therapy) and asthma, cancer (non-haematological and haematological), chronic liver disease, dementia, other neurological disease (including motor neuron disease, myasthenia gravis, multiple sclerosis, Parkinson's disease, cerebral palsy, quadriplegia or hemiplegia, and progressive cerebellar disease), organ kidney transplant, asplenia (due to splenectomy or spleen dysfunction, including sickle cell disease), rheumatoid arthritis/lupus/psoriasis, other immunosuppressive conditions, learning disability, several mental ill, the number of prior antibiotic prescription from one year and six week to six week before the index time (indicating the infection history)

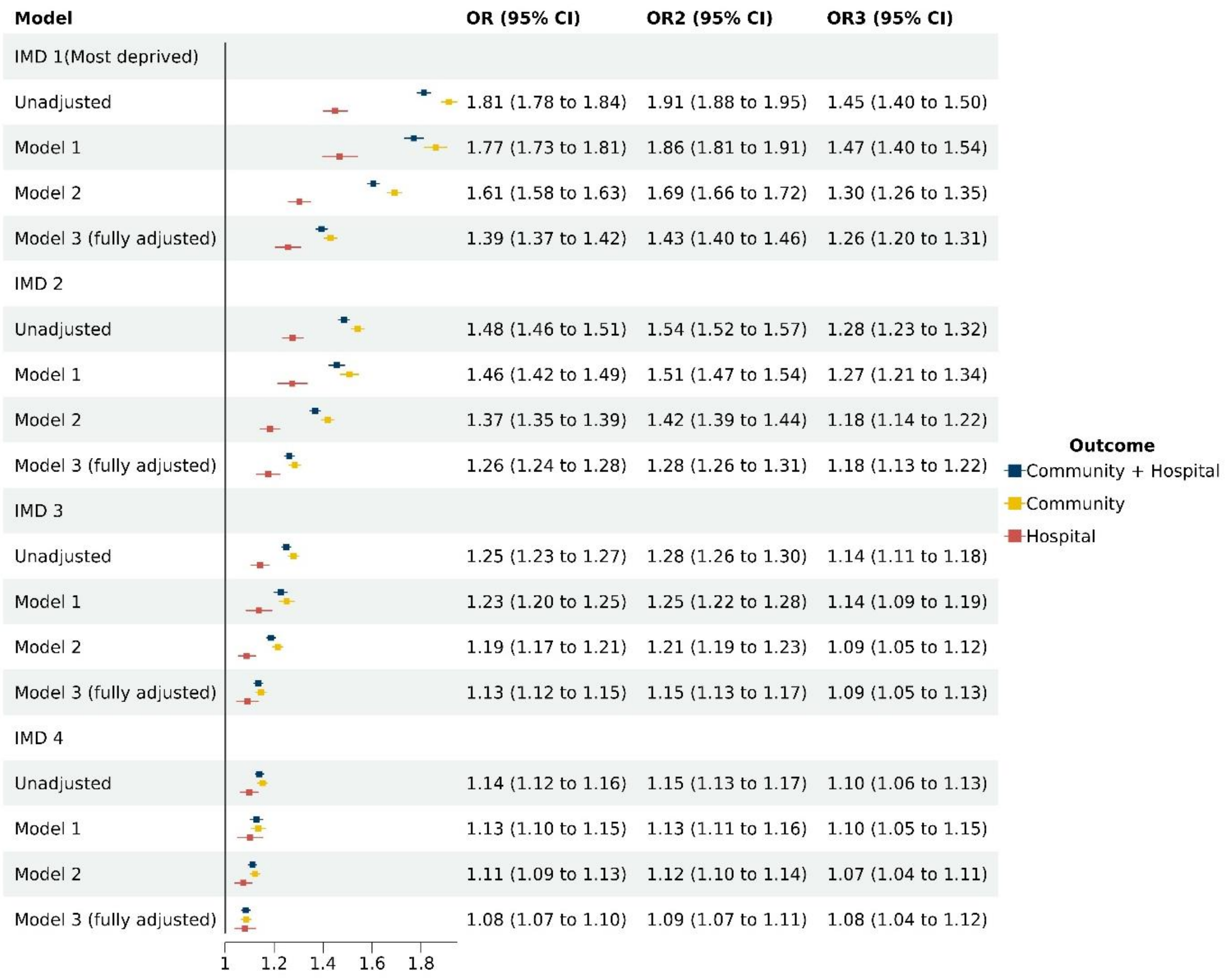

eFigure 6. Association between index of multiple deprivations (IMD) quintile and risk of hospital admission for Sepsis (18+ years old study population)

OR: Community + Hospital, OR2: Community, OR3: Hospital
IMD (Index of Multiple Deprivation) quintile measured from patient-level address.
Odds ratios are compared to the least deprived quintile (IMD 5)
Model 1 – adjusted for interaction term between IMD and time (Period 1: 2019-01-01 to 2020-03-25; Period 2: 2020-03-26 to 2021-03-08; Period 3: 2021-03-09 to 2022-06-30)
Model 2 – adjusted for cardiometabolic comorbidities*
Model 3 (fully adjusted model) – adjust for all comorbidities**

Cardiometabolic comorbidities* Hypertension, chronic cardiac disease, diabetes, stroke, chronic kidney disease or renal replacement therapy)
All comorbidities** Cardiometabolic comorbidities and asthma, cancer (non-haematological and haematological), chronic liver disease, dementia, other neurological disease (including motor neuron disease, myasthenia gravis, multiple sclerosis, Parkinson's disease, cerebral palsy, quadriplegia or hemiplegia, and progressive cerebellar disease), organ kidney transplant, asplenia (due to splenectomy or spleen dysfunction, including sickle cell disease), rheumatoid arthritis/lupus/psoriasis, other immunosuppressive conditions, learning disability, several mental ill, the number of prior antibiotic prescription from one year and six week to six week before the index time (indicating the infection history)

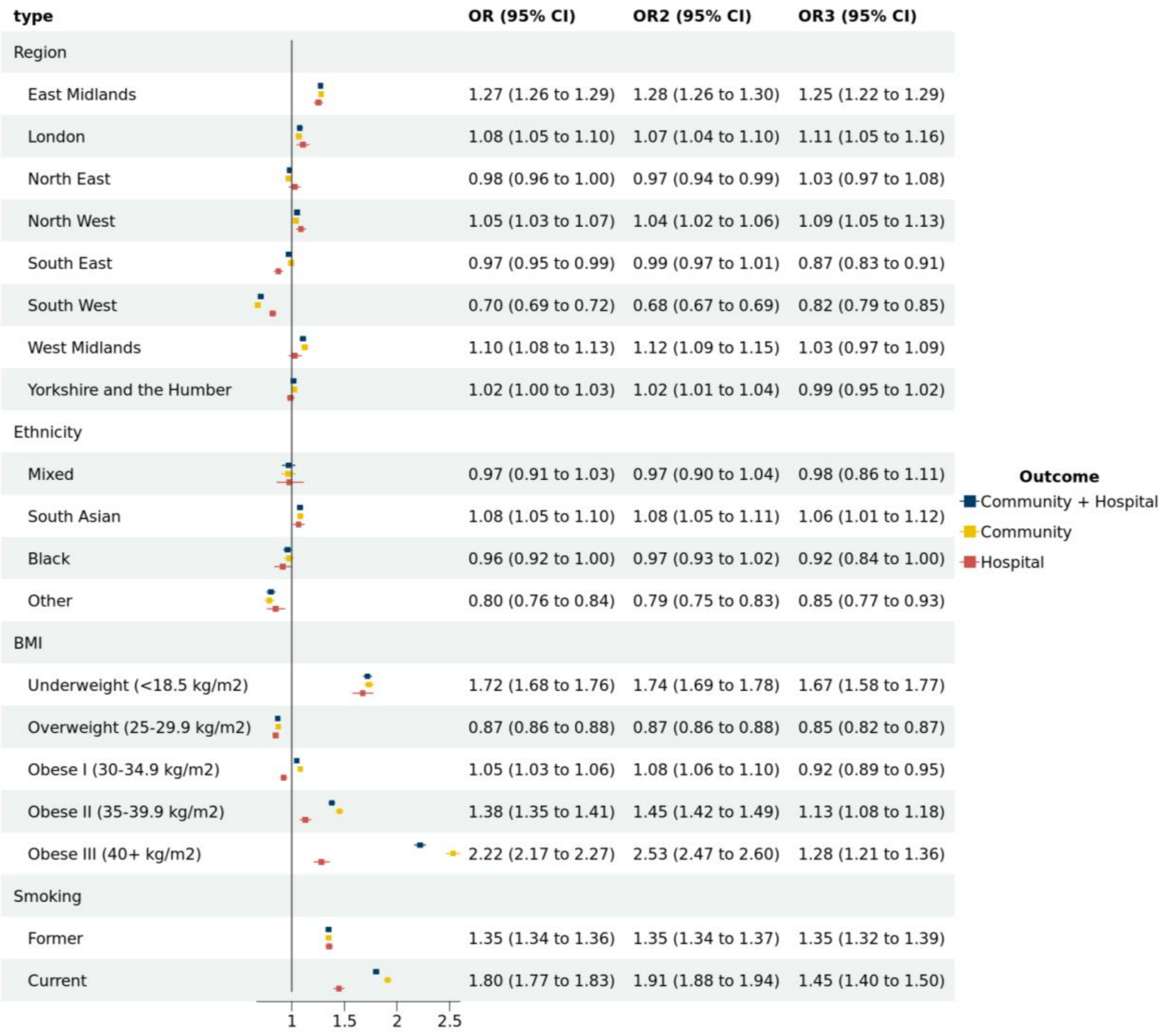

type	OR (95% CI)	OR2 (95% CI)	OR3 (95% CI)
Region			
East Midlands	1.27 (1.26 to 1.29)	1.28 (1.26 to 1.30)	1.25 (1.22 to 1.29)
London	1.08 (1.05 to 1.10)	1.07 (1.04 to 1.10)	1.11 (1.05 to 1.16)
North East	0.98 (0.96 to 1.00)	0.97 (0.94 to 0.99)	1.03 (0.97 to 1.08)
North West	1.05 (1.03 to 1.07)	1.04 (1.02 to 1.06)	1.09 (1.05 to 1.13)
South East	0.97 (0.95 to 0.99)	0.99 (0.97 to 1.01)	0.87 (0.83 to 0.91)
South West	0.70 (0.69 to 0.72)	0.68 (0.67 to 0.69)	0.82 (0.79 to 0.85)
West Midlands	1.10 (1.08 to 1.13)	1.12 (1.09 to 1.15)	1.03 (0.97 to 1.09)
Yorkshire and the Humber	1.02 (1.00 to 1.03)	1.02 (1.01 to 1.04)	0.99 (0.95 to 1.02)
Ethnicity			
Mixed	0.97 (0.91 to 1.03)	0.97 (0.90 to 1.04)	0.98 (0.86 to 1.11)
South Asian	1.08 (1.05 to 1.10)	1.08 (1.05 to 1.11)	1.06 (1.01 to 1.12)
Black	0.96 (0.92 to 1.00)	0.97 (0.93 to 1.02)	0.92 (0.84 to 1.00)
Other	0.80 (0.76 to 0.84)	0.79 (0.75 to 0.83)	0.85 (0.77 to 0.93)
BMI			
Underweight (<18.5 kg/m2)	1.72 (1.68 to 1.76)	1.74 (1.69 to 1.78)	1.67 (1.58 to 1.77)
Overweight (25-29.9 kg/m2)	0.87 (0.86 to 0.88)	0.87 (0.86 to 0.88)	0.85 (0.82 to 0.87)
Obese I (30-34.9 kg/m2)	1.05 (1.03 to 1.06)	1.08 (1.06 to 1.10)	0.92 (0.89 to 0.95)
Obese II (35-39.9 kg/m2)	1.38 (1.35 to 1.41)	1.45 (1.42 to 1.49)	1.13 (1.08 to 1.18)
Obese III (40+ kg/m2)	2.22 (2.17 to 2.27)	2.53 (2.47 to 2.60)	1.28 (1.21 to 1.36)
Smoking			
Former	1.35 (1.34 to 1.36)	1.35 (1.34 to 1.37)	1.35 (1.32 to 1.39)
Current	1.80 (1.77 to 1.83)	1.91 (1.88 to 1.94)	1.45 (1.40 to 1.50)

eFigure 7. Crude ORs of sepsis by region, ethnicity, BMI, smoking history and stratified by type of sepsis (18+ years old study population)

OR: Community + Hospital, OR2: Community, OR3: Hospital
BMI, Body Mass Index (from the most recent clinical records)
Models were unadjusted crude model.
Reference groups:
Region: East of England
Ethnicity: white
BMI: healthy range (18.5 – 24.9 kg/m2)
Smoking: never (Smoking status identified from the most recent clinical records)

type	OR (95% CI)	OR2 (95% CI)	OR3 (95% CI)
Care homes status			
Potential Care Home	3.18 (3.11 to 3.26)	3.27 (3.20 to 3.35)	2.54 (2.37 to 2.72)
Chronic kidney disease or renal replacement therapy			
CKD stage 3a	1.50 (1.47 to 1.52)	1.47 (1.45 to 1.49)	1.63 (1.57 to 1.69)
CKD stage 3b	2.26 (2.22 to 2.30)	2.21 (2.17 to 2.25)	2.53 (2.42 to 2.65)
CKD stage 4	3.89 (3.80 to 3.99)	3.80 (3.70 to 3.91)	4.46 (4.16 to 4.77)
CKD stage 5	8.87 (8.33 to 9.44)	8.34 (7.78 to 8.93)	11.93 (10.24 to 13.91)
RRT (dialysis)	10.95 (10.32 to 11.63)	9.57 (8.94 to 10.25)	17.33 (15.22 to 19.75)
RRT (transplant)	12.14 (11.13 to 13.25)	12.58 (11.40 to 13.87)	10.73 (8.89 to 12.95)
Asthma			
With no oral steroid use	1.15 (1.14 to 1.17)	1.16 (1.15 to 1.18)	1.12 (1.08 to 1.15)
With oral steroid use	1.76 (1.71 to 1.81)	1.78 (1.72 to 1.84)	1.70 (1.60 to 1.81)
Diabetes			
Controlled	1.92 (1.89 to 1.94)	1.97 (1.94 to 1.99)	1.70 (1.66 to 1.75)
Not controlled	3.19 (3.07 to 3.31)	3.37 (3.23 to 3.52)	2.45 (2.23 to 2.69)
Without recent Hb1ac measure	1.90 (1.85 to 1.95)	1.94 (1.89 to 2.00)	1.70 (1.59 to 1.80)
Organ transplant			
Kidney transplant	12.32 (11.39 to 13.32)	12.66 (11.58 to 13.84)	11.16 (9.46 to 13.18)
Other organ transplant	8.00 (7.00 to 9.13)	7.87 (6.77 to 9.15)	8.44 (6.40 to 11.13)
Other clinical characteristics			
Hypertension	1.51 (1.49 to 1.52)	1.54 (1.52 to 1.56)	1.39 (1.36 to 1.42)
Chronic respiratory disease	2.18 (2.15 to 2.21)	2.20 (2.17 to 2.23)	2.07 (2.01 to 2.14)
Chronic cardiac disease	1.96 (1.94 to 1.98)	1.96 (1.93 to 1.98)	1.99 (1.93 to 2.04)
Cancer (non haematological)	2.43 (2.40 to 2.45)	1.83 (1.81 to 1.86)	6.51 (6.35 to 6.67)
Haematological malignancy	5.07 (4.95 to 5.19)	3.11 (3.02 to 3.20)	15.55 (14.84 to 16.30)
Chronic liver disease	4.68 (4.53 to 4.82)	4.72 (4.56 to 4.88)	4.51 (4.20 to 4.84)
Stroke	2.00 (1.97 to 2.03)	2.05 (2.02 to 2.08)	1.77 (1.70 to 1.84)
Dementia	2.29 (2.22 to 2.37)	2.41 (2.34 to 2.50)	1.49 (1.34 to 1.65)
Other neurological disease	2.91 (2.84 to 2.98)	3.11 (3.03 to 3.19)	2.00 (1.88 to 2.13)
Asplenia	2.93 (2.75 to 3.12)	2.66 (2.48 to 2.86)	4.13 (3.61 to 4.71)
Rheumatoid arthritis/ lupus/ psoriasis	1.45 (1.43 to 1.47)	1.46 (1.44 to 1.49)	1.41 (1.36 to 1.46)
Immunosuppressive condition	4.14 (4.07 to 4.22)	2.79 (2.73 to 2.85)	11.63 (11.20 to 12.07)
Learning disability	4.74 (4.51 to 4.98)	5.50 (5.22 to 5.81)	2.34 (2.07 to 2.65)
Severe mental illness	2.56 (2.48 to 2.64)	2.79 (2.70 to 2.89)	1.68 (1.55 to 1.81)
Alcohol problems	1.62 (1.60 to 1.65)	1.68 (1.65 to 1.71)	1.41 (1.36 to 1.46)
The number of prior antibiotic prescriptions			
Antibiotic count: 1	2.08 (2.05 to 2.11)	2.02 (2.00 to 2.05)	2.30 (2.24 to 2.37)
Antibiotic count: 2-3	3.06 (3.02 to 3.10)	3.00 (2.96 to 3.05)	3.30 (3.20 to 3.41)
Antibiotic count: 3+	5.14 (5.07 to 5.21)	5.12 (5.04 to 5.20)	5.19 (5.02 to 5.37)

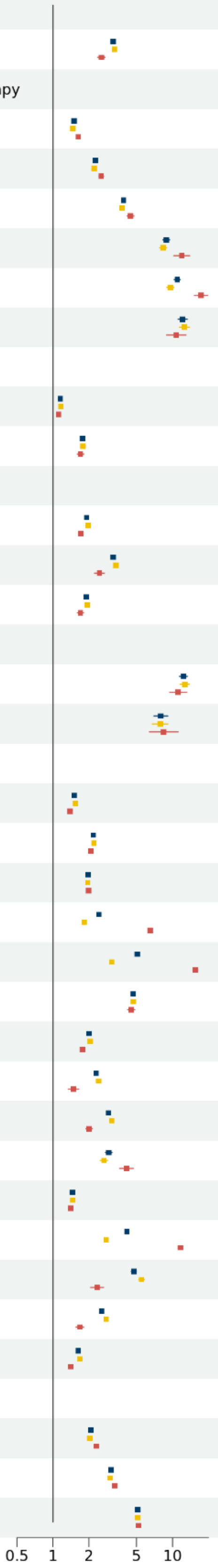

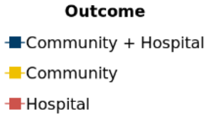

eFigure 8. Crude ORs of developing sepsis for clinical characteristics stratified by sepsis type (18+ years old study population)

OR: Community + Hospital, OR2: Community, OR3: Hospital
Reference groups:
Clinical characteristics: the patients without the clinical disease.
The number of prior antibiotic prescriptions: antibiotic count: 0.
Models were unadjusted

All comorbidities* Hypertension, chronic cardiac disease, diabetes, stroke, chronic kidney disease or renal replacement therapy) and asthma, cancer (non-haematological and haematological), chronic liver disease, dementia, other neurological disease (including motor neuron disease, myasthenia gravis, multiple sclerosis, Parkinson's disease, cerebral palsy, quadriplegia or hemiplegia, and progressive cerebellar disease), organ kidney transplant, asplenia (due to splenectomy or spleen dysfunction, including sickle cell disease), rheumatoid arthritis/lupus/psoriasis, other immunosuppressive conditions, learning disability, several mental ill, the number of prior antibiotic prescription from one year and six week to six week before the index time (indicating the infection history)

Abbreviations: CKD, chronic kidney disease; RRT, renal replacement therapy.
The number of prior antibiotic prescriptions (one year plus six weeks to six weeks before the index date)

type	OR (95% CI)	OR2 (95% CI)	OR3 (95% CI)
Care homes status			
Potential Care Home	2.34 (2.28 to 2.40)	2.32 (2.26 to 2.38)	2.30 (2.11 to 2.50)
Chronic kidney disease or renal replacement therapy			
CKD stage 3a	1.24 (1.23 to 1.26)	1.24 (1.22 to 1.26)	1.27 (1.22 to 1.33)
CKD stage 3b	1.70 (1.67 to 1.73)	1.68 (1.65 to 1.72)	1.82 (1.72 to 1.92)
CKD stage 4	2.62 (2.55 to 2.70)	2.58 (2.51 to 2.66)	3.10 (2.85 to 3.38)
CKD stage 5	6.23 (5.81 to 6.68)	5.79 (5.37 to 6.24)	11.15 (9.23 to 13.47)
RRT (dialysis)	7.22 (6.73 to 7.75)	6.13 (5.66 to 6.64)	17.71 (14.97 to 20.94)
RRT (transplant)	5.39 (4.27 to 6.80)	4.80 (3.68 to 6.25)	10.29 (6.15 to 17.21)
Asthma			
With no oral steroid use	0.99 (0.98 to 1.01)	1.00 (0.98 to 1.01)	1.00 (0.96 to 1.04)
With oral steroid use	0.91 (0.89 to 0.94)	0.92 (0.89 to 0.96)	0.88 (0.81 to 0.95)
Diabetes			
Controlled	1.45 (1.43 to 1.47)	1.47 (1.45 to 1.49)	1.35 (1.30 to 1.39)
Not controlled	2.39 (2.28 to 2.49)	2.47 (2.36 to 2.59)	2.07 (1.84 to 2.33)
Without recent Hb1ac measure	1.58 (1.53 to 1.62)	1.61 (1.56 to 1.66)	1.38 (1.28 to 1.49)
Organ transplant			
Kidney transplant	1.49 (1.21 to 1.85)	1.74 (1.37 to 2.22)	0.79 (0.50 to 1.25)
Other organ transplant	1.55 (1.33 to 1.81)	1.62 (1.36 to 1.92)	1.44 (1.02 to 2.04)
Other clinical characteristics			
Hypertension	1.12 (1.11 to 1.14)	1.14 (1.13 to 1.16)	1.06 (1.02 to 1.09)
Chronic respiratory disease	1.44 (1.41 to 1.46)	1.45 (1.43 to 1.47)	1.41 (1.36 to 1.47)
Chronic cardiac disease	1.38 (1.37 to 1.40)	1.37 (1.35 to 1.38)	1.55 (1.50 to 1.60)
Cancer (non haematological)	2.35 (2.32 to 2.38)	1.73 (1.71 to 1.76)	7.73 (7.51 to 7.96)
Haematological malignancy	2.47 (2.37 to 2.57)	1.79 (1.70 to 1.88)	4.58 (4.19 to 5.00)
Chronic liver disease	3.06 (2.95 to 3.17)	3.07 (2.95 to 3.19)	3.38 (3.08 to 3.71)
Stroke	1.47 (1.45 to 1.50)	1.48 (1.46 to 1.51)	1.43 (1.36 to 1.50)
Dementia	1.41 (1.36 to 1.47)	1.44 (1.39 to 1.49)	1.10 (0.97 to 1.25)
Other neurological disease	2.30 (2.24 to 2.36)	2.41 (2.35 to 2.48)	1.86 (1.72 to 2.01)
Asplenia	1.11 (1.03 to 1.19)	1.12 (1.03 to 1.21)	1.27 (1.07 to 1.51)
Rheumatoid arthritis/ lupus/ psoriasis	1.25 (1.23 to 1.27)	1.26 (1.24 to 1.28)	1.22 (1.17 to 1.28)
Immunosuppressive condition	2.05 (1.98 to 2.12)	1.61 (1.55 to 1.67)	5.01 (4.66 to 5.39)
Learning disability	3.39 (3.21 to 3.58)	3.76 (3.54 to 3.99)	2.25 (1.93 to 2.61)
Severe mental illness	1.96 (1.89 to 2.03)	2.06 (1.99 to 2.14)	1.52 (1.39 to 1.67)
Alcohol problems	1.37 (1.35 to 1.39)	1.41 (1.38 to 1.43)	1.25 (1.20 to 1.30)
The number of prior antibiotic prescriptions			
Antibiotic count: 1	1.73 (1.71 to 1.76)	1.70 (1.68 to 1.73)	1.86 (1.80 to 1.93)
Antibiotic count: 2-3	2.31 (2.27 to 2.34)	2.28 (2.25 to 2.32)	2.40 (2.31 to 2.49)
Antibiotic count: 3+	3.36 (3.31 to 3.42)	3.38 (3.32 to 3.44)	3.35 (3.21 to 3.50)

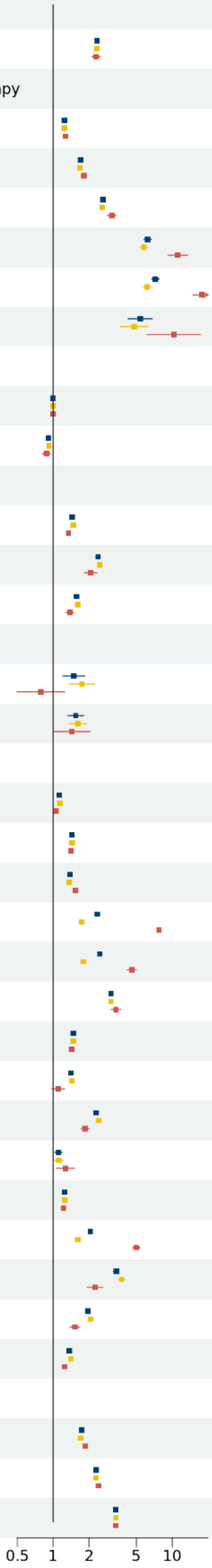

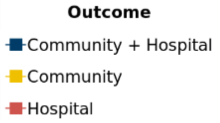

eFigure 9. Adjusted ORs of developing sepsis for clinical characteristics stratified by sepsis type (18+ years old study population)

OR: Community + Hospital, OR2: Community, OR3: Hospital
Reference groups:
Clinical characteristics: the patients without the clinical disease.
The number of prior antibiotic prescriptions: antibiotic count: 0.
Models were adjusted for all comorbidities*

All comorbidities* Hypertension, chronic cardiac disease, diabetes, stroke, chronic kidney disease or renal replacement therapy) and asthma, cancer (non-haematological and haematological), chronic liver disease, dementia, other neurological disease (including motor neuron disease, myasthenia gravis, multiple sclerosis, Parkinson's disease, cerebral palsy, quadriplegia or hemiplegia, and progressive cerebellar disease), organ kidney transplant, asplenia (due to splenectomy or spleen dysfunction, including sickle cell disease), rheumatoid arthritis/lupus/psoriasis, other immunosuppressive conditions, learning disability, several mental ill, the number of prior antibiotic prescription from one year and six week to six week before the index time (indicating the infection history)

Abbreviations: CKD, chronic kidney disease; RRT, renal replacement therapy.
The number of prior antibiotic prescriptions (one year plus six weeks to six weeks before the index date)

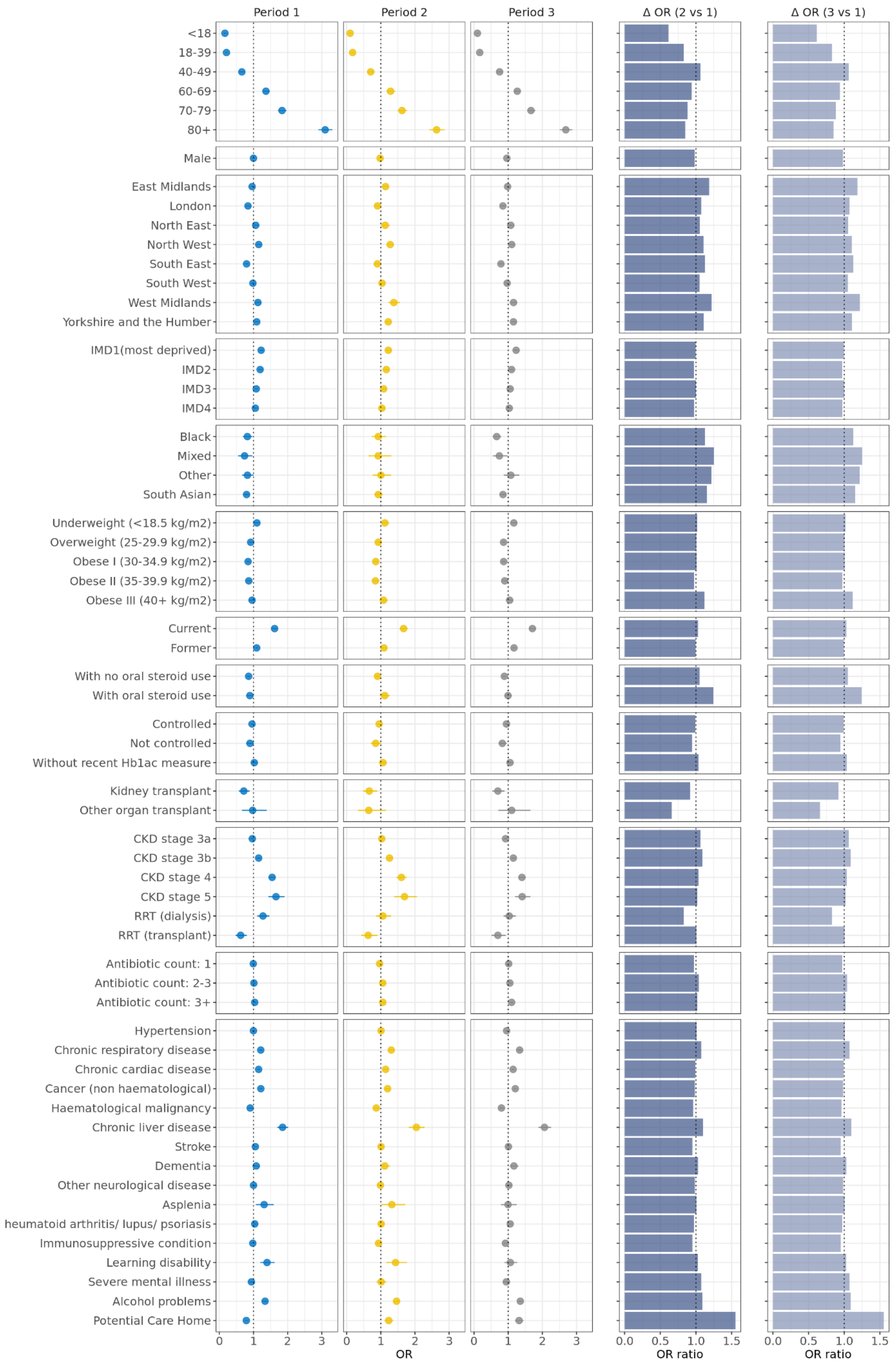

Period 1
Period 2
Period 3
Δ OR (2 vs 1)
Δ OR (3 vs 1)
<18
18-39
40-49
60-69
70-79
80+
Male
East Midlands
London
North East
North West
South East
South West
West Midlands
Yorkshire and the Humber
IMD1(most deprived)
IMD2
IMD3
IMD4
Black
Mixed
Other
South Asian
Underweight (<18.5 kg/m2)
Overweight (25-29.9 kg/m2)
Obese I (30-34.9 kg/m2)
Obese II (35-39.9 kg/m2)
Obese III (40+ kg/m2)
Current
Former
With no oral steroid use
With oral steroid use
Controlled
Not controlled
Without recent Hb1ac measure
Kidney transplant
Other organ transplant
CKD stage 3a
CKD stage 3b
CKD stage 4
CKD stage 5
RRT (dialysis)
RRT (transplant)
Antibiotic count: 1
Antibiotic count: 2-3
Antibiotic count: 3+
Hypertension
Chronic respiratory disease
Chronic cardiac disease
Cancer (non haematological)
Haematological malignancy
Chronic liver disease
Stroke
Dementia
Other neurological disease
Asplenia
heumatoid arthritis/ lupus/ psoriasis
Immunosuppressive condition
Learning disability
Severe mental illness
Alcohol problems
Potential Care Home
0 1 2 3
OR
0.0 0.5 1.0 1.5
OR ratio

eFigure 10. Adjusted ORs of all sepsis mortality stratified by COVID-19 period

Relative odds ratio of 30-day mortality after sepsis and 95% confidence intervals in OpenSAFELY-TPP in the three periods (Period 1: 2019-01-01 to 2020-03-25; Period 2: 2020-03-26 to 2021-03-08; Period 3: 2021-03-09 to 2022-06-30).

Models were adjusted for age using a 4-knot cubic spline, except for estimation of age group relative odds of 30-day mortality; and adjusted for sex, except for estimation of sex group relative odds of 30-day mortality; and stratified by region, except for IMD group relative odds of 30-day mortality.

The two columns on the right present the ratio of the relative odds of 30-day mortality (fold-change: Δ OR) of period 2 vs 1 and period 3 vs 1.

Abbreviations: IMD, index of multiple deprivation; BMI, body mass index;
Tx, transplant; CKD, chronic kidney disease; RRT, renal replacement therapy.

The relative odds of 30-day mortality presented in this figure can be found in table SX of the Supplementary material.

The number of prior antibiotic prescriptions (one year plus six weeks to six weeks before the index date)

Reference sub-group:
age: 50-59 years
sex: female
region: East of England
IMD: the least deprived quintile
ethnicity: white
BMI: healthy range (18.5 – 24.9 kg/m2)
smoking: never
prior antibiotic count: 0
Patients without the disease were used as the reference for other clinical conditions.

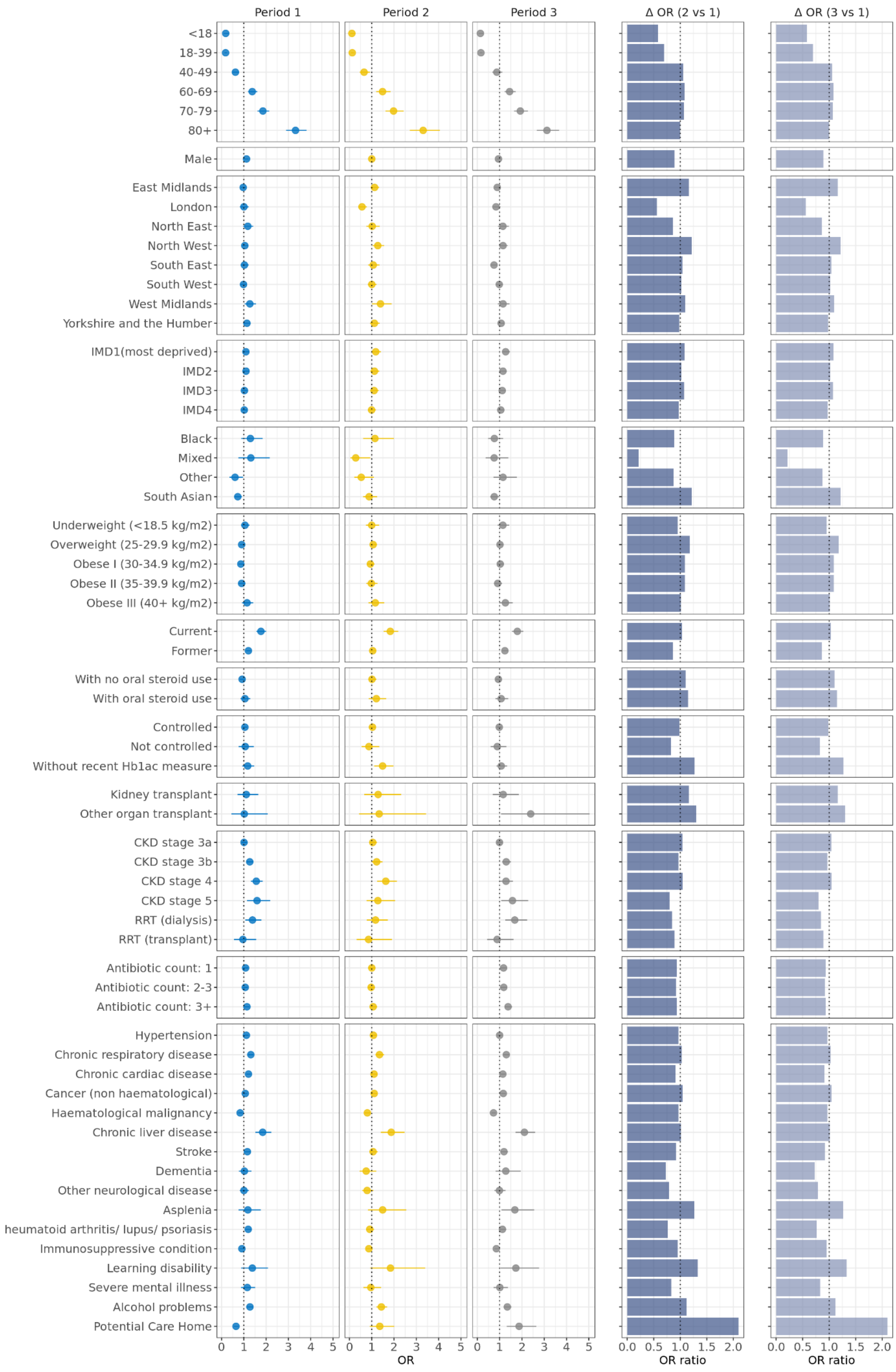

Period 1
Period 2
Period 3
Δ OR (2 vs 1)
Δ OR (3 vs 1)
<18
18-39
40-49
60-69
70-79
80+
Male
East Midlands
London
North East
North West
South East
South West
West Midlands
Yorkshire and the Humber
IMD1(most deprived)
IMD2
IMD3
IMD4
Black
Mixed
Other
South Asian
Underweight (<18.5 kg/m2)
Overweight (25-29.9 kg/m2)
Obese I (30-34.9 kg/m2)
Obese II (35-39.9 kg/m2)
Obese III (40+ kg/m2)
Current
Former
With no oral steroid use
With oral steroid use
Controlled
Not controlled
Without recent Hb1ac measure
Kidney transplant
Other organ transplant
CKD stage 3a
CKD stage 3b
CKD stage 4
CKD stage 5
RRT (dialysis)
RRT (transplant)
Antibiotic count: 1
Antibiotic count: 2-3
Antibiotic count: 3+
Hypertension
Chronic respiratory disease
Chronic cardiac disease
Cancer (non haematological)
Haematological malignancy
Chronic liver disease
Stroke
Dementia
Other neurological disease
Asplenia
heumatoid arthritis/ lupus/ psoriasis
Immunosuppressive condition
Learning disability
Severe mental illness
Alcohol problems
Potential Care Home
0 1 2 3 4 5
OR
0.0 0.5 1.0 1.5 2.0
OR ratio

eFigure 11. Adjusted ORs of hospital-acquired sepsis mortality stratified by COVID-19 period

Relative odds ratio of 30-day mortality after sepsis and 95% confidence intervals in OpenSAFELY-TPP in the three periods (Period 1: 2019-01-01 to 2020-03-25; Period 2: 2020-03-26 to 2021-03-08; Period 3: 2021-03-09 to 2022-06-30).

Models were adjusted for age using a 4-knot cubic spline, except for estimation of age group relative odds of 30-day mortality; and adjusted for sex, except for estimation of sex group relative odds of 30-day mortality; and stratified by region, except for IMD group relative odds of 30-day mortality.

The two columns on the right present the ratio of the relative odds of 30-day mortality (fold-change: Δ OR) of period 2 vs 1 and period 3 vs 1.

Abbreviations: IMD, index of multiple deprivation; BMI, body mass index;
Tx, transplant; CKD, chronic kidney disease; RRT, renal replacement therapy.

The number of prior antibiotic prescriptions (one year plus six weeks to six weeks before the index date)

Reference sub-group:
age: 50-59 years
sex: female
region: East of England
IMD: the least deprived quintile
ethnicity: white
BMI: healthy range (18.5 – 24.9 kg/m2)
smoking: never
prior antibiotic count: 0
Patients without the disease were used as the reference for other clinical conditions.

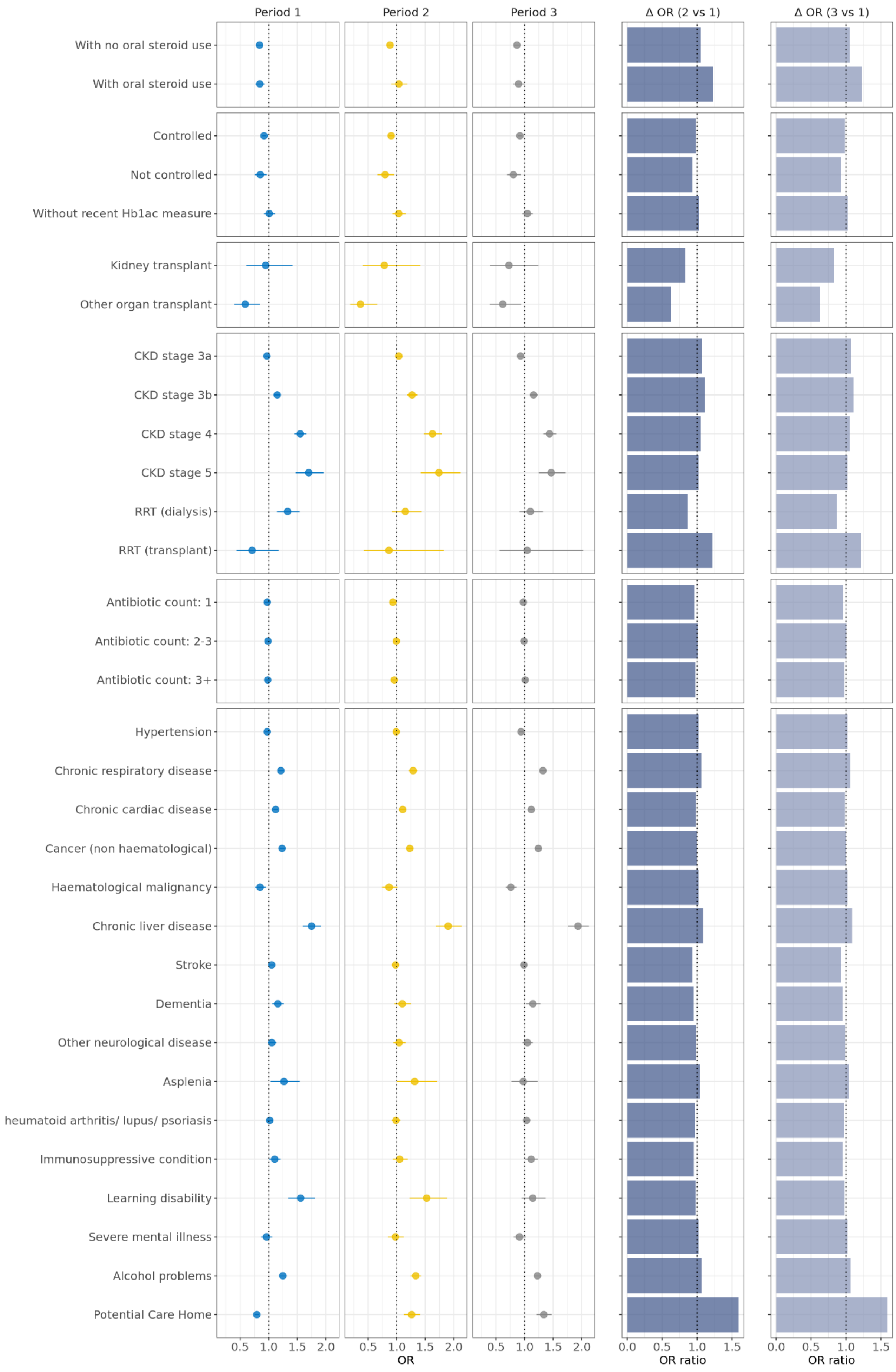
Period 1
Period 2
Period 3
Δ OR (2 vs 1)
Δ OR (3 vs 1)
With no oral steroid use
With oral steroid use
Controlled
Not controlled
Without recent Hb1ac measure
Kidney transplant
Other organ transplant
CKD stage 3a
CKD stage 3b
CKD stage 4
CKD stage 5
RRT (dialysis)
RRT (transplant)
Antibiotic count: 1
Antibiotic count: 2-3
Antibiotic count: 3+
Hypertension
Chronic respiratory disease
Chronic cardiac disease
Cancer (non haematological)
Haematological malignancy
Chronic liver disease
Stroke
Dementia
Other neurological disease
Asplenia
heumatoid arthritis/ lupus/ psoriasis
Immunosuppressive condition
Learning disability
Severe mental illness
Alcohol problems
Potential Care Home
0.5 1.0 1.5 2.0
OR
0.0 0.5 1.0 1.5
OR ratio

eFigure 12. Fully adjusted ORs of all sepsis mortality stratified by COVID-19 period

Relative odds ratio of 30-day mortality after sepsis and 95% confidence intervals in OpenSAFELY-TPP in the three periods (Period 1: 2019-01-01 to 2020-03-25; Period 2: 2020-03-26 to 2021-03-08; Period 3: 2021-03-09 to 2022-06-30).

Models were adjusted for age using a 4-knot cubic spline, except for estimation of age group relative odds of 30-day mortality; and adjusted for sex, except for estimation of sex group relative odds of 30-day mortality; and stratified by region, except for IMD group relative odds of 30-day mortality.

The two columns on the right present the ratio of the relative odds of 30-day mortality (fold-change: Δ OR) of period 2 vs 1 and period 3 vs 1.

Abbreviations: CKD, chronic kidney disease; RRT, renal replacement therapy.

The number of prior antibiotic prescriptions (one year plus six weeks to six weeks before the index date)

Reference sub-group:
Patients without the disease were used as the reference for other clinical conditions.

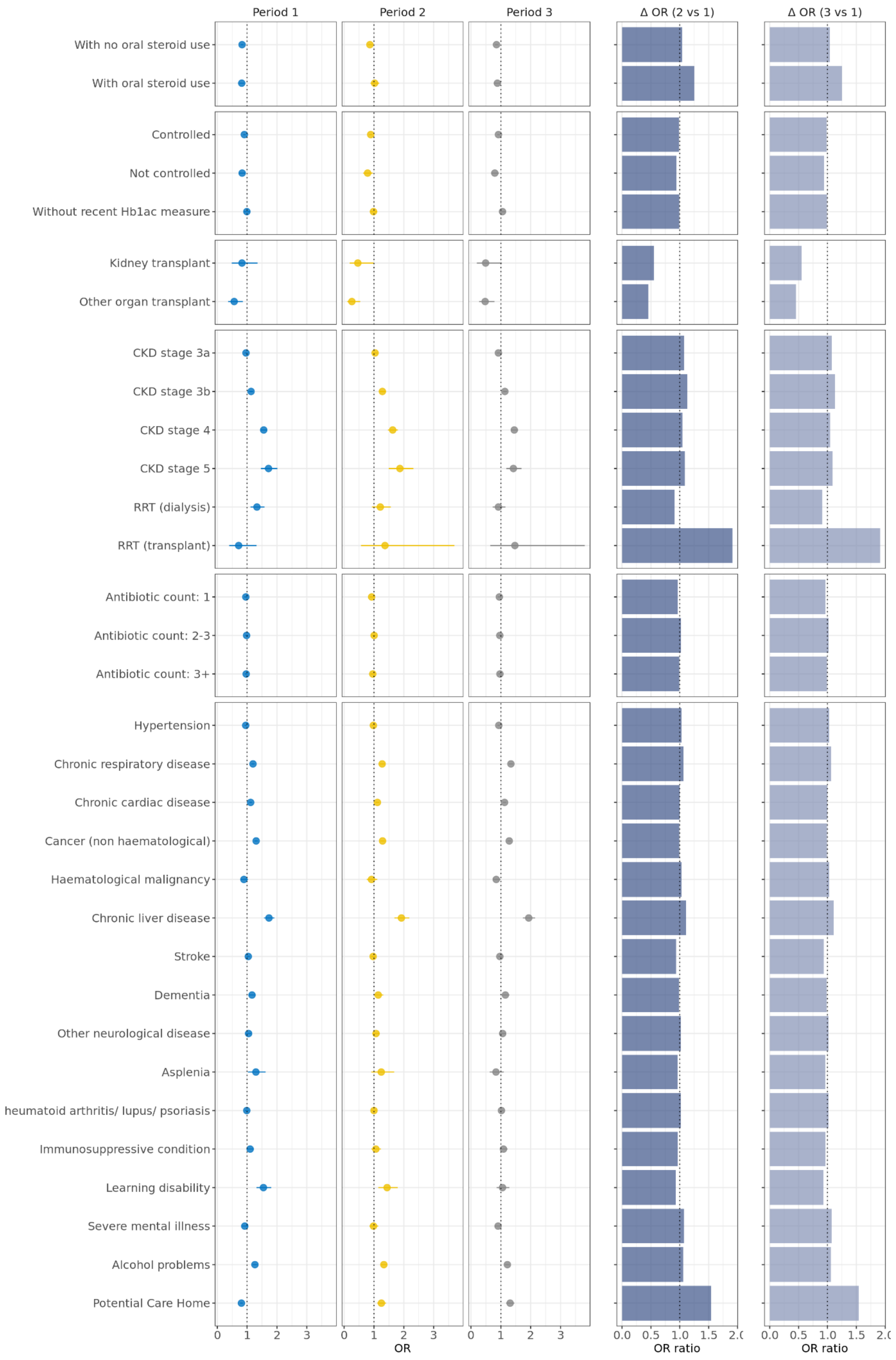
Period 1
Period 2
Period 3
Δ OR (2 vs 1)
Δ OR (3 vs 1)
With no oral steroid use
With oral steroid use
Controlled
Not controlled
Without recent Hb1ac measure
Kidney transplant
Other organ transplant
CKD stage 3a
CKD stage 3b
CKD stage 4
CKD stage 5
RRT (dialysis)
RRT (transplant)
Antibiotic count: 1
Antibiotic count: 2-3
Antibiotic count: 3+
Hypertension
Chronic respiratory disease
Chronic cardiac disease
Cancer (non haematological)
Haematological malignancy
Chronic liver disease
Stroke
Dementia
Other neurological disease
Asplenia
heumatoid arthritis/ lupus/ psoriasis
Immunosuppressive condition
Learning disability
Severe mental illness
Alcohol problems
Potential Care Home
0 1 2 3
OR
0.0 0.5 1.0 1.5 2.0
OR ratio

eFigure 13. Fully adjusted ORs of community-acquired sepsis mortality stratified by COVID-19 period

Relative odds ratio of 30-day mortality after sepsis and 95% confidence intervals in OpenSAFELY-TPP in the three periods (Period 1: 2019-01-01 to 2020-03-25; Period 2: 2020-03-26 to 2021-03-08; Period 3: 2021-03-09 to 2022-06-30).

Models were adjusted for age using a 4-knot cubic spline, except for estimation of age group relative odds of 30-day mortality; and adjusted for sex, except for estimation of sex group relative odds of 30-day mortality; and stratified by region, except for IMD group relative odds of 30-day mortality.

The two columns on the right present the ratio of the relative odds of 30-day mortality (fold-change: Δ OR) of period 2 vs 1 and period 3 vs 1.

Abbreviations: CKD, chronic kidney disease; RRT, renal replacement therapy.

The number of prior antibiotic prescriptions (one year plus six weeks to six weeks before the index date)

Reference sub-group:
Patients without the disease were used as the reference for other clinical conditions.

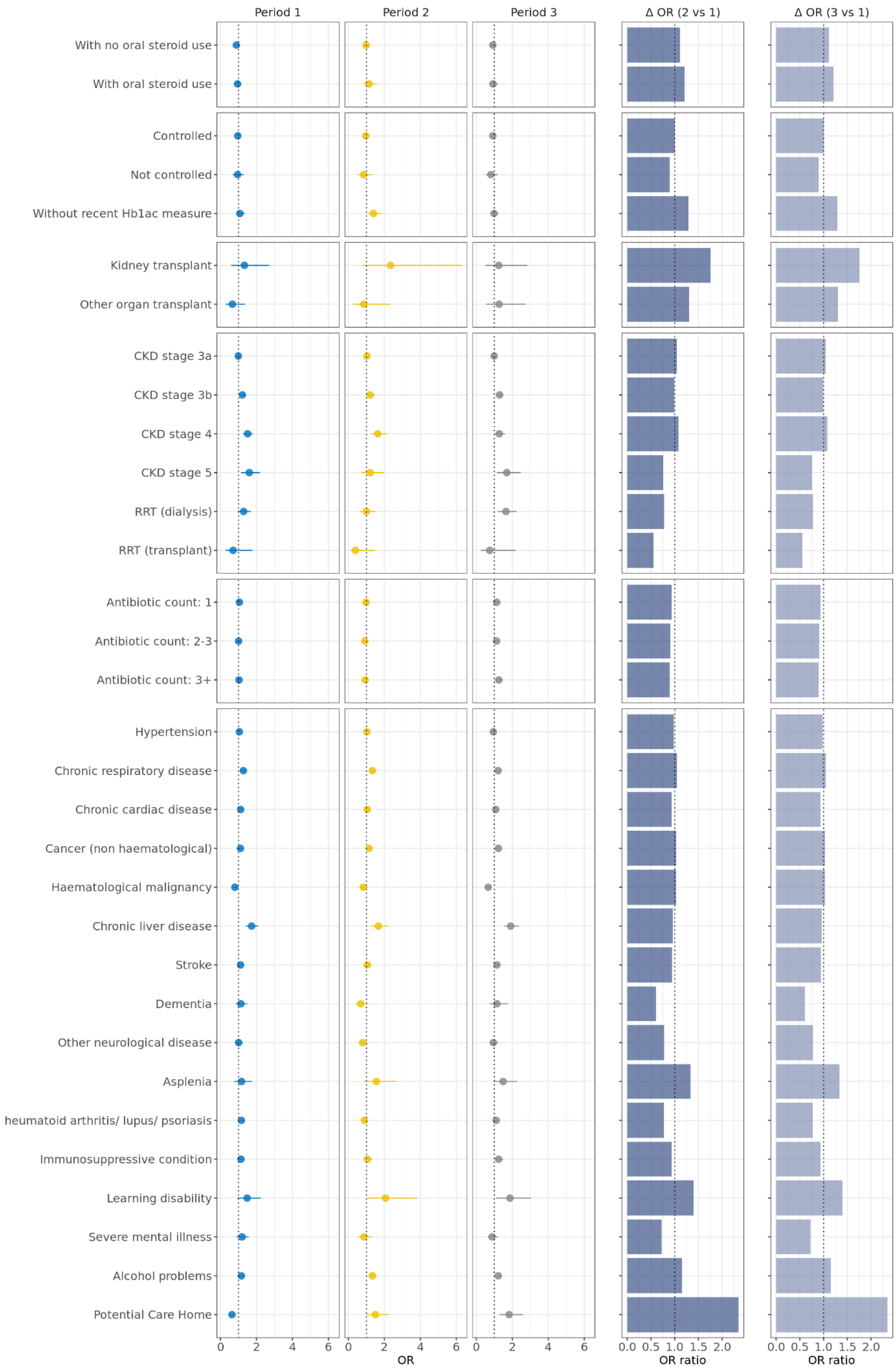
Period 1
Period 2
Period 3
Δ OR (2 vs 1)
Δ OR (3 vs 1)
With no oral steroid use
With oral steroid use
Controlled
Not controlled
Without recent Hb1ac measure
Kidney transplant
Other organ transplant
CKD stage 3a
CKD stage 3b
CKD stage 4
CKD stage 5
RRT (dialysis)
RRT (transplant)
Antibiotic count: 1
Antibiotic count: 2-3
Antibiotic count: 3+
Hypertension
Chronic respiratory disease
Chronic cardiac disease
Cancer (non haematological)
Haematological malignancy
Chronic liver disease
Stroke
Dementia
Other neurological disease
Asplenia
heumatoid arthritis/ lupus/ psoriasis
Immunosuppressive condition
Learning disability
Severe mental illness
Alcohol problems
Potential Care Home
0 2 4 6
OR
0.0 0.5 1.0 1.5 2.0
OR ratio

eFigure 14. Fully adjusted ORs of hospital-acquired sepsis mortality stratified by COVID-19 period

Relative odds ratio of 30-day mortality after sepsis and 95% confidence intervals in OpenSAFELY-TPP in the three periods (Period 1: 2019-01-01 to 2020-03-25; Period 2: 2020-03-26 to 2021-03-08; Period 3: 2021-03-09 to 2022-06-30).

Models were adjusted for age using a 4-knot cubic spline, except for estimation of age group relative odds of 30-day mortality; and adjusted for sex, except for estimation of sex group relative odds of 30-day mortality; and stratified by region, except for IMD group relative odds of 30-day mortality.

The two columns on the right present the ratio of the relative odds of 30-day mortality (fold-change: Δ OR) of period 2 vs 1 and period 3 vs 1.

Abbreviations: CKD, chronic kidney disease; RRT, renal replacement therapy.

The number of prior antibiotic prescriptions (one year plus six weeks to six weeks before the index date)

Reference sub-group:
Patients without the disease were used as the reference for other clinical conditions.

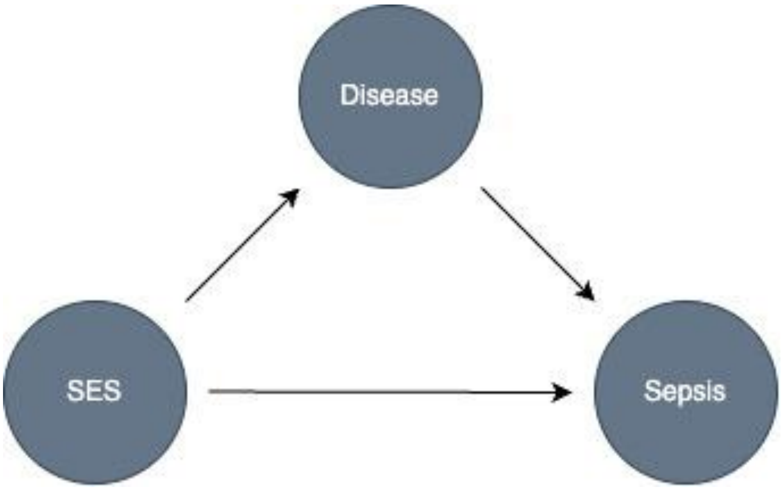

eFigure 15. Directed acyclic graph (DAG) of the association between IMD (socioeconomic status) and the risk of developing non-COVID-19 sepsis

SES: Socioeconomic status
Sepsis: non-COVID-19 sepsis

Reference

1 English indices of deprivation 2019 - GOV.UK. https://www.gov.uk/government/statistics/english-indices-of-deprivation-2019 (accessed 17 June 2023)

2 Identification and classification | Diagnosis | Obesity | CKS | NICE. https://cks.nice.org.uk/topics/obesity/diagnosis/identification-classification/ (accessed 15 March 2023)

3 Moons KGM, Altman DG, Reitsma JB, *et al.* Transparent Reporting of a multivariable prediction model for Individual Prognosis Or Diagnosis (TRIPOD): Explanation and Elaboration. *https://doi.org/107326/M14-0698*. 2015;162:W1–73.

4 Variable reference - OpenSAFELY documentation. https://docs.opensafely.org/study-def-variables/ (accessed 15 March 2023)

5 Russell MD, Galloway JB, Andrews CD, *et al.* Incidence and management of inflammatory arthritis in England before and during the COVID-19 pandemic: a population-level cohort study using OpenSAFELY. *Lancet Rheumatol*. 2022;4:e853–63.

6 NHS Health Check - NHS. https://www.nhs.uk/conditions/nhs-health-check/ (accessed 17 October 2023)

7 Molokhia M, Ayis DS, Karamanos A, *et al.* What factors influence differential uptake of NHS Health Checks, diabetes and hypertension reviews among women in ethnically diverse South London? Cross-sectional analysis of 63,000 primary care records. *EClinicalMedicine*. 2022;49. doi: 10.1016/J.ECLINM.2022.101471

8 Meydan C, Bekenstein U, Soreq H. Molecular regulatory pathways link sepsis with metabolic syndrome: Non-coding RNA elements underlying the sepsis/metabolic cross-talk. *Front Mol Neurosci*. 2018;11:189.

9 Ponsford MJ, Gkatzionis A, Walker VM, *et al.* Cardiometabolic Traits, Sepsis, and Severe COVID-19: A Mendelian Randomization Investigation. *Circulation*. 2020;142:1791.

Supplement:the OpenSAFELY collaborative

alex.walker@phc.ox.ac.uk	bennett institute	University of Oxford	Alex	J	Walker	0000-0003-4932-6135
brian.mackenna@phc.ox.ac.uk	bennett institute	University of Oxford	Brian		MacKenna	0000-0002-3786-9063
peter.inglesby@phc.ox.ac.uk	bennett institute	University of Oxford	Peter		Inglesby	0000-0002-7784-1719
ben.goldacre@phc.ox.ac.uk	bennett institute	University of Oxford	Ben		Goldacre	0000-0002-5127-4728
helen.curtis@phc.ox.ac.uk	bennett institute	University of Oxford	Helen	J	Curtis	0000-0003-3429-9576
jessica.morley@phc.ox.ac.uk	bennett institute	University of Oxford	Jessica		Morley	0000-0001-5221-4770
amir.mehrkar@phc.ox.ac.uk	bennett institute	University of Oxford	Amir		Mehrkar	0000-0002-2098-1278
sebastian.bacon@phc.ox.ac.uk	bennett institute	University of Oxford	Sebastian	C J	Bacon	0000-0002-6354-3454
george.hickman@thedatalab.org	bennett institute	University of Oxford	George		Hickman	0000-0002-6691-4046
richard.croker@phc.ox.ac.uk	bennett institute	University of Oxford	Richard		Croker	0000-0002-8114-9186
david.evans@phc.ox.ac.uk	bennett institute	University of Oxford	David		Evans	0000-0002-1100-079X
thomas.ward@phc.ox.ac.uk	bennett institute	University of Oxford	Tom		Ward	0000-0003-3743-1456
nicholas.devito@phc.ox.ac.uk	bennett institute	University of Oxford	Nicholas	J	DeVito	0000-0001-8286-1995
louis.fisher@phc.ox.ac.uk	bennett institute	University of Oxford	Louis		Fisher	0000-0002-0295-3812
amelia.green@phc.ox.ac.uk	bennett institute	University of Oxford	Amelia	C A	Green	0000-0002-7246-2074
jon.massey@phc.ox.ac.uk	bennett institute	University of Oxford	Jon		Massey	0000-0002-2497-4040
rebecca.smith@phc.ox.ac.uk	bennett institute	University of Oxford	Rebecca	M	Smith	
william.hulme@phc.ox.ac.uk	bennett institute	University of Oxford	William	J	Hulme	0000-0002-9162-4999
simon.davy@thedatalab.org	bennett institute	University of Oxford	Simon		Davy	0000-0001-9890-3619
colm.andrews@phc.ox.ac.uk	bennett institute	University of Oxford	Colm	D	Andrews	0000-0003-2715-0562
Lisa.Hopcroft@phc.ox.ac.uk	bennett institute	University of Oxford	Lisa	E M	Hopcroft	0000-0002-7022-1322
iain.dillingham@phc.ox.ac.uk	bennett institute	University of Oxford	Iain		Dillingham	
rose.higgins@phc.ox.ac.uk	bennett institute	University of Oxford	Rose		Higgins	0000-0002-5295-4370
christine.cunningham@phc.ox.ac.uk	bennett institute	University of Oxford	Christine		Cunningham	0000-0001-6802-7355
milan.wiedemann@phc.ox.ac.uk	bennett institute	University of Oxford	Milan		Wiedemann	0000-0003-1991-282X
linda.nab@phc.ox.ac.uk	bennett institute	University of Oxford	Linda		Nab	0000-0002-1821-7246
steven.maude@phc.ox.ac.uk	bennett institute	University of Oxford	Steven		Maude	0000-0002-6112-5101

orla.macdonald@oxfordhealth.nhs.uk	bennett institute	Oxford Health NHS FT	Orla		Macdonald	0000-0003-2774-0001
benjamin.butler-cole@phc.ox.ac.uk	bennett institute	University of Oxford	Ben	F C	Butler-Cole	
thomas.odwyer@phc.ox.ac.uk	bennett institute	University of Oxford	Thomas		O'Dwyer	0000-0002-4373-1597
catherine.stables@phc.ox.ac.uk	bennett institute	University of Oxford	Catherine	L	Stables	0000-0002-5173-3090
christopher.wood@phc.ox.ac.uk	bennett institute	University of Oxford	Christopher		Wood	0000-0001-5184-0606
andrew.brown@phc.ox.ac.uk	bennett institute	University of Oxford	Andrew	D	Brown	0000-0002-3688-8393
victoria.speed@phc.ox.ac.uk	bennett institute	University of Oxford	Victoria		Speed	000-0001-5064-9375
lucy.bridges@phc.ox.ac.uk	bennett institute	University of Oxford	Lucy		Bridges	0000-0002-4407-9596
andrea.schaffer@phc.ox.ac.uk	bennett institute	University of Oxford	Andrea	L	Schaffer	0000-0002-3701-4997
caroline.walters@phc.ox.ac.uk	bennett institute	University of Oxford	Caroline	E	Walters	0000-0003-1882-0886
chris.bates@tpp-uk.com	TPP	TPP	Christopher		Bates	0000-0003-0113-2593
jonathan.cockburn@tpp-uk.com	TPP	TPP	Jonathan		Cockburn	0000-0002-4681-4873
john.parry@tpp-uk.com	TPP	TPP	John		Parry	
fxhester@tpp-uk.com	TPP	TPP	Frank		Hester	
sam.harper@tpp-uk.com	TPP	TPP	Sam		Harper	
