# Supplementary material for: Clinical and health inequality risk factors for non-COVID-related sepsis during the global COVID-19 pandemic: a national case-control and cohort study
Source: eClinicalMedicine. 2023 Nov 23;66:102321. doi: 10.1016/j.eclinm.2023.102321 (PMC10772239; doi:10.1016/j.eclinm.2023.102321)
Supplement: List of OpenSAFELY Collaborative [file mmc2.docx]

A lit of OpenSAFELY collaborative

| Alex J | Walker |
| --- | --- |
| Brian | MacKenna |
| Peter | Inglesby |
| Ben | Goldacre |
| Helen J | Curtis |
| Jessica | Morley |
| Amir | Mehrkar |
| Sebastian CJ | Bacon |
| George | Hickman |
| Richard | Croker |
| David | Evans |
| Tom | Ward |
| Nicholas J | DeVito |
| Louis | Fisher |
| Amelia CA | Green |
| Jon | Massey |
| Rebecca M | Smith |
| William J | Hulme |
| Simon | Davy |
| Colm D | Andrews |
| Lisa EM | Hopcroft |
| Iain | Dillingham |
| Rose | Higgins |
| Christine | Cunningham |
| Milan | Wiedemann |
| Linda | Nab |
| Steven | Maude |
| Orla | Macdonald |
| Ben FC | Butler-Cole |
| Thomas | O'Dwyer |
| Catherine L | Stables |
| Christopher | Wood |
| Andrew D | Brown |
| Victoria | Speed |
| Lucy | Bridges |
| Andrea L | Schaffer |
| Caroline E | Walters |
| Christopher | Bates |
| Jonathan | Cockburn |
| John | Parry |
| Frank | Hester |
| Sam | Harper |
